# Integrated bioinformatics analysis of IFITM1 as a prognostic biomarker and investigation of its immunological role in prostate adenocarcinoma

**DOI:** 10.3389/fonc.2022.1037535

**Published:** 2022-12-14

**Authors:** Shaoyi Qiao, Wuhe Zhang, Yansheng Su, Yao Jiang

**Affiliations:** Department of Urology, the Air Force 986 Hospital, Shaanxi, China

**Keywords:** prostate cancer, bioinformatics analysis, immune cell infiltration, immune score, IFITM1

## Abstract

**Introduction:**

Prostate adenocarcinoma (PRAD) is a highly aggressive malignancy with high mortality and poor prognosis, and its potential mechanism remains unclear. Our study aimed to identify novel markers for the prognosis of PRAD using bioinformatics technology.

**Methods:**

The GSE32571 dataset was downloaded from the GEO database, and analyzed via the limma R package to identify differentially expressed genes (DEGs) and differentially expressed immune score-related genes (DEISRGs). The immune-related genes (IRGs) were further obtained by overlapping DEISRGs and DEGs, and the core gene was identified via survival analysis. Furthermore, the expression level, prognostic value, and potential functions of the core gene were evaluated via multiple bioinformatics databases.

**Results:**

A total of 301 IRGs were identified from the GSE32571 dataset, and IFITM1 was a down-regulated gene in several types of cancer, including PRAD. Besides, low expression of IFITM1 was associated with a poor prognosis in PRAD. GSEA indicated that the vital pathways of IFITM1-associated genes were mainly enriched in primary immunodeficiency, Th17 cell differentiation, Th1, and Th2 cell differentiation, natural killer cell-mediated cytotoxicity, myeloid dendritic cell activation, regulation of leukocyte activation, etc. Furthermore, IFITM1 was closely correlated with 22 types of tumor-infiltrating immune cells.

**Discussion:**

IFITM1 was a prognostic biomarker for PRAD patients, and it can be acted as a potential immune therapy target in PRAD.

## Introduction

Prostate cancer (PC) is the second most common form of malignancy that impacts men’s health worldwide (1, 2). Prostate-specific antigen is a common diagnostic marker for prostate cancer. However, it has low specificity in differentiating aggressive disease from indolent disease, leading to frequent overtreatment and overdiagnosis (3). Recently, although some advances in chemotherapy, radiation, surgery, and diagnostic imaging have effectively enhanced the diagnosis, treatment, and management of prostate cancer (4, 5), some of the therapies are difficult to achieve the effect of radical resection, and due to its high heterogeneity, prostate cancer presents significant challenges in diagnosis and prognosis (6). In addition, the detailed mechanisms of tumorigenesis and metastasis are still unclear, and a novel strategy for prostate cancer care and diagnosis is urgently needed.

With the development of genomics research, high-throughput sequencing and microarray techniques have been widely applied to screen potential mechanisms and differentially expressed genes implicated in the occurrence and progression of human diseases, including cancer (7, 8). These techniques also provided an effective approach to screening potential biological markers for diagnosis and prognosis (9, 10). In addition, the comprehensive application of bioinformatics approaches could solve the errors caused by small sample sizes or different technology platforms, to find a lot of valuable biological information (11, 12).

In the present study, the gene expression profiles of the GSE32571 dataset were analyzed to assess the infiltrate level of immune cells through the xCell method. Then, the GSE32571 dataset of samples was divided into the low-immune score and high-immune score subgroups based on the median values of the immune score. We identified that IFITM1 was closely associated with the overall survival in PRAD *via* integrated bioinformatics analysis. In addition, we also comprehensively assessed the relationship between IFITM1 expression and the prognosis of PRAD patients through TCGA and GEO databases. The association between IFITM1 and immune cell infiltrations was further assessed using the ssGSEA and Tumor Immunity Estimation Resource (TIMER). Our findings aimed at providing a potential prognostic biomarker for PRAD.

## Methods

### Data source and data acquisition

The gene expression profiles of GSE32571 (including 39 normal samples and 59 tumor samples), GSE32448 (including 40 normal samples and 40 tumor samples), and GSE46602 (including 14 normal samples and 36 tumor samples) were downloaded from the GEO. We also downloaded the mRNA expression data (52 adjacent non-tumor samples and 499 PRAD samples) and clinical information of PRAD patients from TCGA database. In addition, we collected the complete clinical and survival information of 138 PC patients from the Memorial Sloan Kettering Cancer Center (MSKCC) for prognostic marker validation.

### Data processing of differently expressed genes

The GSE32571 dataset was normalized using the Linear Models for Microarray Data (limma) package of R. The limma package was also applied to identify the differently expressed genes (DEGs) between the normal and tumor groups, and genes with p < 0.05 and absolute log2 fold change > 0.5 were identified as DEGs. The immune score of each sample was calculated by using the Estimation of Stromal and Immune cells in MAlignant Tumours using Expression data (ESTIMATE) algorithm based on the GSE32571 dataset. In addition, to identify the immune-related genes, the GSE32571 dataset of samples was divided into the low-immune score and high-immune score subgroups based on the median values of the immune score. Differentially expressed immune score-related genes (DEISRGs) were identified between low-immune score and high-immune score subgroups using the limma package. p < 0.05 and absolute log2 fold change > 0.5 were used as the screening parameters. DEGs and DEISRGs were visualized by drawing volcano maps and heatmaps using the ggplot and heatmap packages of R, respectively. Differentially expressed immune-related genes (DEIRGs) were obtained by the intersection of DEGs and DEISRGs.

### Assessment of immune cell infiltration

xCell is a robust algorithm based on ssGSEA that analyzes the infiltration levels of 64 immune and stroma cell types, such as extracellular matrix cells, epithelial cells, hematopoietic progenitors, and innate and adaptive immune cells (13). In the present study, we used the xCell method to assess the infiltration levels between low-immune score and high-immune score subgroups.

### PPI network and module analysis

The PPI network of DEIRGs was constructed *via* the online tool STRING (http://string-db.org). The results were visualized *via* Cytoscape (version 3.6.1) software. In addition, we used the molecular complex detection (MCODE) plug-in of Cytoscape to identify the significant modules and densely connected regions (14). The criteria of clustering and scoring were set as follows: k-score = 2, max depth = 100, node score cutoff = 0.2, degree cutoff = 2, and MCODE score > 3.

### Analysis of IFITM1 expression levels in human cancers

The “DiffExp module” of TIMER was applied to analyze the IFITM1 expression level in normal tissues and various cancers. We also performed expression analysis on both unpaired and paired samples based on TCGA-PRAD database. We performed the ROC analysis to assess the diagnostic value of IFITM1 in PRAD patients.

### Assessment of association between clinical characteristics and IFITM1 expression in PRAD

PRAD patients were divided into low and high IFITM1 expression groups based on the median value of IFITM1. The association between clinical characteristics and IFITM1 expression was assessed by using Wilcoxon’s rank-sum test, Fisher’s test, and Chisq test.

### Survival analysis for IFITM1

PRAD patients were divided into low and high IFITM1 expression groups based on the median value of IFITM1. The Kaplan–Meier method with the log-rank test was applied to compare the overall survival for different IFITM1 patterns.

### Analysis of IFITM1 co-expression genes in TCGA-PRAD cohort

The LinkFinder module of the LinkedOmics database was applied to identify the IFITM1’s co-expression genes in TCGA-PRAD cohort. The correlation between IFITM1 and its co-expression genes was evaluated *via* the Pearson correlation coefficient, and the results were visualized by a volcano map and heat map. The LinkInterpreter module of the LinkedOmics platform was used to carry out the Gene Set Enrichment Analysis (GSEA) to obtain the GO-BP and KEGG pathways potentially regulated by IFITM1 in PRAD (15).

### Immune cell infiltrate analysis

The GSVA package of R was used to carry out ssGSEA and compare the enrichment score of 24 immune cell types between the IFITM1 low and high expression groups. TIMER was used to analyze the correlation between IFITM1 expression and the infiltration level of B cell, CD8 T cell, CD4 T cell, macrophage, neutrophil, and dendritic cell. The correlation between IFITM1 expression and immune checkpoint gene expression was visualized *via* co-expression heat maps based on TCGA-PRAD database.

### PC cell culture and tissue sample

Human normal prostate epithelial cell lines (RWPE-1) and human PC cell lines (DU145, PC3, 22RV1, LNCaP, and VCaP) were purchased from the American Type Culture Collection. The cell lines were cultured in RPMI-1640 medium (Gibco, United States). All mediums were supplemented with 1% complex of streptomycin and penicillin and 10% fetal bovine serum (Gibco, United States) at 37°C in a 5% CO_2_ atmosphere. We collected the carcinoma tissues (n = 10) and corresponding noncancerous normal samples (n = 10) from 10 PC patients who underwent tumor resection in the Air Force 986 Hospital between 2020 and 2021. This study was approved by the Ethics Committee of the Air Force 986 Hospital.

### Quantitative reverse real-time PCR

TRIzol reagent (Invitrogen, USA) was used to perform total RNA extraction. Two micrograms of purified RNA was used to synthesize cDNA by using RevertAid First Strand cDNA Synthesis Kit (Thermo Fisher, United States) according to the manufacturer’s instruction. Then, quantitative reverse real-time PCR (qRT-PCR) was performed in the LightCycler 480 System (Roche, United States) using Fast SYBR Green Master Mix (Roche, United States). The 2^–ΔΔCt^ method was used to quantify the mRNA expression levels of IFITM1. The primer sequences are listed in **Supplementary Table S1**.

### Statistical analysis

R software (version 3.3.3) was used to perform all statistical data derived from TCGA database. The differences between tumor samples and adjacent non-cancer samples were compared by Wilcoxon signed-rank test and Mann–Whitney U-test. The association between IFITM1 expression and clinical features was assessed by the Wilcoxon rank-sum test. We used GraphPad Prism to conduct statistical analysis. The statistical significance between the two groups was evaluated by Student’s t-test. P-value < 0.05 was regarded as statistically significant.

## Results

### Identification of DEGs in the GEO dataset

As shown in **Supplementary Figure S1A**, a total of 1,424 DEGs were identified between normal and prostate cancer groups, of which 910 were low expression genes and 514 were high expression genes. The heatmap of the top 50 DEGs is shown in **Supplementary Figure S1B**.

### Characterization of the immune cell infiltrations in immune score subgroups

As shown in **Supplementary Figure S2A**, the result of the ESTIMATE algorithm indicated that PC patients had lower immune scores compared with normal individuals (p < 0.01). Next, the samples in the GSE32571 dataset were divided into high- and low-immune score subgroups based on the median values of the immune score. Subsequently, hierarchical cluster analysis was carried out based on the immune cell infiltration results obtained by the xCell method (**Supplementary Figure S2B**). As shown in **Supplementary Figures S2C, D**, aDC, B cells, CD4 memory T cells, CD4 naive T cells, CD4 T cells, CD4 Tem, CD8 T cells, CD8 Tcm, CD8 Tem, cDC, macrophages, macrophages M1, memory B cells, NK cells, NKT, pDC, Th2 cells, and microenvironment score were highly increased in the high immune score group. These findings revealed that the immune cell patterns were significantly different between the high- and low-immune score groups. Thus, we intend to perform the differential analysis between the two groups to identify immune-related differential genes.

### Identification of DEISRGs in the GEO dataset

As shown in **Supplementary Figure S3A**, a total of 484 DEISRGs were identified between high- and low-immune score groups, of which 130 were low-expression genes and 354 were high-expression genes. The heatmap of the top 50 DEISRGs is shown in **Supplementary Figure S3B**.

### Identification of prognosis-associated DEIRGs in the GEO dataset

**Figure 1:**
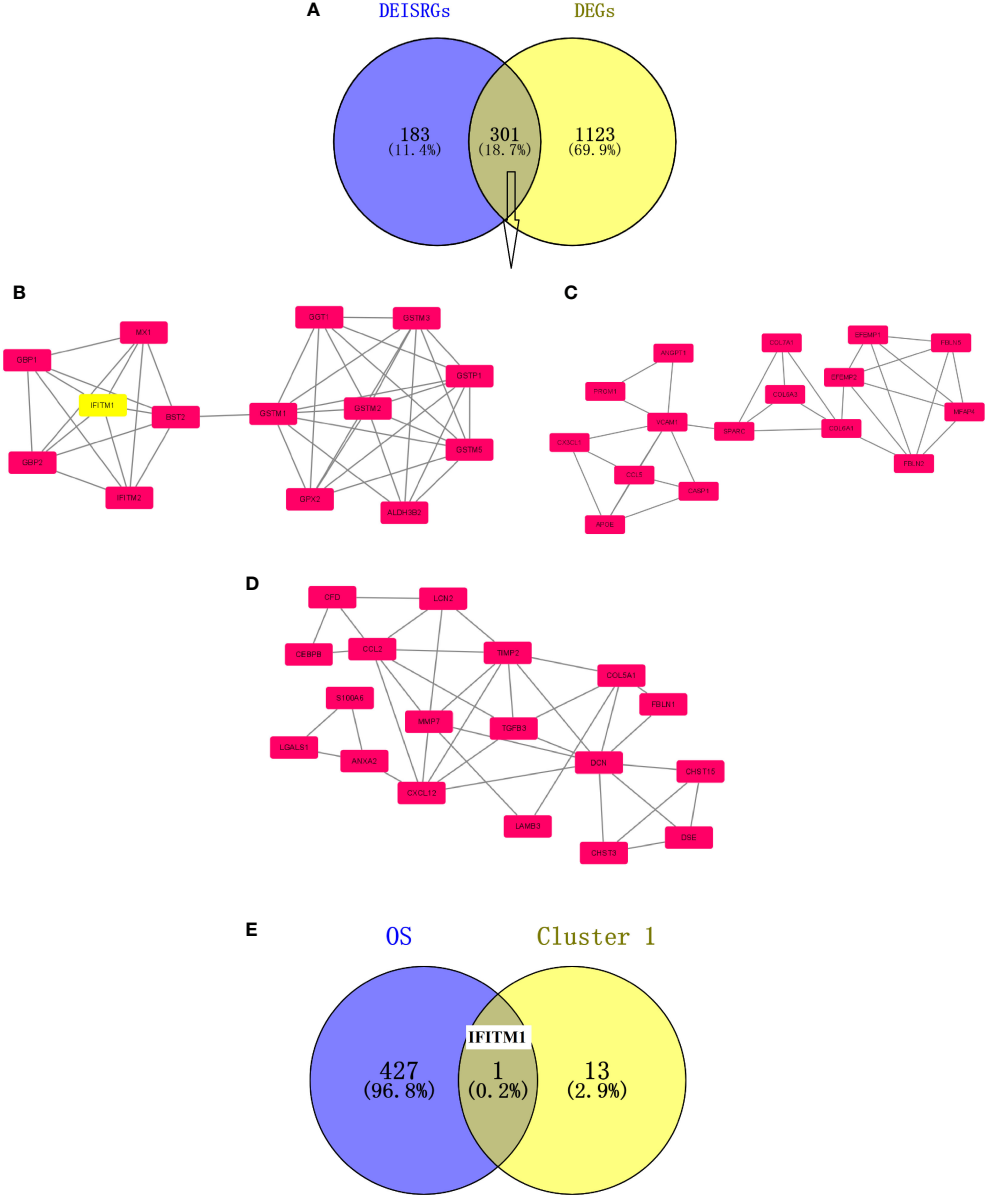
Screening of prognosis-associated DEIRGs. **(A)** Venn diagram of the 301 DEIRGs. **(B)** Cluster 1 with an MCODE score of 6.462. **(C)** Cluster 2 with an MCODE score of 4.353. **(D)** Cluster 3 with an MCODE score of 4.133. The higher the MCODE score, the more important the cluster is in the PPI network. **(E)** Venn diagram of prognosis-associated DEIRGs. The overlapping gene is IFITM1.

A total of 301 overlapping DEIRGs were obtained between DEGs and DEISRGs using a Venn tool (**Figure 1**). Next, the PPI network of 301 DEIRGs was constructed *via* the STRING database, and the MCODE plug-in was used to generate the crucial clustering modules of the PPI network. As shown in **Figures 1B–D**, three clusters were identified: cluster 1 contained 14 nodes and 42 edges with an MCODE score of 6.462; cluster 2 contained 18 nodes and 37 edges with an MCODE score of 4.353; and cluster 3 contained 16 nodes and 31 edges with an MCODE score of 4.133. In addition, survival analysis indicated that there were 428 genes related to the overall survival of PC patients in TCGA dataset. Subsequently, the core prognosis-associated gene (IFITM1) was obtained between cluster 1 and 428 prognosis-associated genes using a Venn tool (**Figure 1E**).

### Analysis of IFITM1 expression in cancer samples

**Figure 2:**
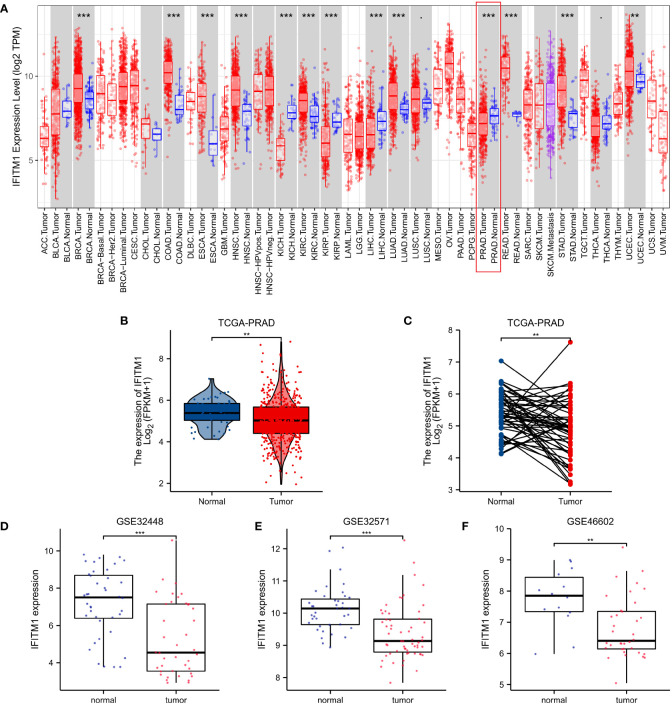
The IFITM1 expression levels in human cancers. **(A)** IFITM1 expression in pan-cancer based on the TIMER database. **(B)** IFITM1 expression in TCGA-PRAD was measured *via* unpaired sample analysis. **(C)** IFITM1 expression in TCGA-PRAD was measured *via* paired sample analysis. IFITM1 expression in GSE32448 **(D)**, GSE32571 **(E)**, and GSE46602 **(F)** datasets. **p < 0.01, and ***p < 0.001.

Based on the TIMER database (**Figure 2A** **)**, IFITM1 was found to be significantly upregulated in various tumors, including breast invasive carcinoma (BRCA), colon adenocarcinoma (COAD), esophageal carcinoma (ESCA), head and neck squamous cell carcinoma (HNSC), kidney chromophobe (KIRC), lung adenocarcinoma (LUAD), rectum adenocarcinoma (READ), stomach adenocarcinoma (STAD), and uterine corpus endometrial carcinoma (UCEC). However, IFITM1 was significantly downregulated in some malignancies, such as kidney chromophobe (KICH), kidney renal clear cell carcinoma (KIRP), liver hepatocellular carcinoma (LIHC), and prostate adenocarcinoma (PRAD). We also prove the significantly downregulated IFITM1 gene expression in PRAD patients based on GEO and TCGA databases (**Figures 2B** **–F**).

### Relationships between clinicopathologic features and IFITM1 expression in PRAD

As shown in **Supplementary Figure S4** and **Table 1**, the IFITM1 expression was significantly correlated with M stage, N stage, T stage, PSA, Gleason score, primary therapy outcome, residual tumor, DSS event, and OS event. In addition, we performed the ROC analysis to evaluate the diagnostic value of IFITM1 in PRAD patients, and the results showed that IFITM1 could differentiate PRAD samples from the normal samples (**Supplementary Figure S5**, AUC = 0.750).

**Table 1:** Analysis of clinical features of TCGA-PRAD patients based on IFITM1 expression.

Characteristic	Low expression of IFITM1	High expression of IFITM1	p
n	247	248	
T stage, n (%)			0.097
T2	105 (21.5%)	82 (16.8%)	
T3	136 (27.9%)	155 (31.8%)	
T4	4 (0.8%)	6 (1.2%)	
N stage, n (%)			**0.022**
N0	180 (42.7%)	164 (38.9%)	
N1	29 (6.9%)	49 (11.6%)	
M stage, n (%)			1.000
M0	227 (49.8%)	226 (49.6%)	
M1	2 (0.4%)	1 (0.2%)	
Primary therapy outcome, n (%)			0.247
PD	15 (3.5%)	13 (3%)	
SD	14 (3.2%)	15 (3.5%)	
PR	14 (3.2%)	26 (6%)	
CR	174 (40.1%)	163 (37.6%)	
Race, n (%)			0.857
Asian	5 (1%)	7 (1.5%)	
Black or African American	28 (5.8%)	28 (5.8%)	
White	205 (42.7%)	207 (43.1%)	
Age, n (%)			0.224
<=60	118 (23.8%)	104 (21%)	
>60	129 (26.1%)	144 (29.1%)	
Residual tumor, n (%)			0.143
R0	165 (35.5%)	149 (32%)	
R1	63 (13.5%)	83 (17.8%)	
R2	3 (0.6%)	2 (0.4%)	
Zone of origin, n (%)			0.370
Central zone	3 (1.1%)	1 (0.4%)	
Overlapping/multiple zones	61 (22.3%)	65 (23.7%)	
Peripheral zone	65 (23.7%)	71 (25.9%)	
Transition zone	6 (2.2%)	2 (0.7%)	
PSA (ng/mL), n (%)			0.399
<4	210 (47.9%)	201 (45.9%)	
≥4	11 (2.5%)	16 (3.7%)	
Gleason score, n (%)			0.069
6	25 (5.1%)	20 (4%)	
7	131 (26.5%)	115 (23.2%)	
8	35 (7.1%)	28 (5.7%)	
9	55 (11.1%)	82 (16.6%)	
10	1 (0.2%)	3 (0.6%)	
OS event, n (%)			**0.011**
Alive	238 (48.1%)	247 (49.9%)	
Dead	9 (1.8%)	1 (0.2%)	
DSS event, n (%)			**0.030**
Alive	241 (48.9%)	247 (50.1%)	
Dead	5 (1%)	0 (0%)	
PFI event, n (%)			1.000
Alive	201 (40.6%)	201 (40.6%)	
Dead	46 (9.3%)	47 (9.5%)	

Bold values means P-value < 0.05 was regarded as statistically significant.

**Figure 3:**
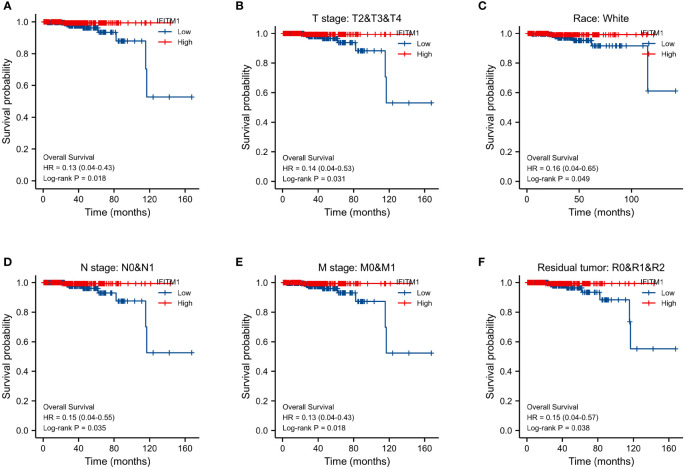
Assessment of IFITM1’s prognostic value in PRAD. **(A)** The overall survival in all PRAD patients. The overall survival for T2&T3&T4 **(B)**, white **(C)**, N0&N1 **(D)**, M0&M1 **(E)**, and R0&R1&R2 **(F)** subgroups.

Furthermore, we used the survminer package of R to assess the prognostic value of IFITM1 in PRAD patients. Our results showed that the downregulation of IFITM1 expression was associated with poor overall survival (p = 0.018) (**Figure 3A**). Moreover, the low expression of IFITM1 was also related to worse overall survival in the T stage (p = 0.031), the white subgroup of the race (p = 0.049), N stage (p = 0.035), M stage (p = 0.018), and residual tumor (p = 0.038) (**Figures 3B** **–F**).

### Pan-cancer analysis of the prognostic value of IFITM1

We assessed the prognostic value of IFITM1 in multiple types of cancer. As shown in **Supplementary Figure S6**, Kaplan–Meier survival analysis revealed that the low expression of IFITM1 was associated with poor overall survival in BRCA (p = 0.031); the upregulation of IFITM1 expression was associated with poor overall survival in KIRC (p = 0.005) and KIRP (p = 0.016). However, IFITM1 is upregulated in BRCA and KIRC, and IFITM1 is downregulated in KIRP (**Figure 2A**). Overall, high expression of IFITM1 is associated with poor overall survival in KIRC and is a potential prognostic biomarker for KIRC patients.

### Investigation of the co-expression pattern of IFITM1 in TCGA-PRAD cohort

**Figure 4:**
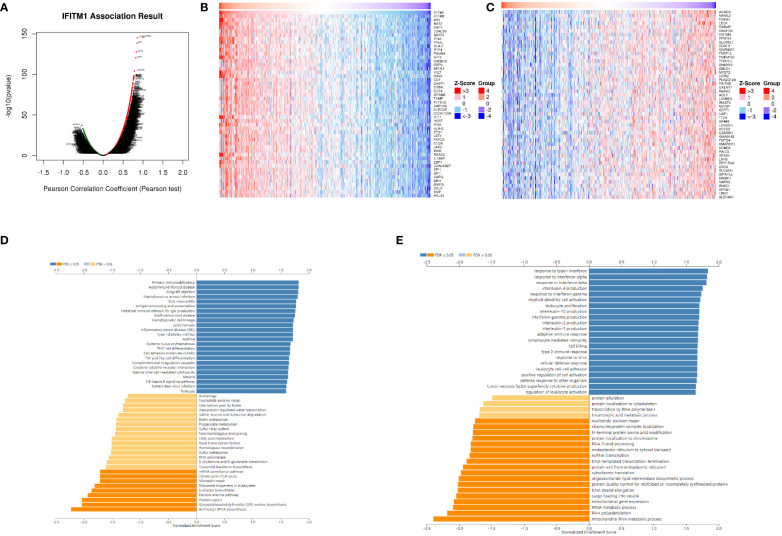
The co-expression pattern of IFITM1 in TCGA-PRAD cohort. **(A)** Volcano map of IFITM1 co-expression genes. **(B)** IFITM1 expression was positively related to the top 50 co-expression genes **(B)** and negatively related to the top 50 co-expression genes **(C)**. Potential KEGG **(D)** and GO-BP pathways **(E)** are medicated by IFITM1 in PRAD.

We identified that 4,956 genes (green dots) were negatively correlated with IFITM1, whereas 8,306 genes (red dots) were positively correlated with IFITM1 using the LinkedOmics database (**Figure 4A**). In addition, the top 50 positively correlated co-expressed genes are shown in **Figure 4B**, whereas the top 50 negatively correlated co-expressed genes are presented in **Figure 4C**.

Furthermore, KEGG enrichment analysis showed these co-expressed genes of IFITM1 mainly enriched in primary immunodeficiency, autoimmune thyroid disease, the intestinal immune network for IgA production, inflammatory bowel disease, Th17 cell differentiation, cytokine–cytokine receptor interaction, natural killer cell-mediated cytotoxicity, and NF-kappa B signaling pathway (**Figure 4D**). GO-BP functional enrichment analysis revealed these IFITM1 co-expression genes mainly enriched in response to interferon-alpha, interleukin-4 production, response to interferon-gamma, myeloid dendritic cell activation, leukocyte proliferation, interleukin-10 production, adaptive immune response, lymphocyte-mediated immunity, cell killing, type 2 immune response, leukocyte cell–cell adhesion, positive regulation of cell activation, tumor necrosis factor superfamily cytokine production, and regulation of leukocyte activation (**Figure 4E**).

As shown in **Supplementary Figure S7A**, a PPI network of these co-expression genes with 64 nodes and 224 edges was constructed. Cluster 1 contained 17 nodes and 130 edges with an MCODE score of 16.25 (**Supplementary Figure S7B**). Cluster 2 contained three nodes and three edges with an MCODE score of 3 (**Supplementary Figure S7C**). **Supplementary Figure S8** displayed the expression levels of genes (cluster 1) in TCGA-PRAD cohort. Among them, BST2, GBP2, HLA-E, IFIT1, XAF1, RTP4, MX1, IFITM3, and IFIT3 were significantly decreased in PRAD. We also performed a prognostic analysis of these co-expression genes. However, none of them have prognostic value for PRAD patients.

### Investigation of the correlation between IFITM1 expression and immune cell infiltration

**Figure 5:**
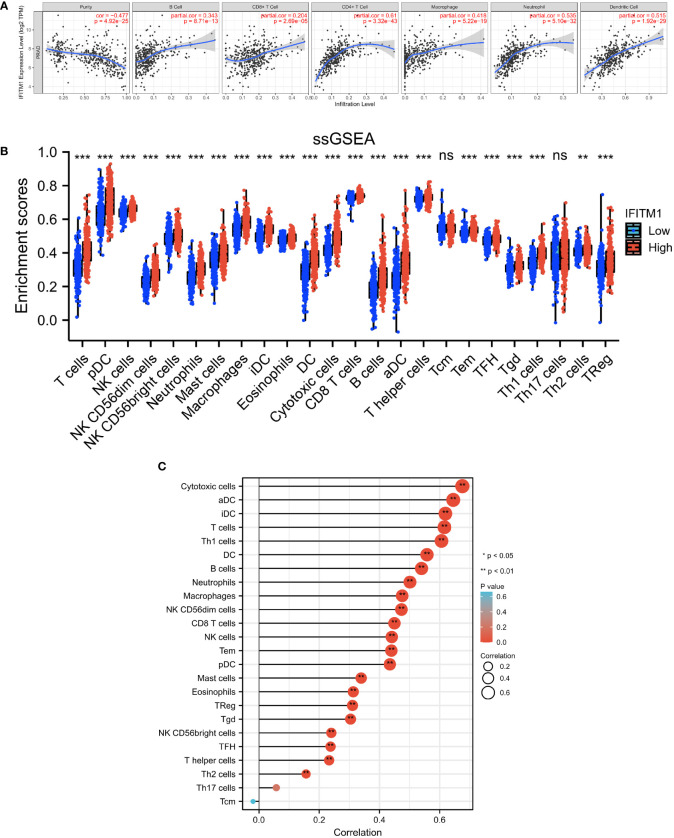
Investigation of the correlation between immunity and IFITM1 expression in PRAD. **(A)** Correlation analysis between IFITM1 expression and six immune cell subtypes level in the TIMER database. **(B)** Enrichment scores of 24 immune cell subtypes in PRAD patients with low IFITM1 expression and high IFITM1 expression. **(C)** Correlation analysis between IFITM1 expression and 24 immune cell subtypes level in TCGA-BRCA database. ***p < 0.001, "ns" is no significant difference.

We used TIMER and ssGSEA to further explore the correlation between IFITM1 expression and immunity. As shown in **Figure 5A**, IFITM1 expression was a positive correlation with B cell, CD8 T cell, CD4 T cell, macrophage, neutrophil, and dendritic cell. In addition, the enrichment scores of T cells, pDC, NK cell, NK CD56dim cells, NK CD56bright cells, neutrophils, mast cells, macrophages, iDC, eosinophils, DC, cytotoxic cells, CD8 T cells, B cells, aDC, T helper cells, Tem, TFH, Tgd, Th1 cells, Th2 cells, and Treg were decreased in the low-expression IFITM1 group compared with those in the high-expression of IFITM1 group (**Figure 5B**). IFITM1 expression was positively correlated with these immune cells (**Figure 5C**).

The relationship between IFITM1 expression and immune cell-related immunoinhibitors and immunostimulators in PRAD was also analyzed. As presented in **Supplementary Figure S9A**, 40 immunostimulator genes were positively correlated with IFITM1, including CD27, CD28, CD40, CD40LG, CD48, CD70, CD80, CD86, CXCL12, CXCR4, ENTPD1, HHLA2, ICOS, ICOSLG, IL2RA, IL6, IL6R, KLRC1, KLRK1, LTA, MICB, NT5E, RAET1E, TMIGD2, TNFRSF13B, TNFRSF13C, TNFRSF14, TNFRSF17, TNFRSF18, TNFRSF25, TNFRSF4, TNFRSF8, TNFRSF9, TNFSF13, TNFSF13B, TNFSF14, TNFSF15, TNFSF18, TNFSF9, and ULBP1. Twenty-four immunoinhibitor genes were positively correlated with IFITM1, such as ADORA2A, BTLA, CD160, CD244, CD274, CD96, CSF1R, CTLA4, HAVCR2, IDO1, IL10, IL10RB, KDR, KIR2DL1, KIR2DL3, LAG3, LGALS9. PDCD1, PDCD1LG2, TGFB1, TGFBR1, TIGIT, and VTCN1 (**Supplementary Figure S9B** **)**.

### Validation of IFITM1 expression and its prognostic value in PC

**Figure 6:**
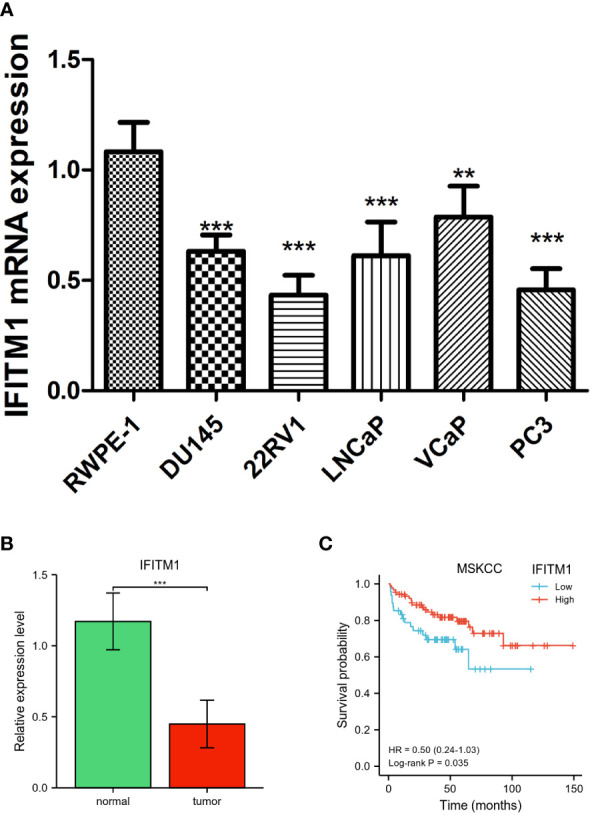
Validation of IFITM1 in PC. **(A)** IFITM1 mRNA expression in PC cell lines validated by qRT-PCR. **(B)** The mRNA expression of IFITM1 in PC patients. **(C)** Kaplan–Meier curves of overall survival for low- and high-IFITM1 subgroups in MSKCC-PRAD. **p < 0.01 and ***p < 0.001.

As shown in **Figure 6A**, we observed that IFITM1 expression levels were downregulated in PC cells (DU145, PC3, 22RV1, LNCaP, and VCaP) compared with RWPE-1 cells (p < 0.001). Similar expression results were found in clinical PC patients (**Figure 6B**). In addition, the low expression of IFITM1 was associated with poor overall survival in MSKCC-PRAD (**Figure 6C**). These results were consistent with our bioinformatics analysis findings.

## Discussion

Human interferon-induced transmembrane proteins (IFITMs) are a family of small homologous proteins and are involved in various physiological processes, including bone formation, tumor inhibition, and antiviral immunity (16). Recent studies have revealed that IFITMs play an important role in adaptive immunity, impacting Th2 immunopathology and Th1/Th2 polarization (17). IFITMs are involved in the oncogenesis of kidney renal clear cell carcinoma and may be a prognosis biomarker for treatment response to targeted therapies (18). Therefore, IFITMs can be the potential targets for tumor treatment (19, 20).

In the present study, we identified IFITM1 as a novel prognostic marker for PRAD patients using an integrated bioinformatics approach. Our findings showed that IFITM1 expression was downregulated in PRAD tumor samples. As an important member of the IFITM family, human interferon-induced transmembrane protein 1 (IFITM1) has been revealed to be a modulator of antiviral activity and immunity (21). It has also been reported to be anomalously expressed in cancer cell lines as well as tumor samples (22), including colorectal cancer (23), gastric cancer (24), liver cancer (25), lung cancer (26), breast cancer (27), head and neck cancer (28), glioma (29), and ovarian cancer (30). In our study, we also found that low expression of IFITM1 was related to a poor prognosis for PRAD patients. Previous studies have indicated that IFITM1 is an independent prognostic marker for cancer patients. For example, IFITM1 is a prognostic marker in resected esophageal and gastric adenocarcinoma (31). IFITM1 expression is a biomarker for the diagnosis, poor prognosis, and clinical severity of gallbladder carcinomas (32). IFITM1 is closely correlated with angiogenesis, and it may be a potential biomarker for lung adenocarcinoma patients (33). These findings indicated that IFITM1 could be a novel therapeutic target for effective tumor therapy.

IFITM1 is an immune-related IFITM and has been reported to inhibit the early replication of multiple viruses (34). IFITM1 expression was associated with immune activation in CD4 T cells (35). In this study, we also performed GSEA to further investigate the functions and mechanisms of IFITM1 in PRAD. The results of GSEA revealed that positively enriched KEGG pathways and GO-BP terms were immune-related pathways, including primary immunodeficiency, the intestinal immune network for IgA production, Th17 cell differentiation, cytokine–cytokine receptor interaction, natural killer cell-mediated cytotoxicity, myeloid dendritic cell activation, leukocyte proliferation, adaptive immune response, lymphocyte-mediated immunity, cell killing, type 2 immune response, leukocyte cell–cell adhesion, positive regulation of cell activation, regulation of leukocyte activation, etc. All these results implied that IFITM1 may be involved in the immune microenvironment to improve the prognosis of PRAD patients.

Tumor-infiltrating immune cells are an important part of the tumor microenvironment and participate in the occurrence and progression of tumors (36–38). Accumulating evidence revealed that myeloid-derived cells play a vital role in the promotion and progression of prostate cancer (39). The immune cell infiltration phenotypes are closely associated with the adverse prognosis for prostate cancer patients (40). A recent study indicated higher infiltrating neutrophils and M1 macrophages in prostate cancer tissues, and these immune cells may be potential targets in the diagnosis and prognosis of prostate cancer (41). Infiltrating macrophages and regulatory T cells were identified as adverse prognostic factors in prostate cancer (42). In the present study, we found that IFITM1 was positively correlated with most tumor-infiltrating immune cells using TIMER and ssGSEA. Furthermore, IFITM1 expression was positively correlated with the immune cell-related immunoinhibitors and immunostimulators in PRAD. To our knowledge, there was no study exploring the association between IFITM1 and immune cell infiltration in cancer; therefore, our results provided a novel research direction for tumor immunity study. Our results implied that there was a potential relationship between immune cell infiltration and IFITM1 in PRAD. However, more experiments were needed to verify this correlation.

## Conclusion

Our results indicated that low expression of IFITM1 was an adverse prognostic factor in PRAD. IFITM1 might play an important role in the tumor immune microenvironment of PRAD *via* the regulation of tumor-infiltrating immune cells. Our findings highlighted a potential function of IFITM1 and its therapeutic potential for PRAD patients.

## Data availability statement

The original contributions presented in the study are included in the article/**Supplementary Material**. Further inquiries can be directed to the corresponding author.

## Author contributions

SQ drafted the manuscript. YJ was responsible for the acquisition of data. YS participated in the data analysis. WZ modified the manuscript. All authors contributed to the article and approved the submitted version.
