# Supplementary figures and images for: Integrated bioinformatics analysis of IFITM1 as a prognostic biomarker and investigation of its immunological role in prostate adenocarcinoma
Source: Front Oncol. 2022 Dec 14;12:1037535. doi: 10.3389/fonc.2022.1037535 (PMC9795034; doi:10.3389/fonc.2022.1037535)

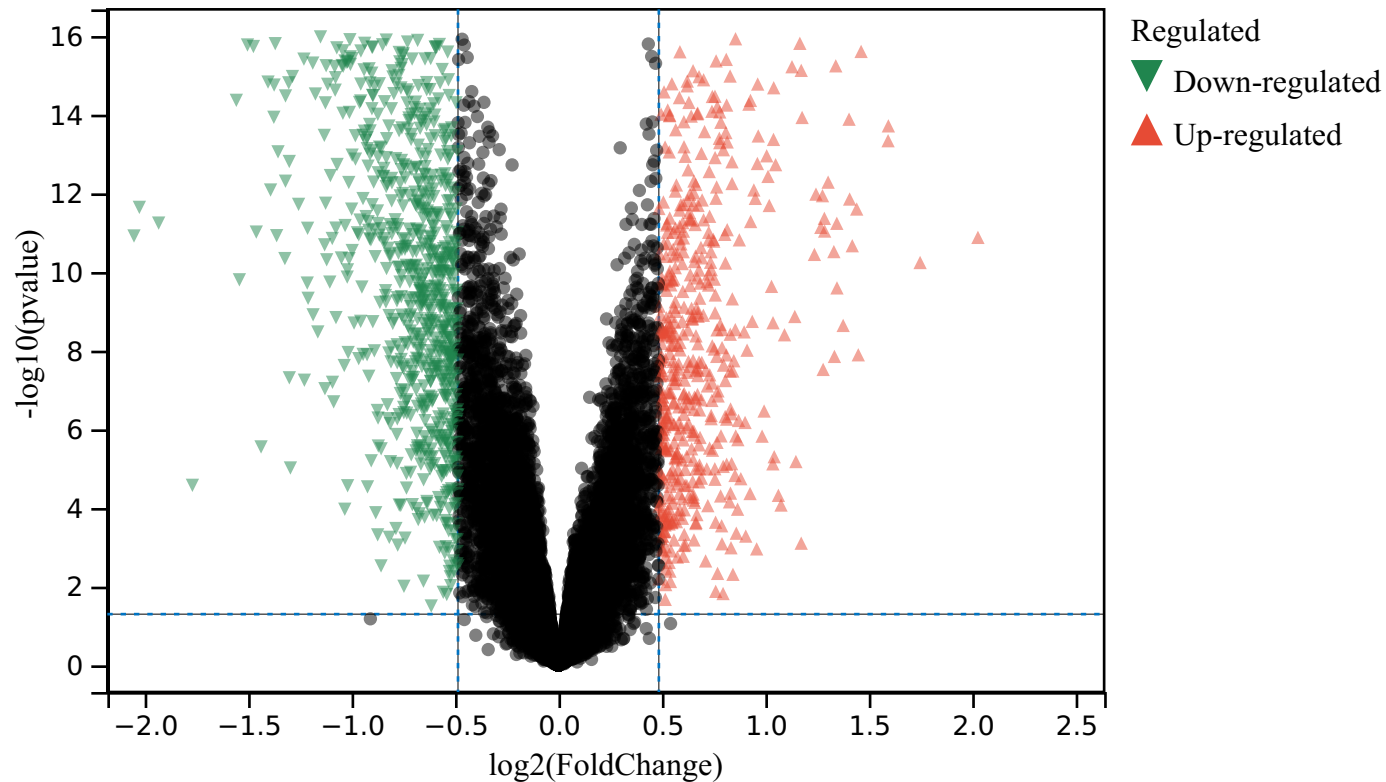

Supplement: Supplementary file 1 [file DataSheet_1.zip › raw data 1/Fig 1/Fig 1a.pdf]

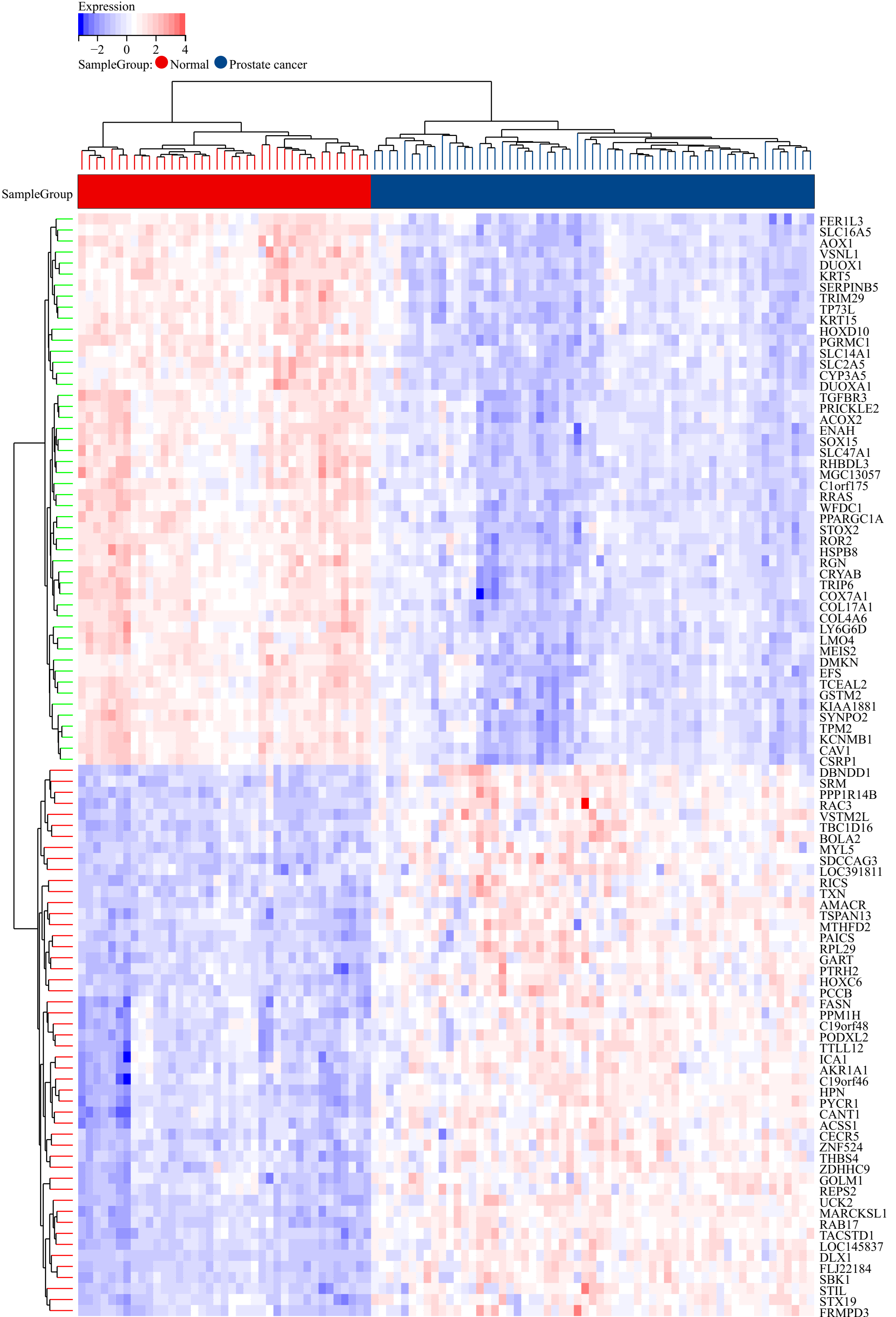

Supplement: Supplementary file 1 [file DataSheet_1.zip › raw data 1/Fig 1/Fig 1b.pdf]

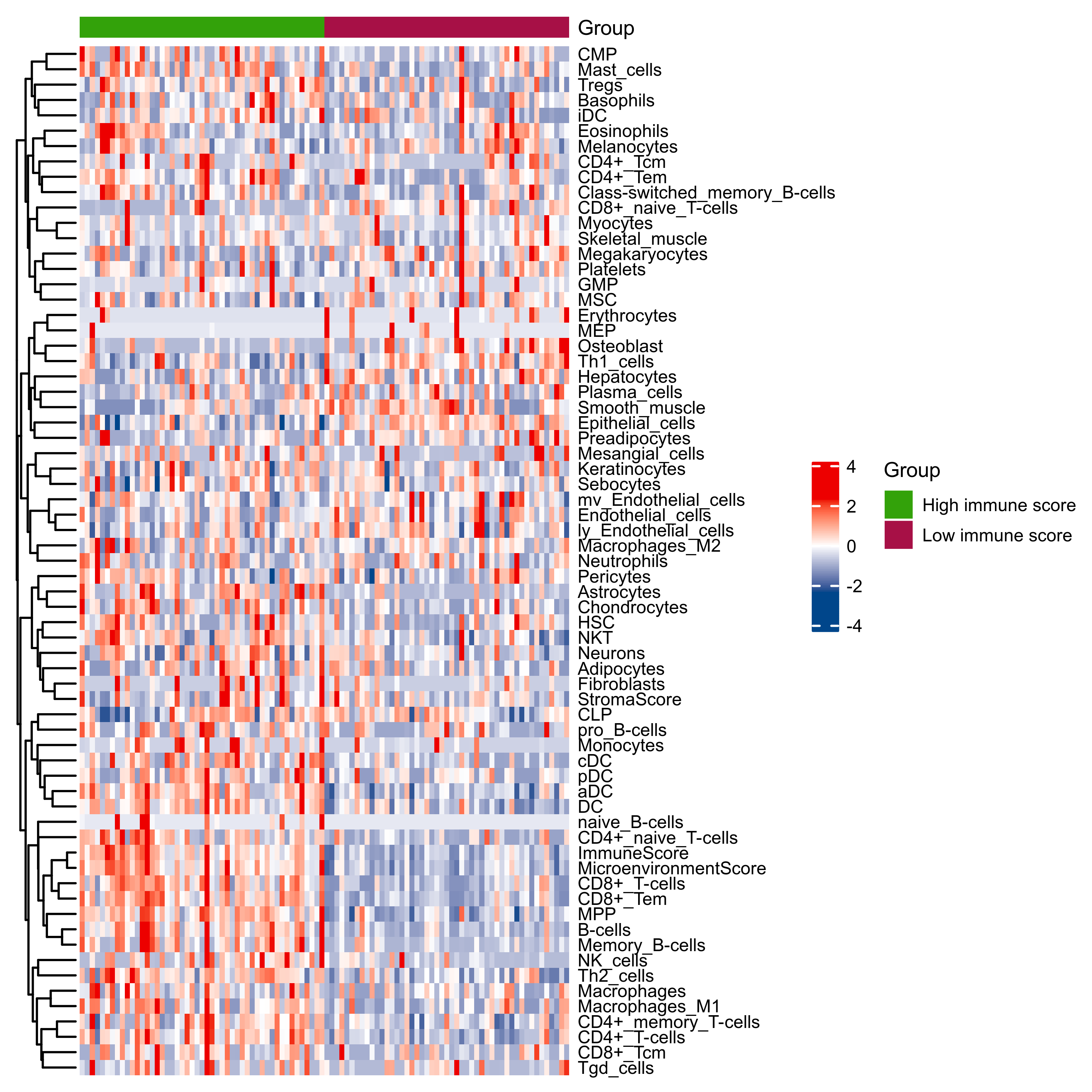

Supplement: Supplementary file 1 [file DataSheet_1.zip › raw data 1/Fig 2/Fig 2a.tiff]

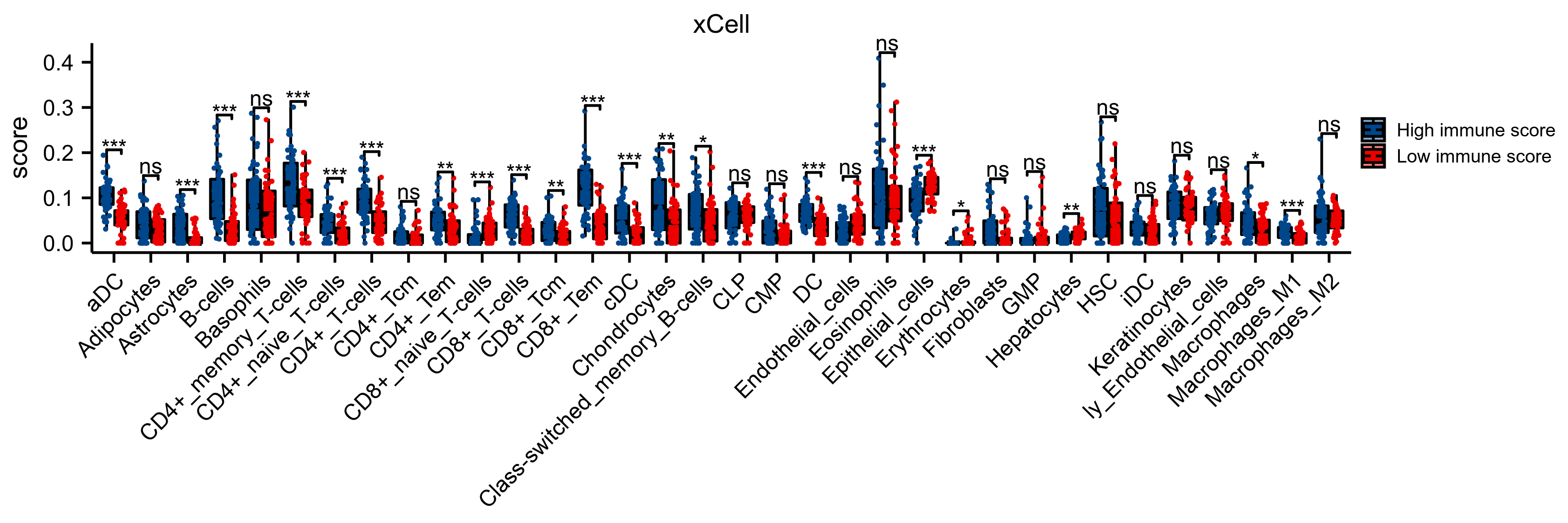

Supplement: Supplementary file 1 [file DataSheet_1.zip › raw data 1/Fig 2/Fig 2b.tiff]

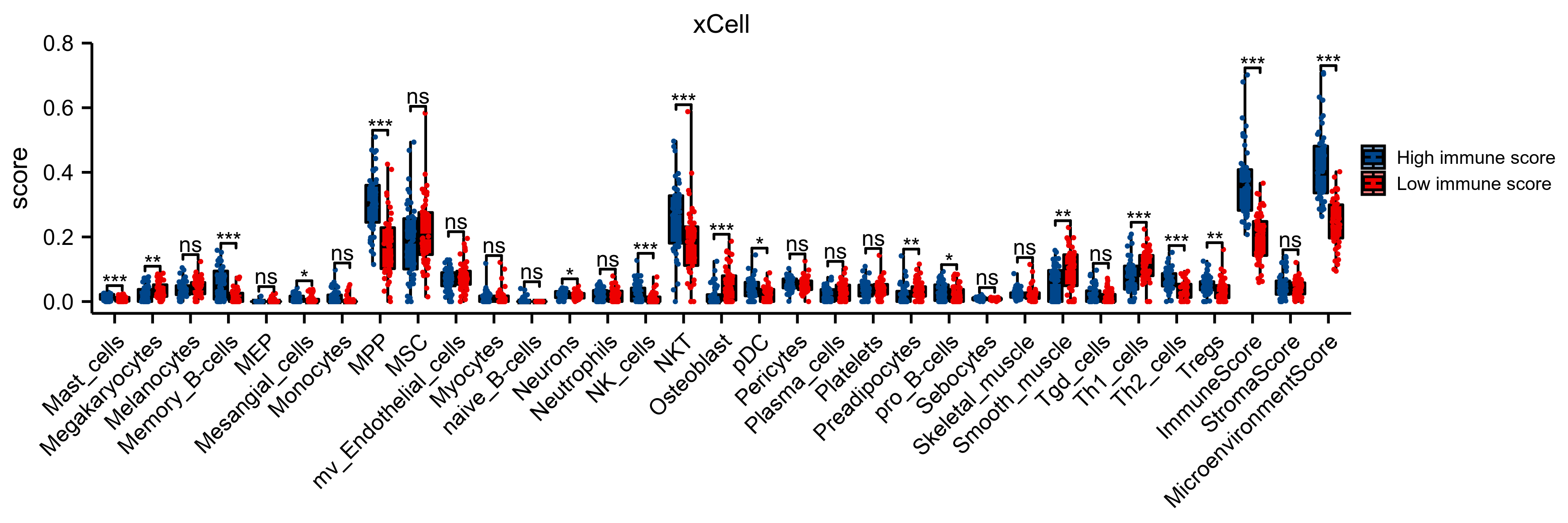

Supplement: Supplementary file 1 [file DataSheet_1.zip › raw data 1/Fig 2/Fig 2c.tiff]

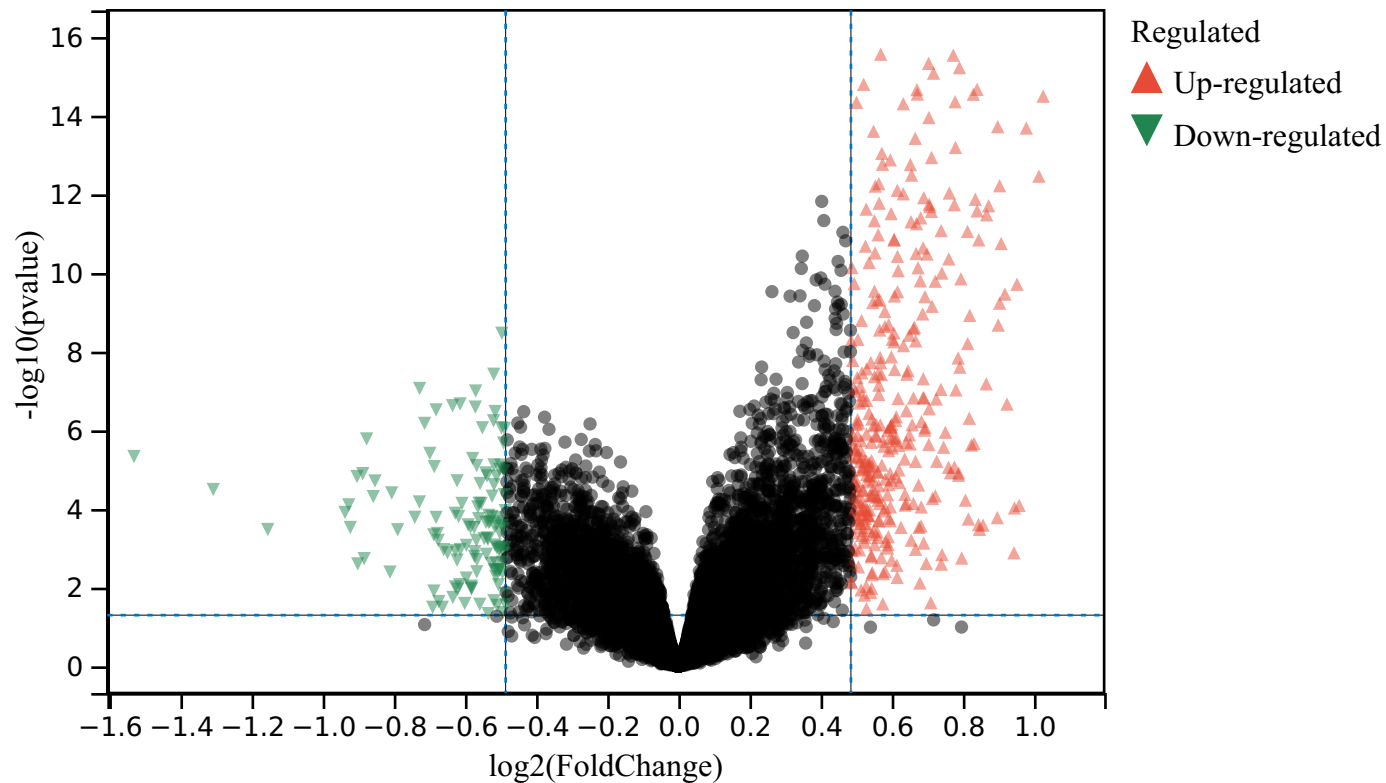

Supplement: Supplementary file 1 [file DataSheet_1.zip › raw data 1/Fig 3/Fig 3a.pdf]

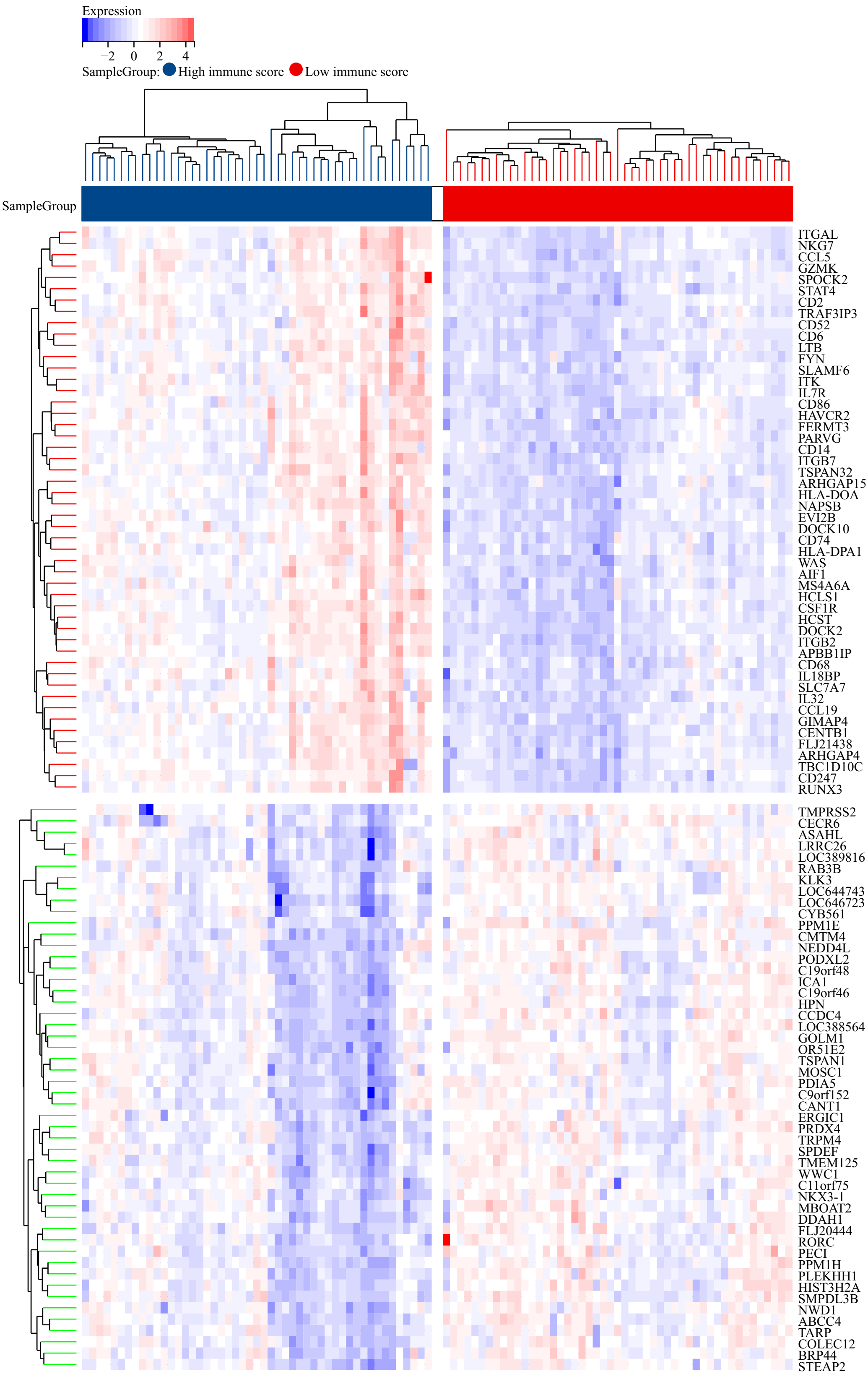

Supplement: Supplementary file 1 [file DataSheet_1.zip › raw data 1/Fig 3/Fig 3b.pdf]

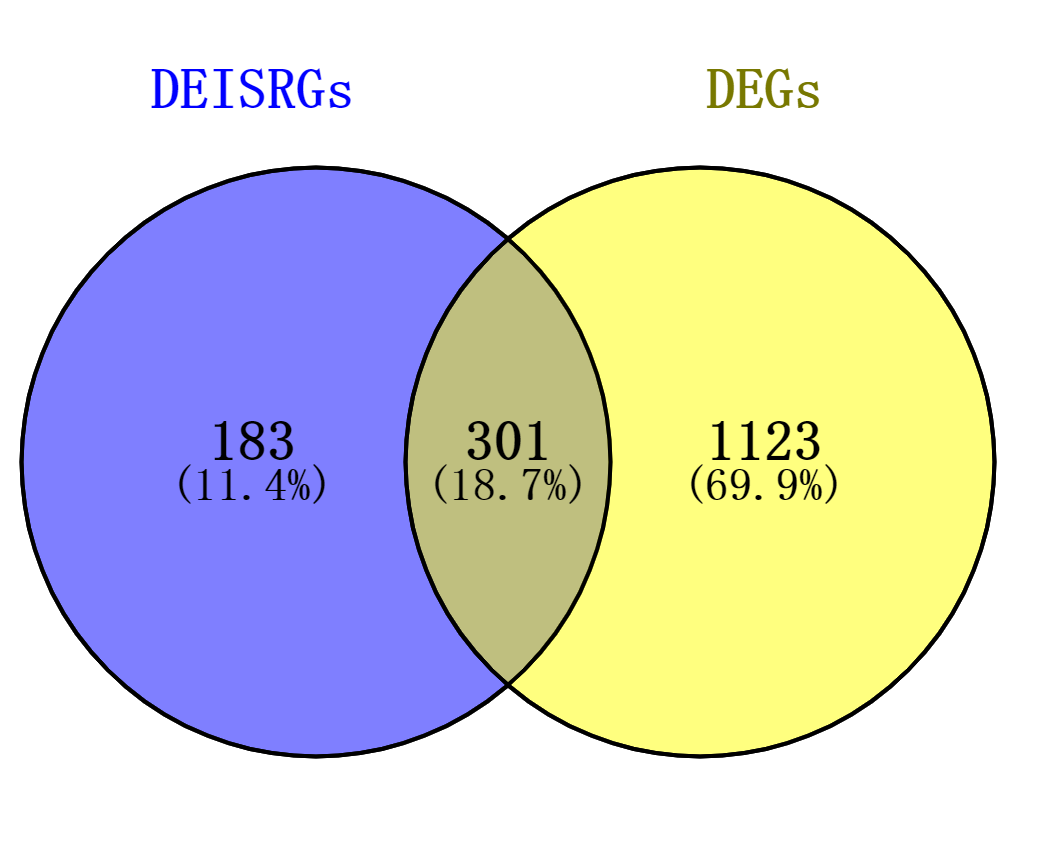

Supplement: Supplementary file 1 [file DataSheet_1.zip › raw data 1/Fig 4/Fig 4a.png]

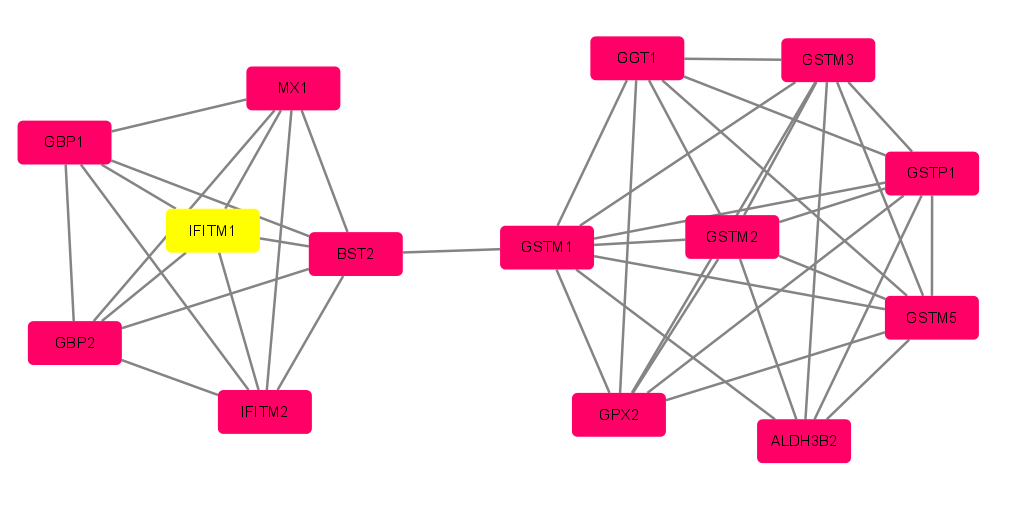

Supplement: Supplementary file 1 [file DataSheet_1.zip › raw data 1/Fig 4/Fig 4b.png]

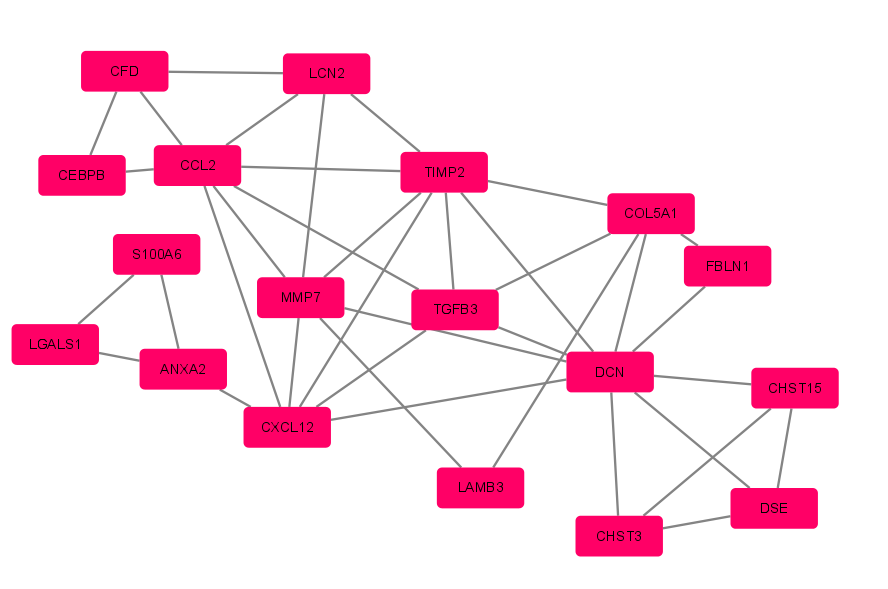

Supplement: Supplementary file 1 [file DataSheet_1.zip › raw data 1/Fig 4/Fig 4c.png]

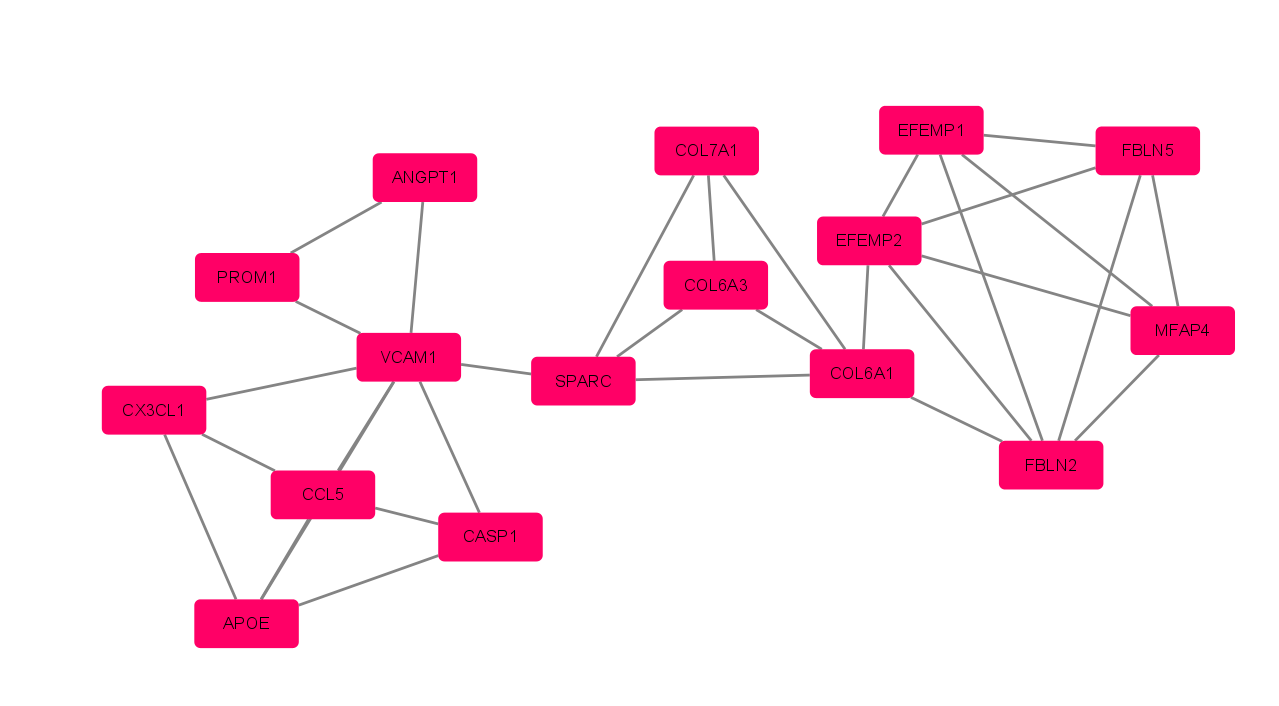

Supplement: Supplementary file 1 [file DataSheet_1.zip › raw data 1/Fig 4/Fig 4d.png]

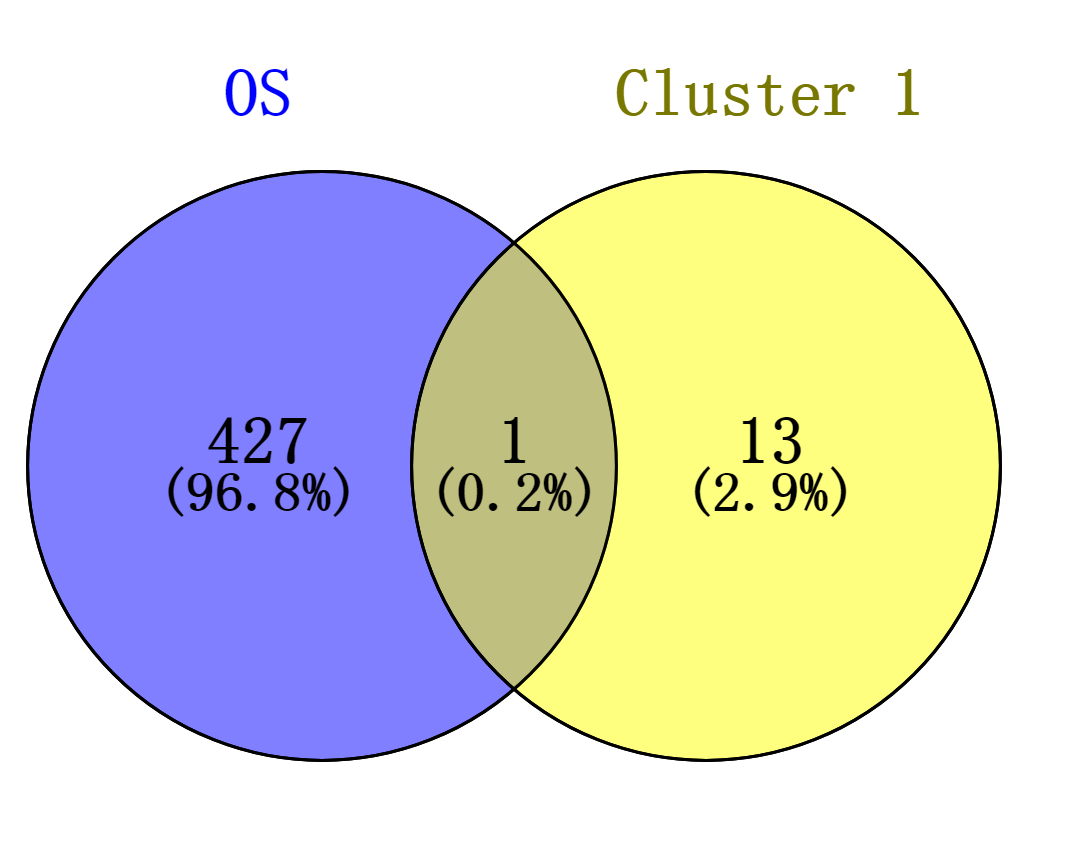

Supplement: Supplementary file 1 [file DataSheet_1.zip › raw data 1/Fig 4/Fig 4e.png]

IFITM1 Expression Level (log2 TPM)

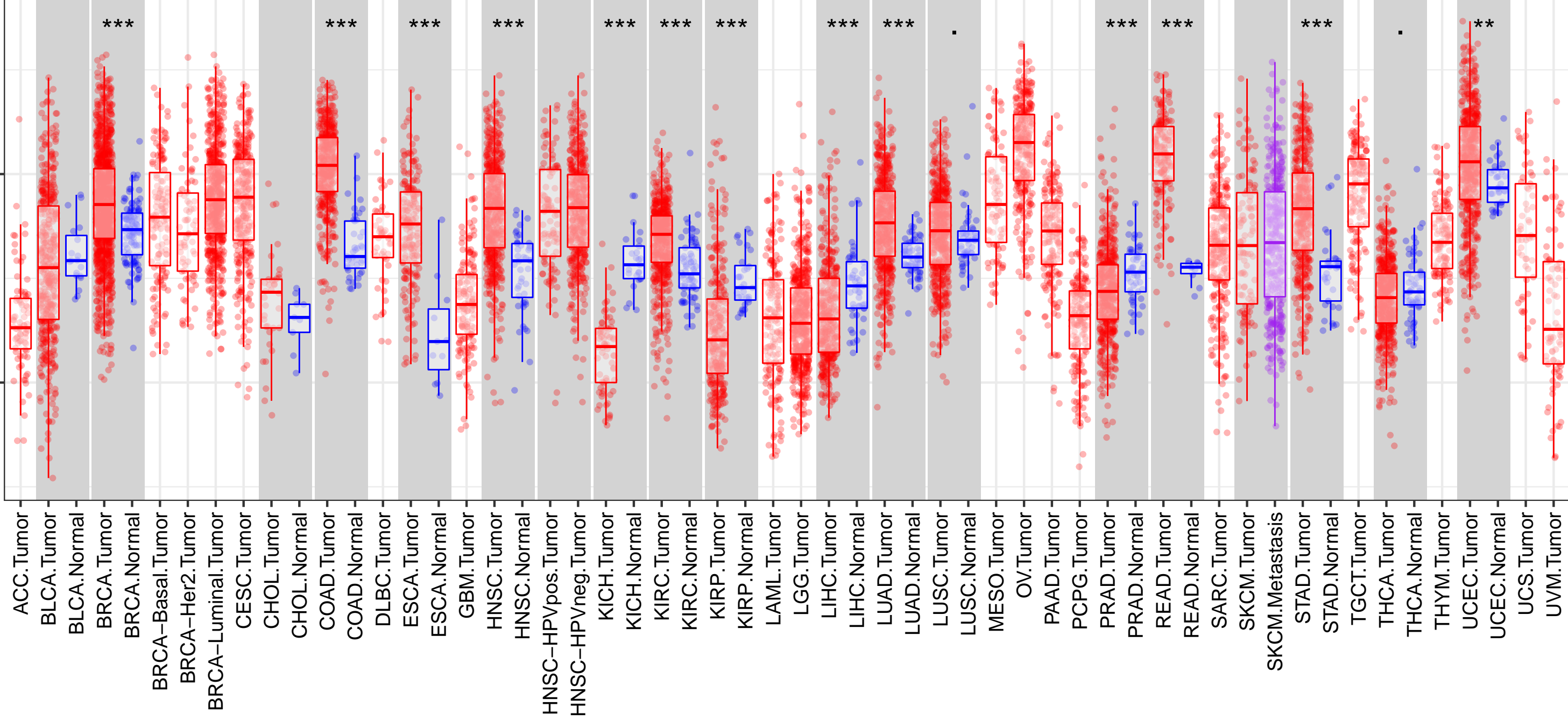

Supplement: Supplementary file 1 [file DataSheet_1.zip › raw data 1/Fig 5/Fig 5a.pdf]

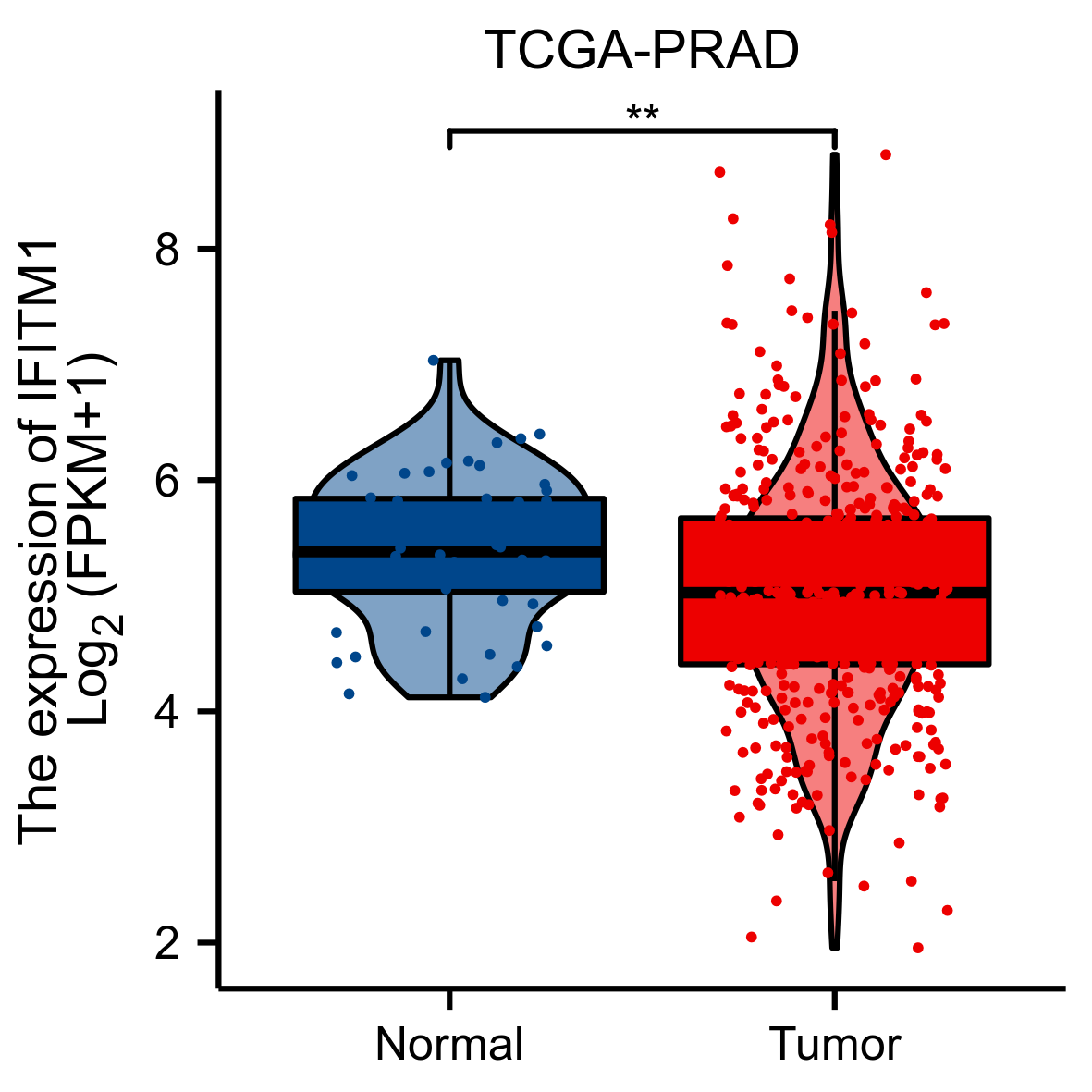

Supplement: Supplementary file 1 [file DataSheet_1.zip › raw data 1/Fig 5/Fig 5b.tiff]

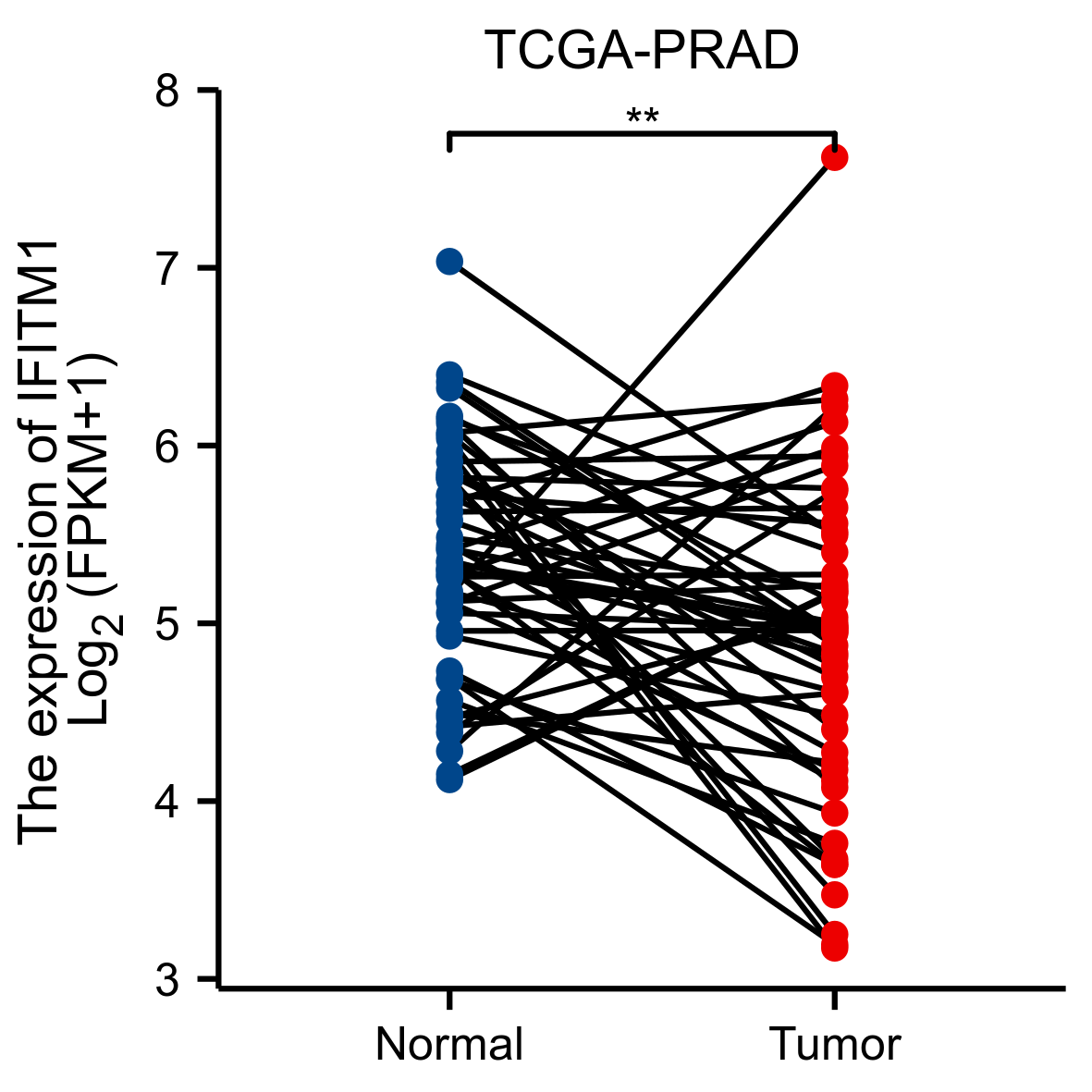

Supplement: Supplementary file 1 [file DataSheet_1.zip › raw data 1/Fig 5/Fig 5c.tiff]

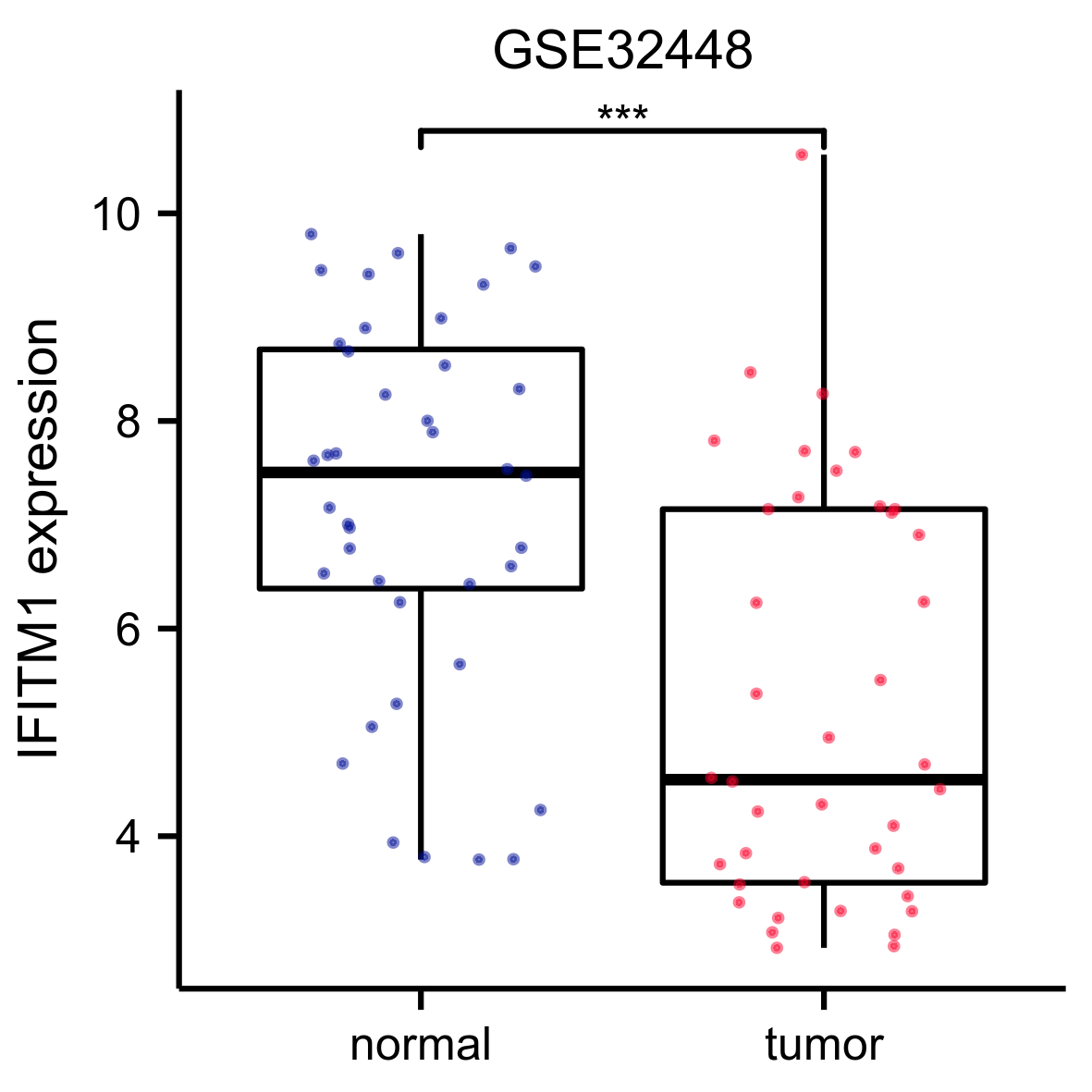

Supplement: Supplementary file 1 [file DataSheet_1.zip › raw data 1/Fig 5/Fig 5d.tiff]

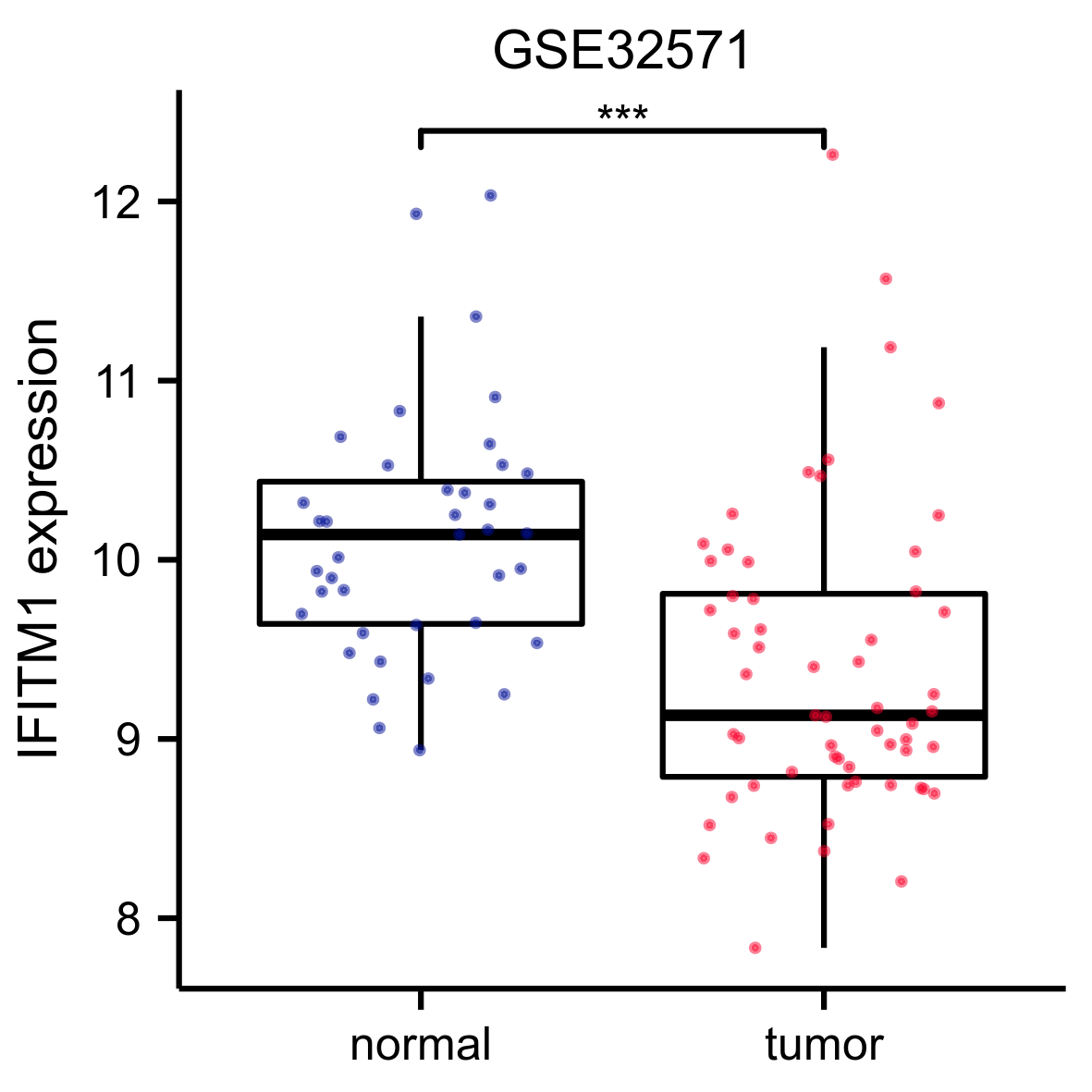

Supplement: Supplementary file 1 [file DataSheet_1.zip › raw data 1/Fig 5/Fig 5e.tiff]

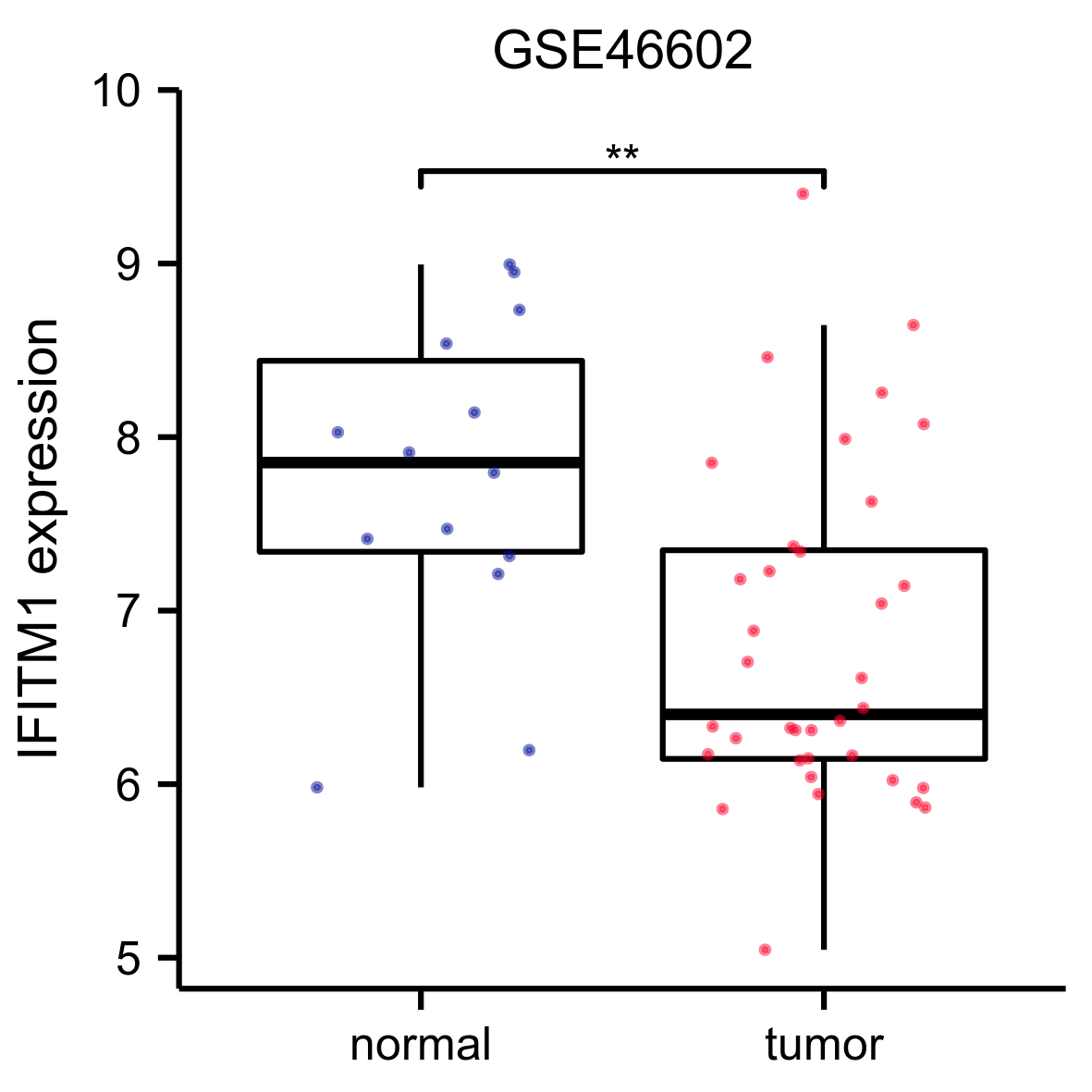

Supplement: Supplementary file 1 [file DataSheet_1.zip › raw data 1/Fig 5/Fig 5f.tiff]

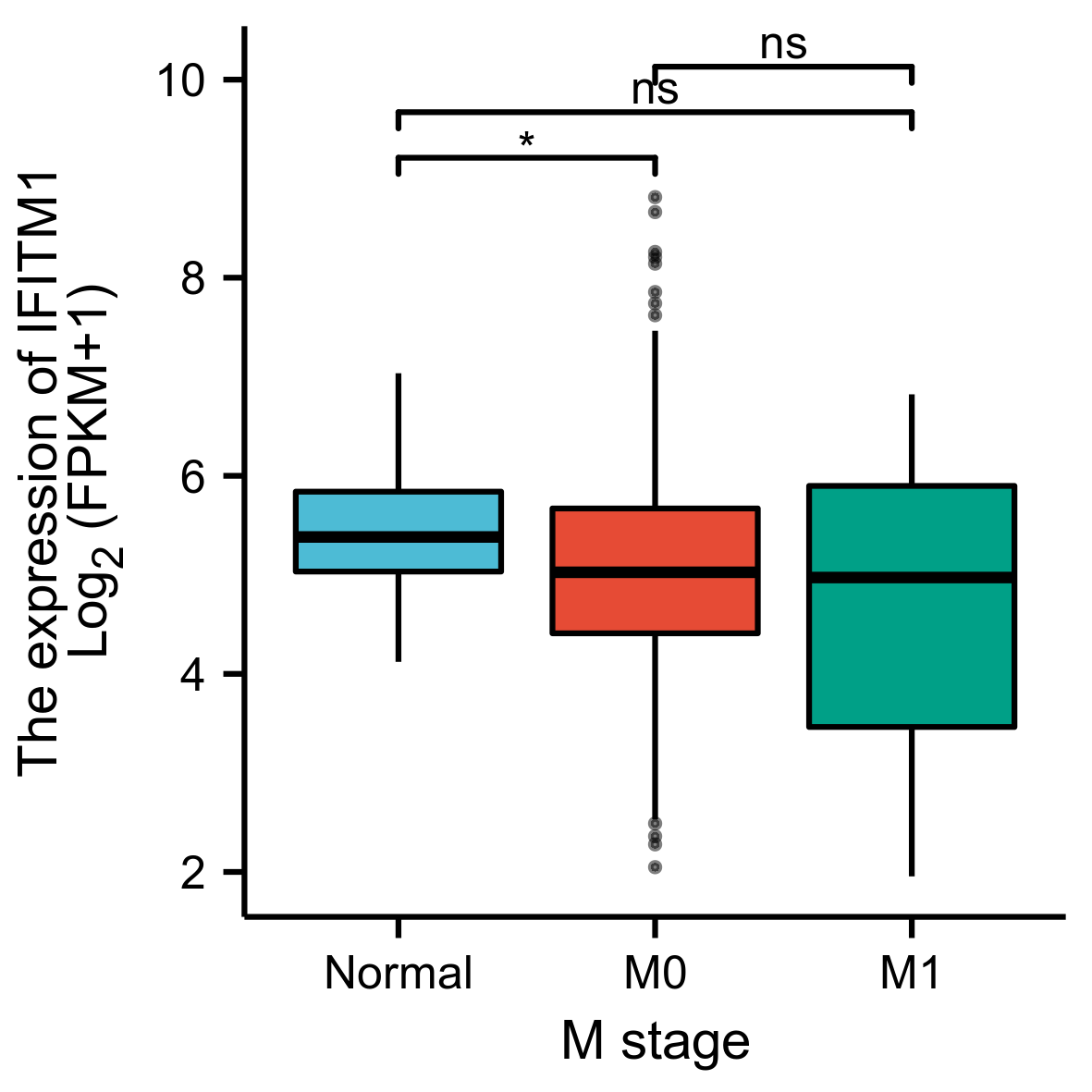

Supplement: Supplementary file 1 [file DataSheet_1.zip › raw data 1/Fig 6/fig 6a.tiff]

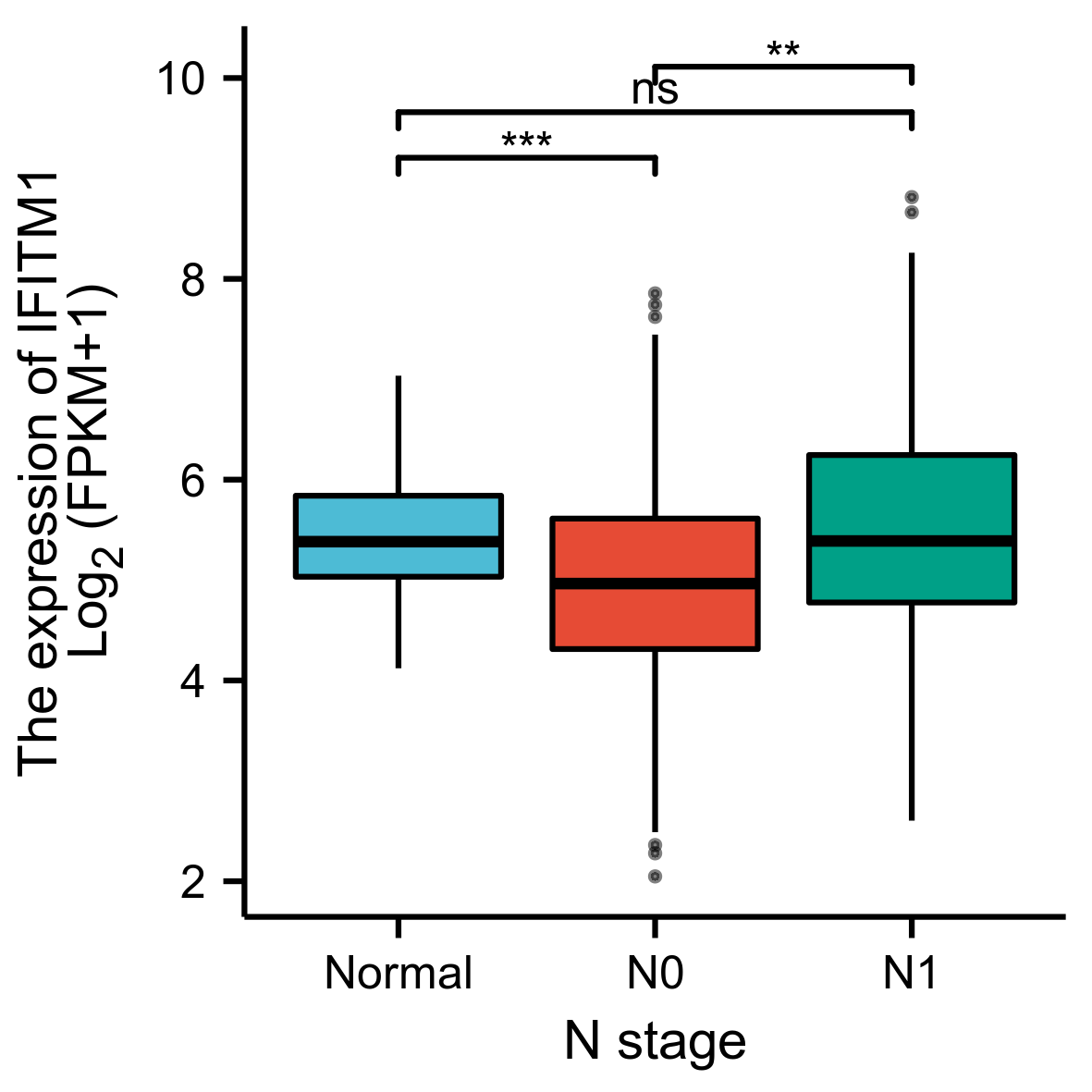

Supplement: Supplementary file 1 [file DataSheet_1.zip › raw data 1/Fig 6/fig 6b.tiff]

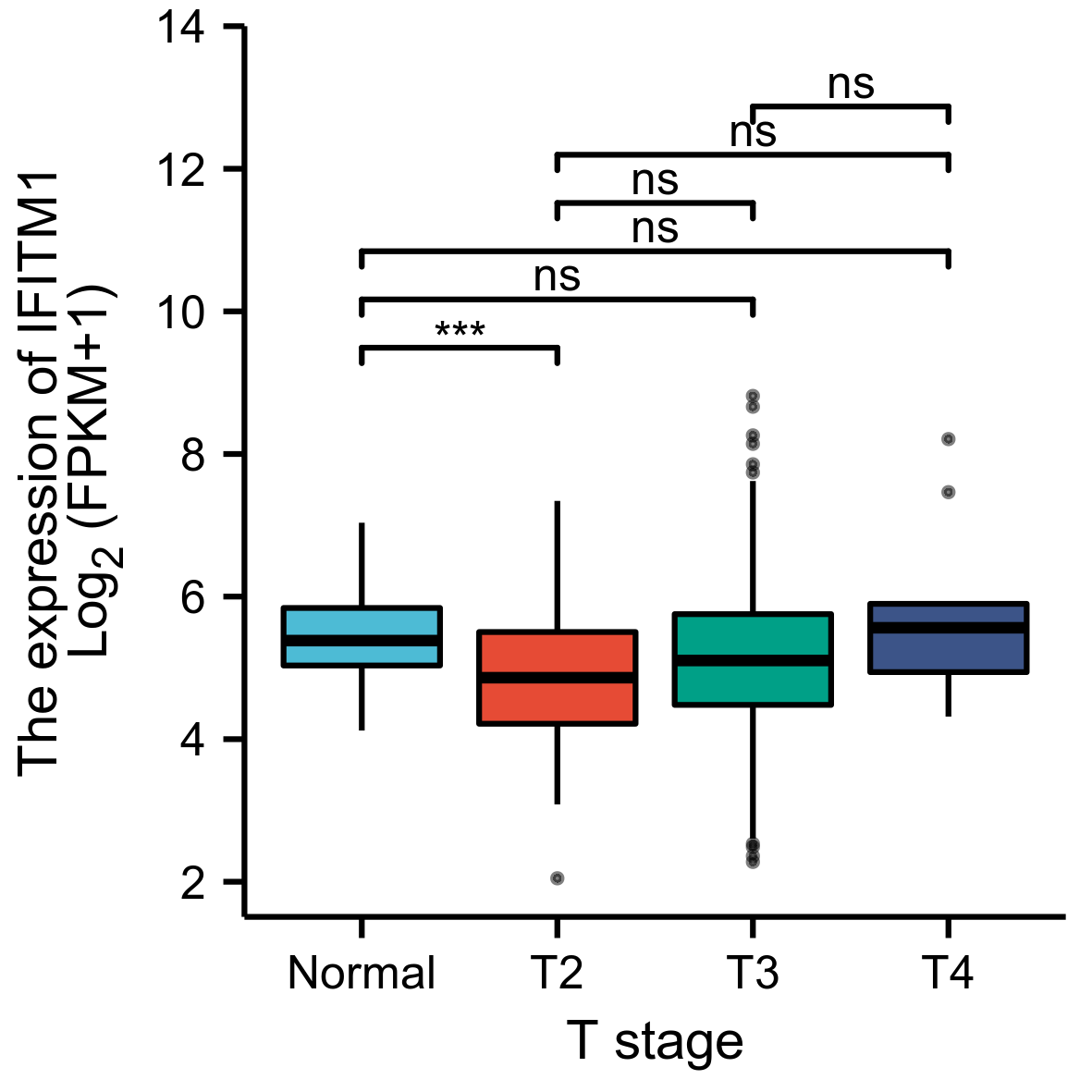

Supplement: Supplementary file 1 [file DataSheet_1.zip › raw data 1/Fig 6/fig 6c.tiff]

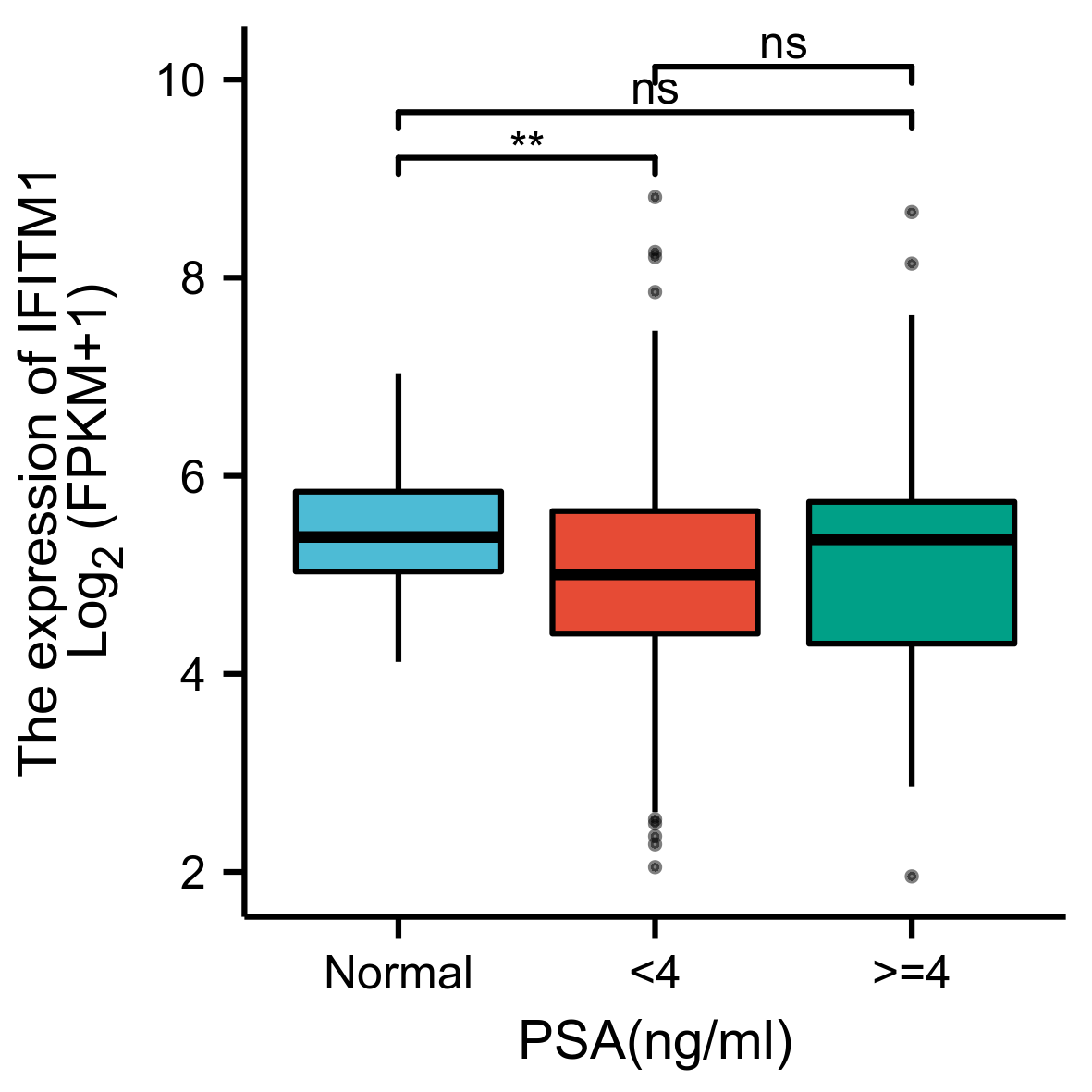

Supplement: Supplementary file 1 [file DataSheet_1.zip › raw data 1/Fig 6/fig 6d.tiff]

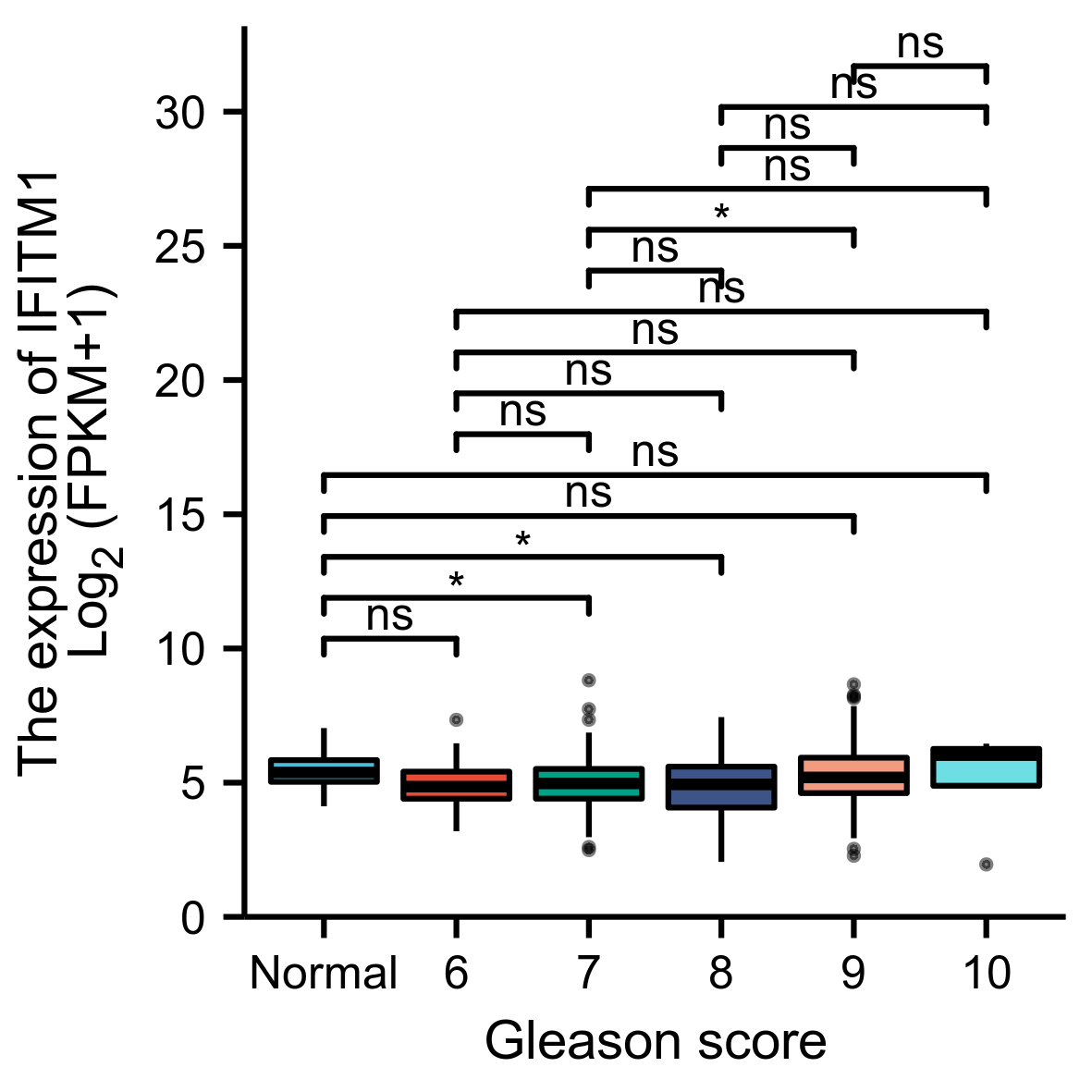

Supplement: Supplementary file 1 [file DataSheet_1.zip › raw data 1/Fig 6/fig 6e.tiff]

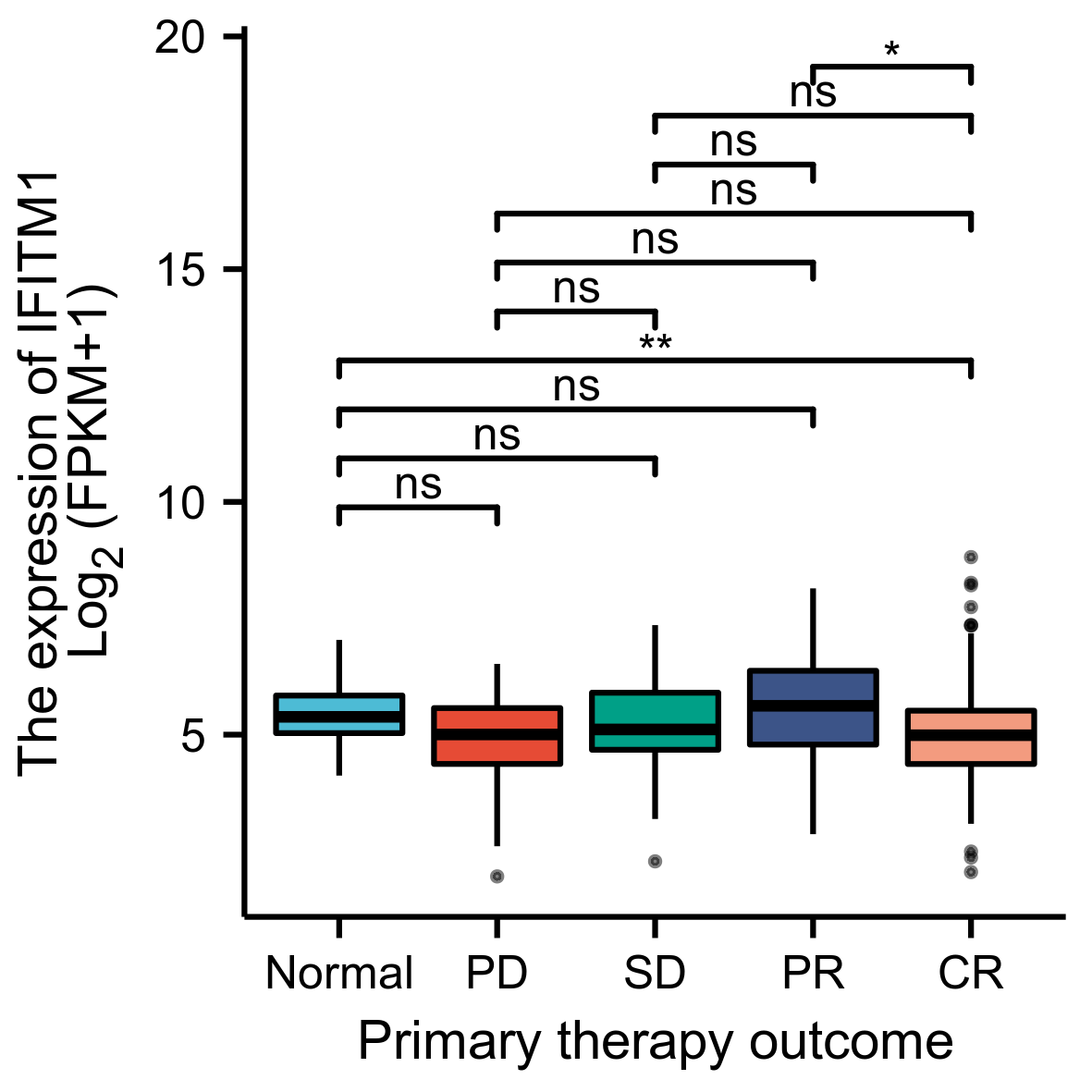

Supplement: Supplementary file 1 [file DataSheet_1.zip › raw data 1/Fig 6/fig 6f.tiff]

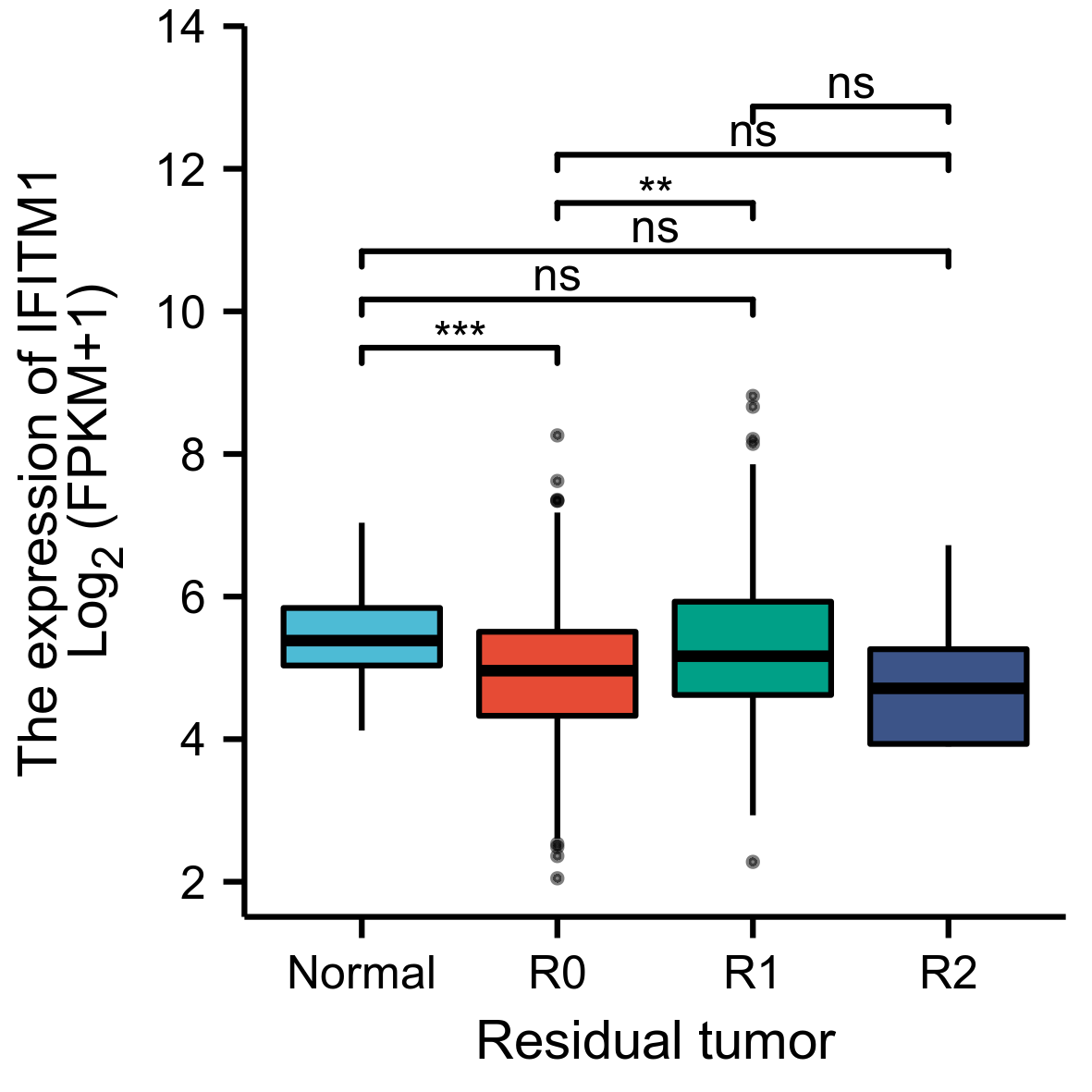

Supplement: Supplementary file 1 [file DataSheet_1.zip › raw data 1/Fig 6/fig 6g.tiff]

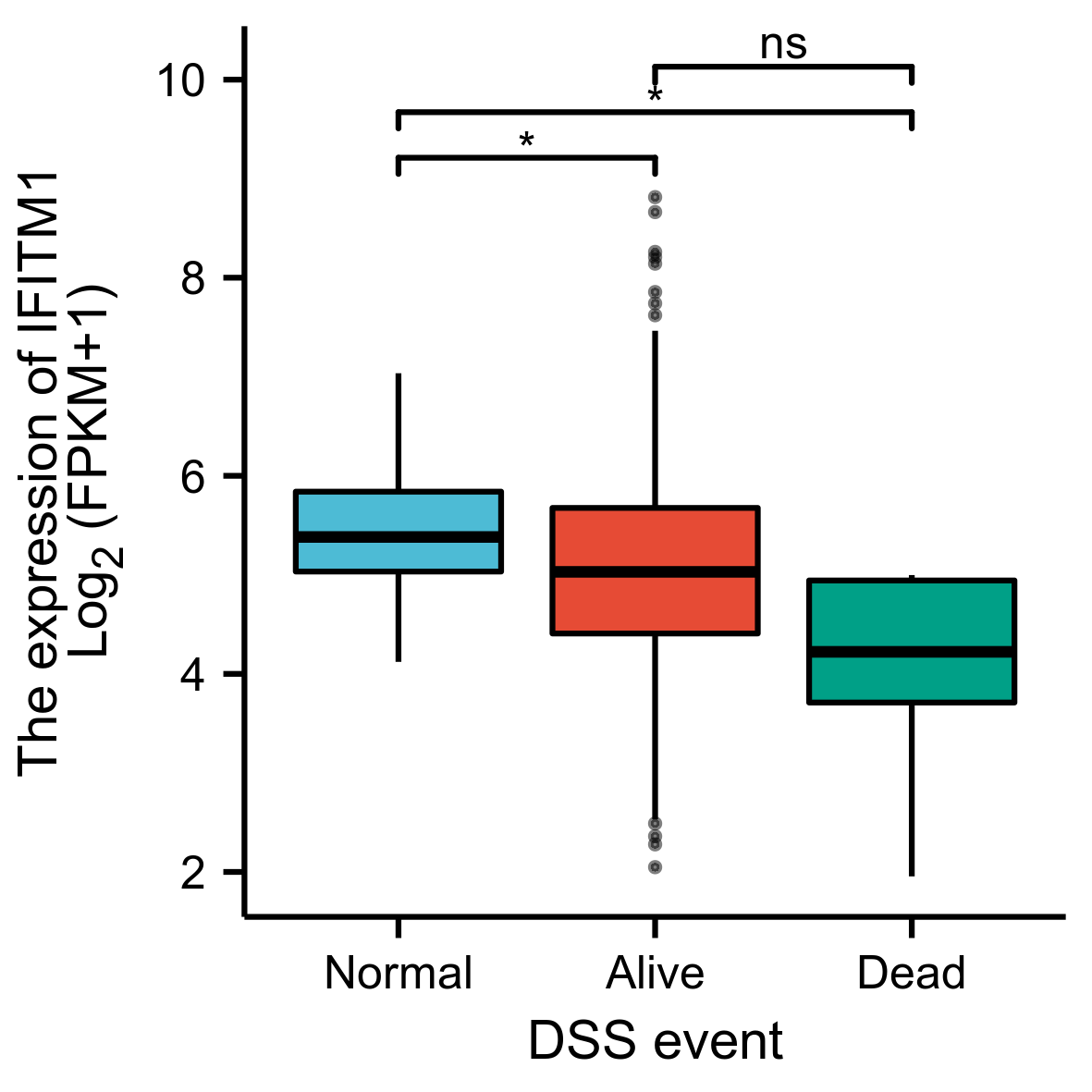

Supplement: Supplementary file 1 [file DataSheet_1.zip › raw data 1/Fig 6/fig 6h.tiff]

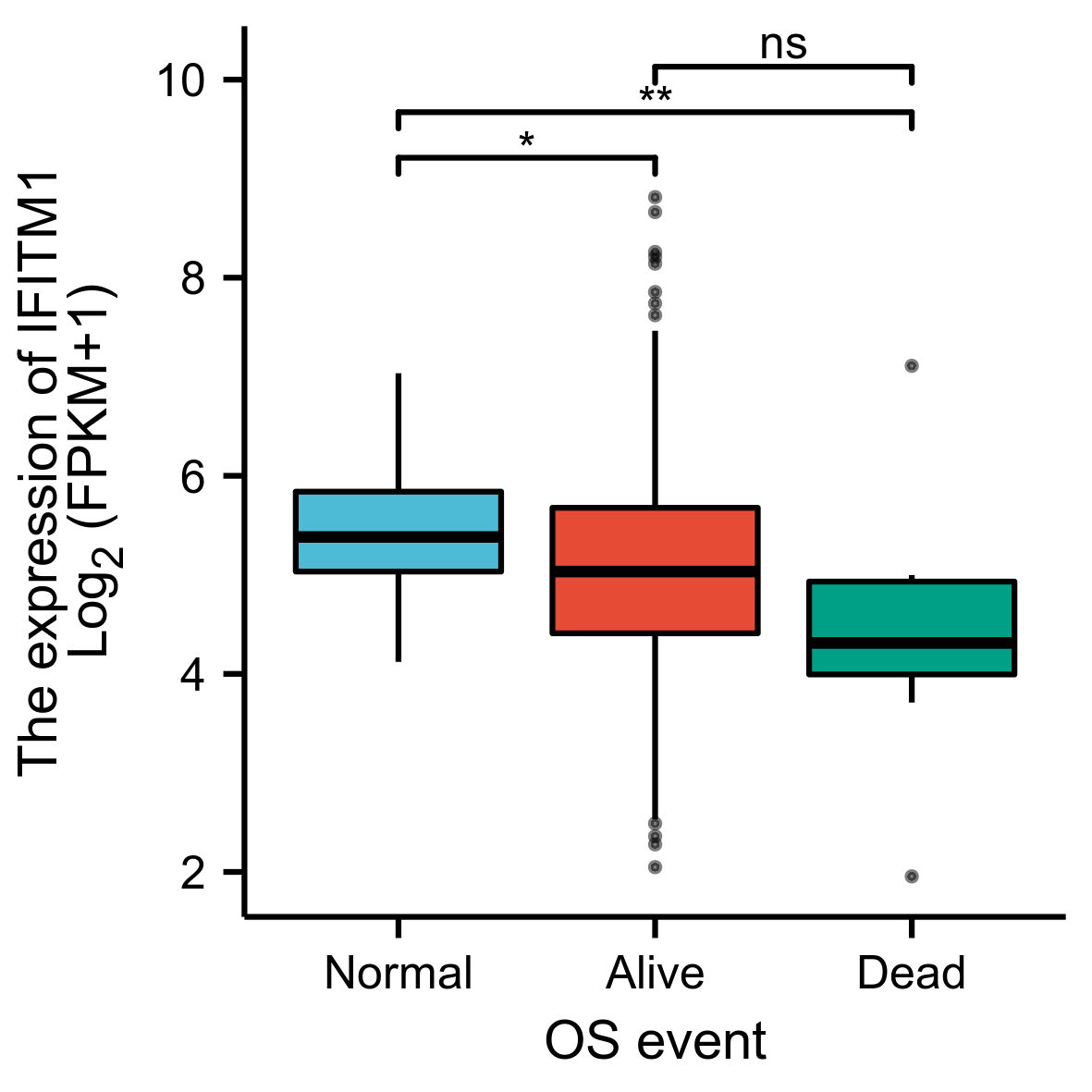

Supplement: Supplementary file 1 [file DataSheet_1.zip › raw data 1/Fig 6/fig 6i.tiff]

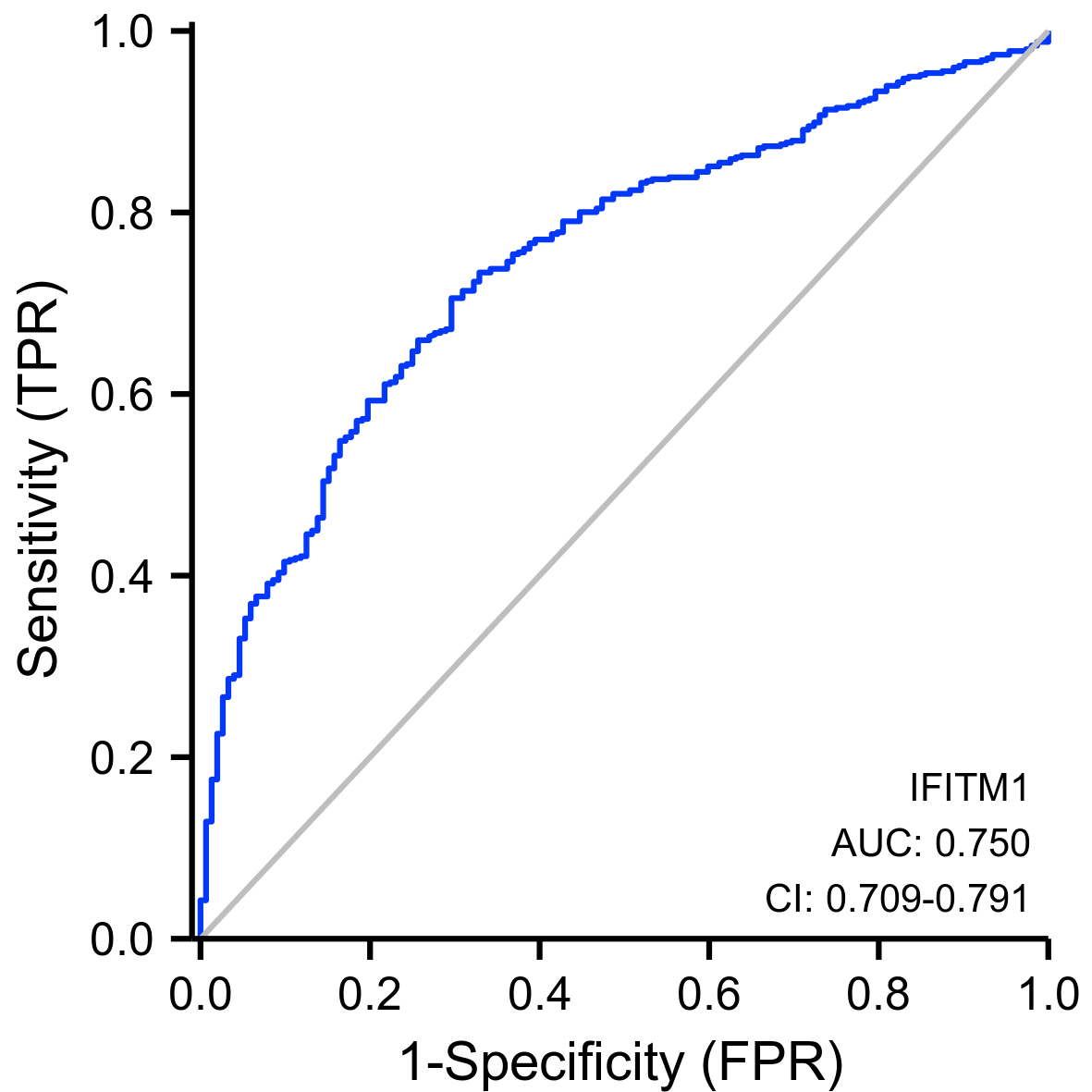

Supplement: Supplementary file 1 [file DataSheet_1.zip › raw data 1/Fig 7/fig 7.tiff]

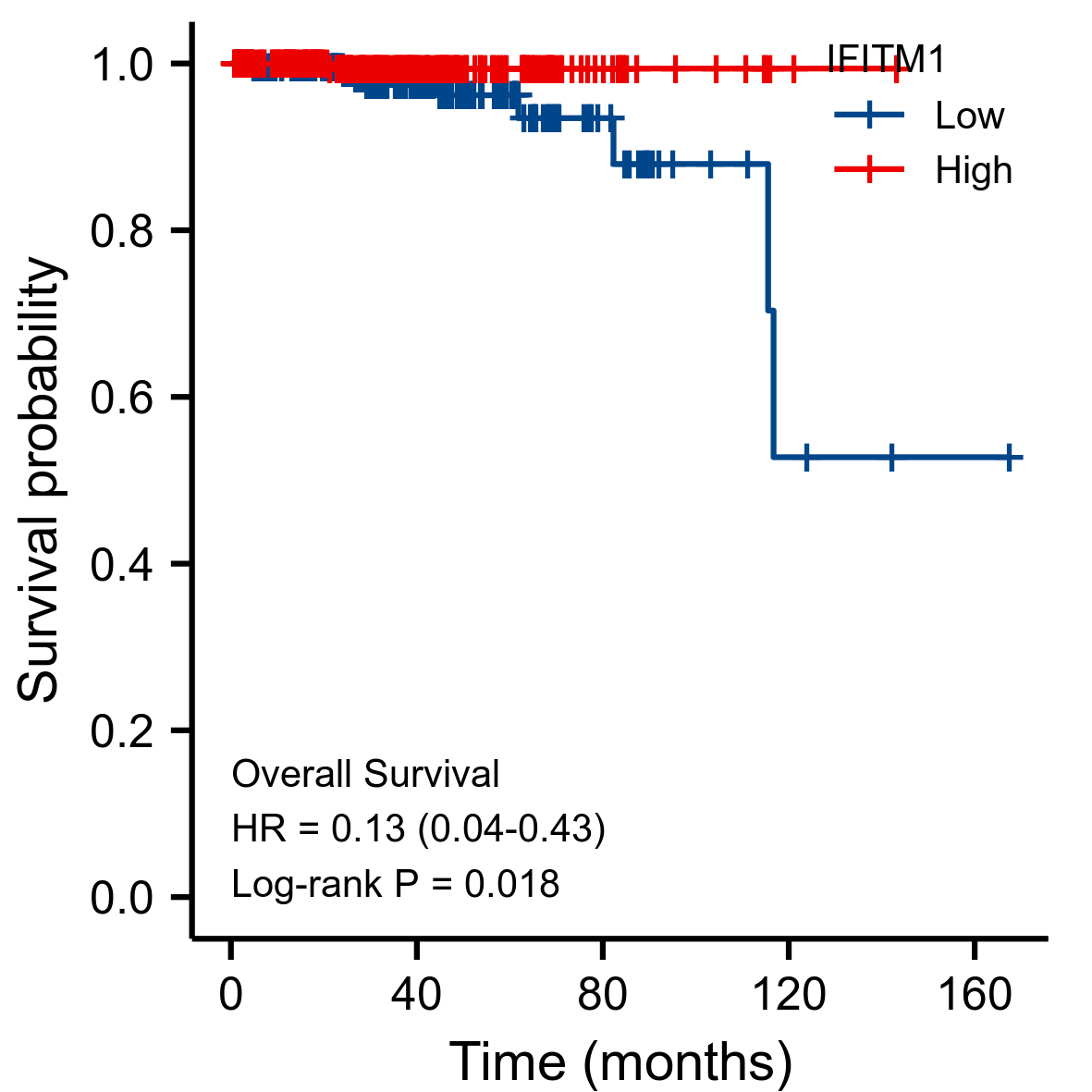

Supplement: Supplementary file 2 [file DataSheet_2.zip › raw data 2/Fig 8/fig 8a.tiff]

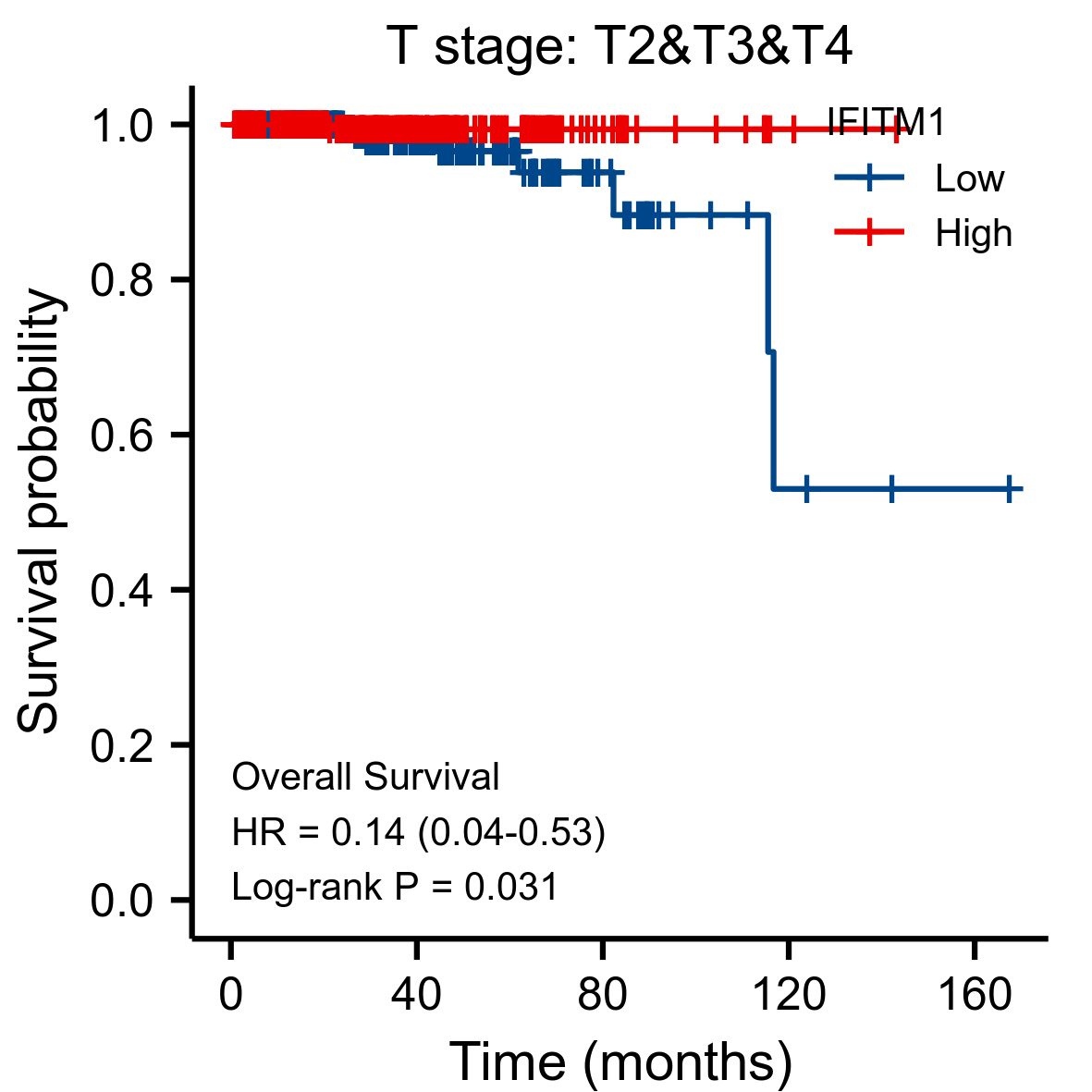

Supplement: Supplementary file 2 [file DataSheet_2.zip › raw data 2/Fig 8/fig 8b.tiff]

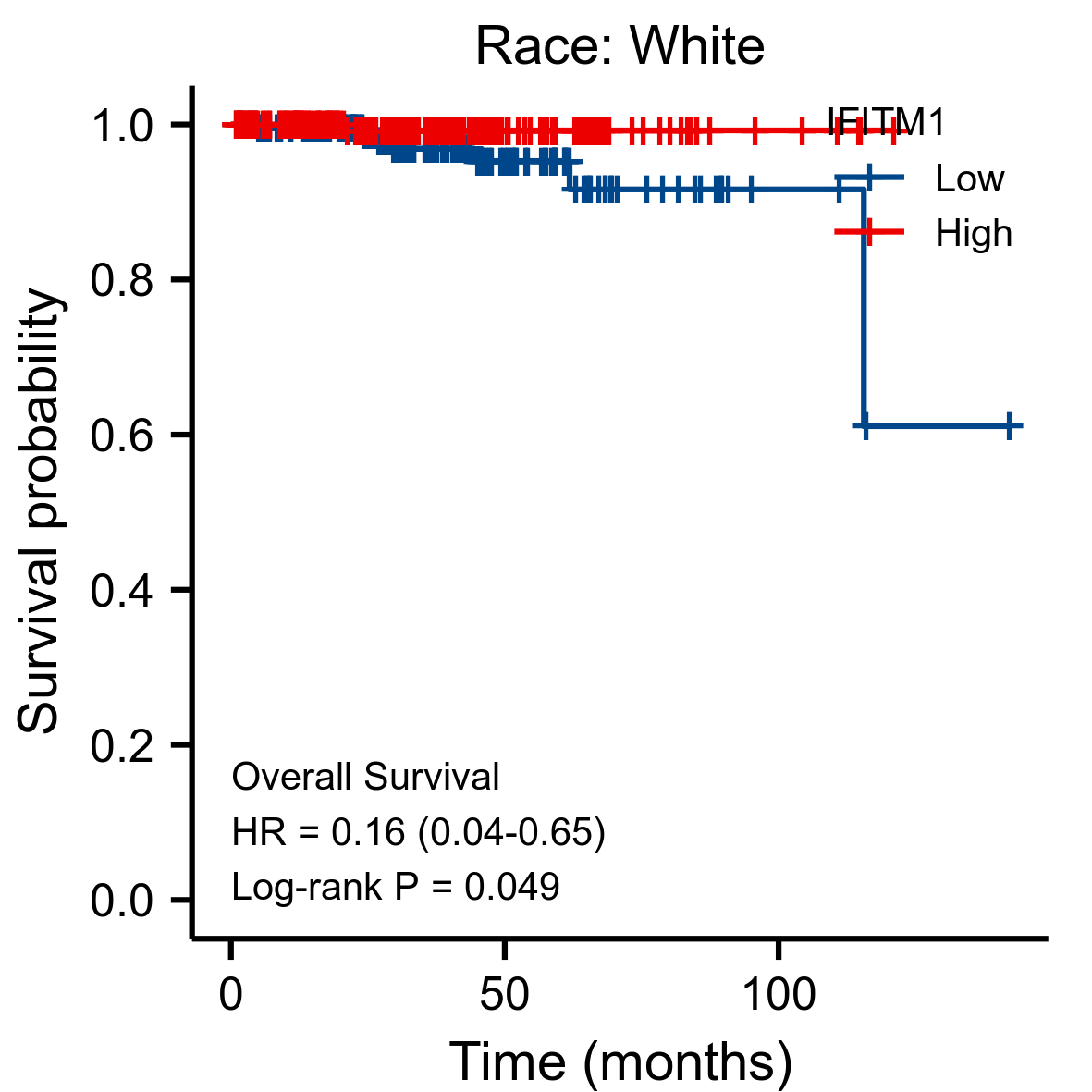

Supplement: Supplementary file 2 [file DataSheet_2.zip › raw data 2/Fig 8/fig 8c.tiff]

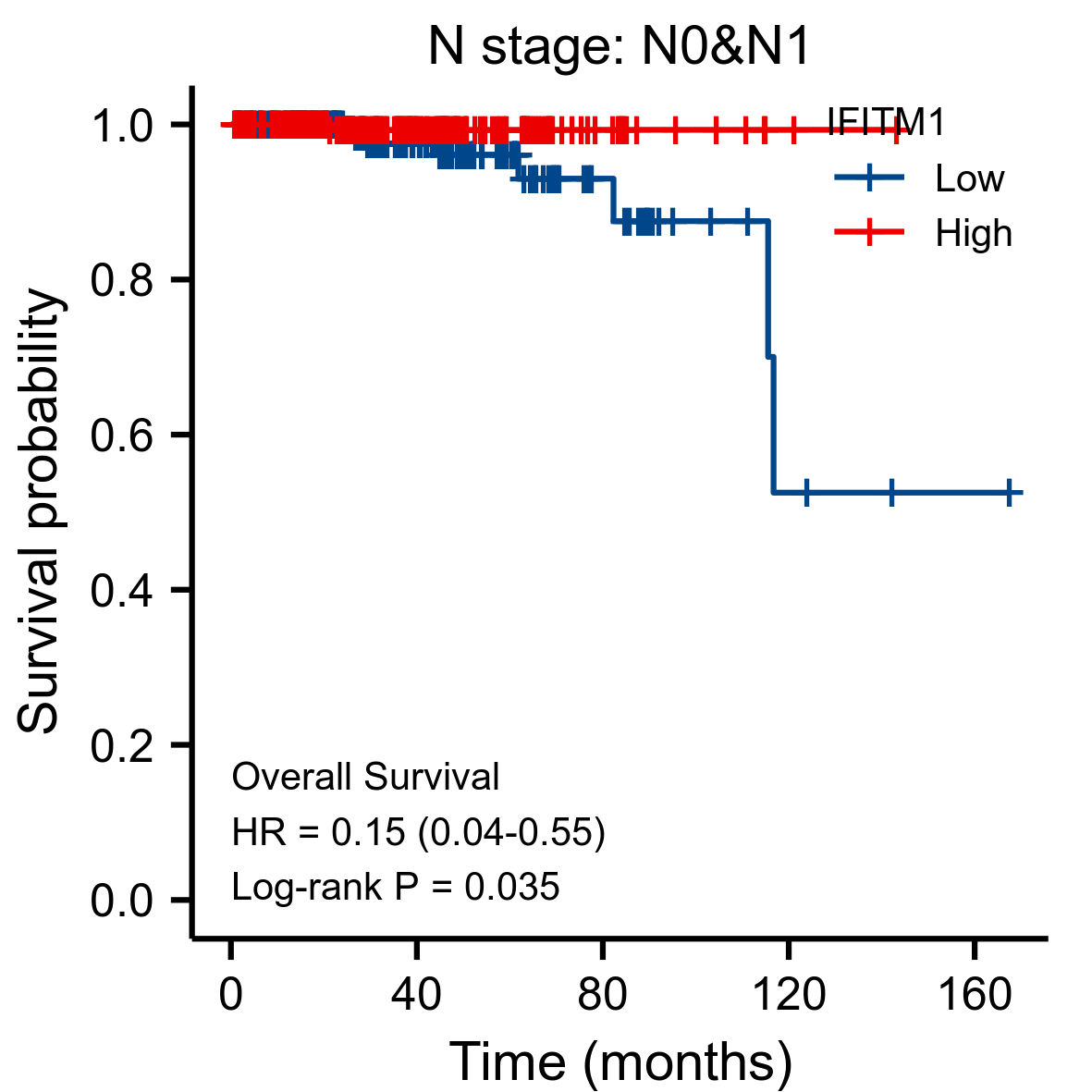

Supplement: Supplementary file 2 [file DataSheet_2.zip › raw data 2/Fig 8/fig 8d.tiff]

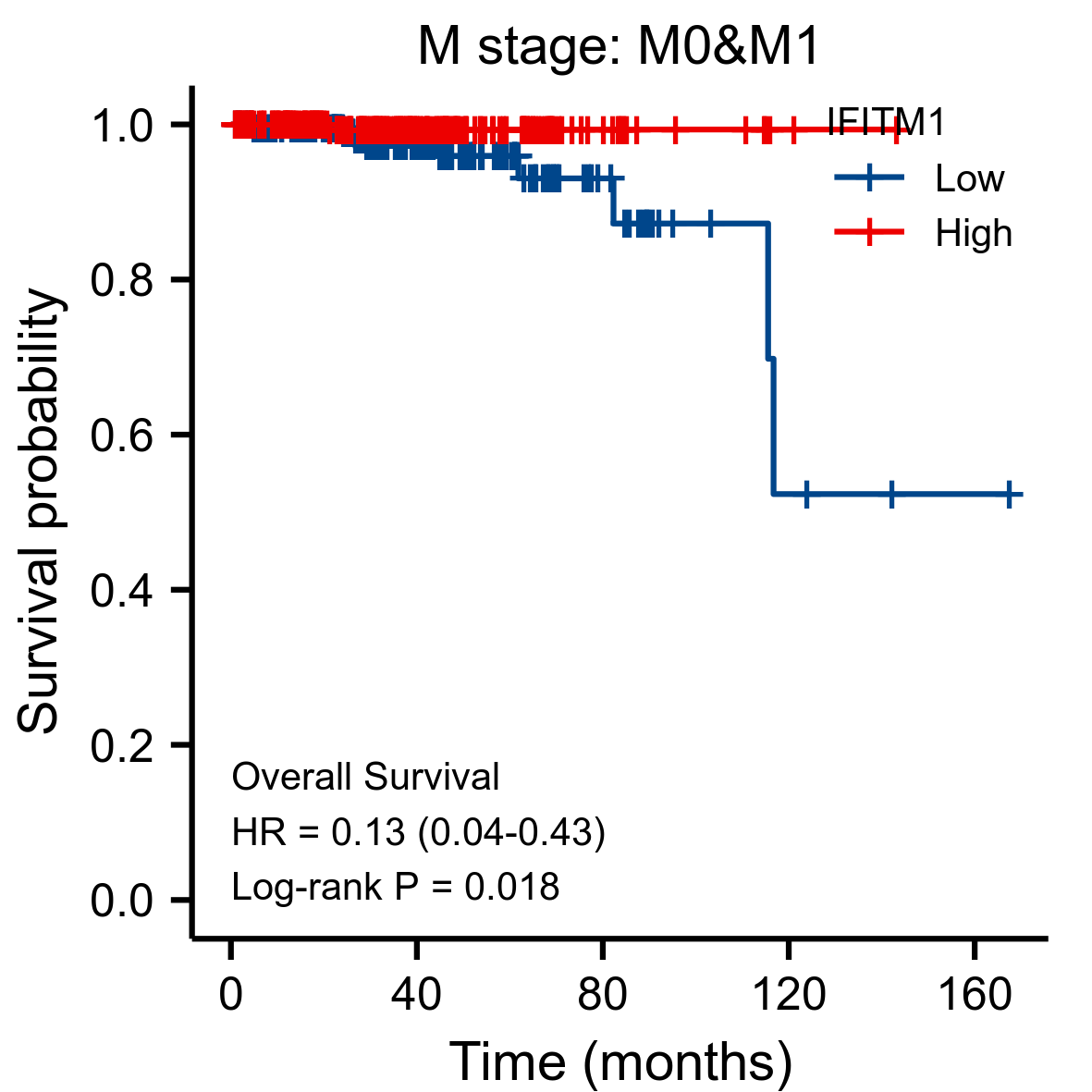

Supplement: Supplementary file 2 [file DataSheet_2.zip › raw data 2/Fig 8/fig 8e.tiff]

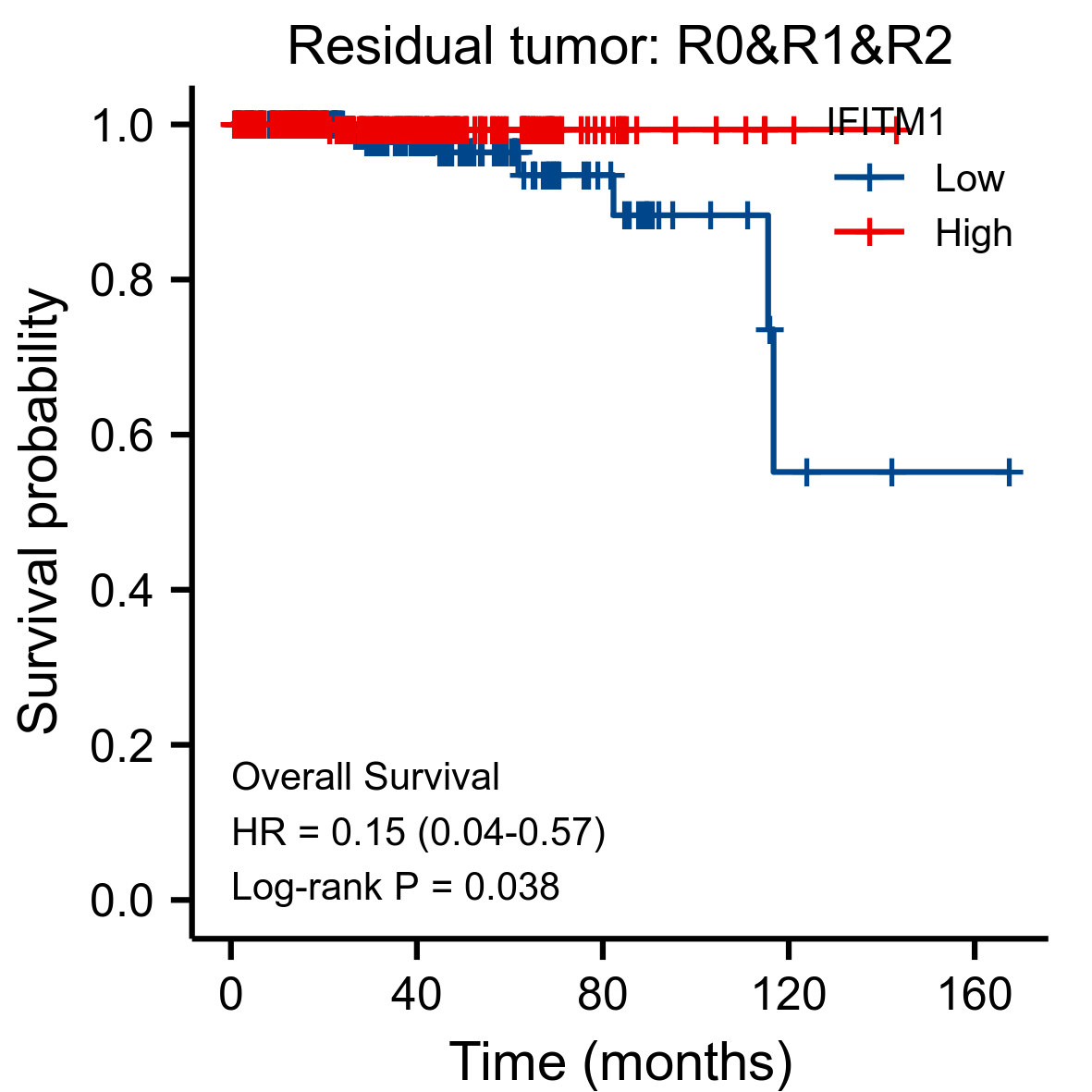

Supplement: Supplementary file 2 [file DataSheet_2.zip › raw data 2/Fig 8/fig 8f.tiff]

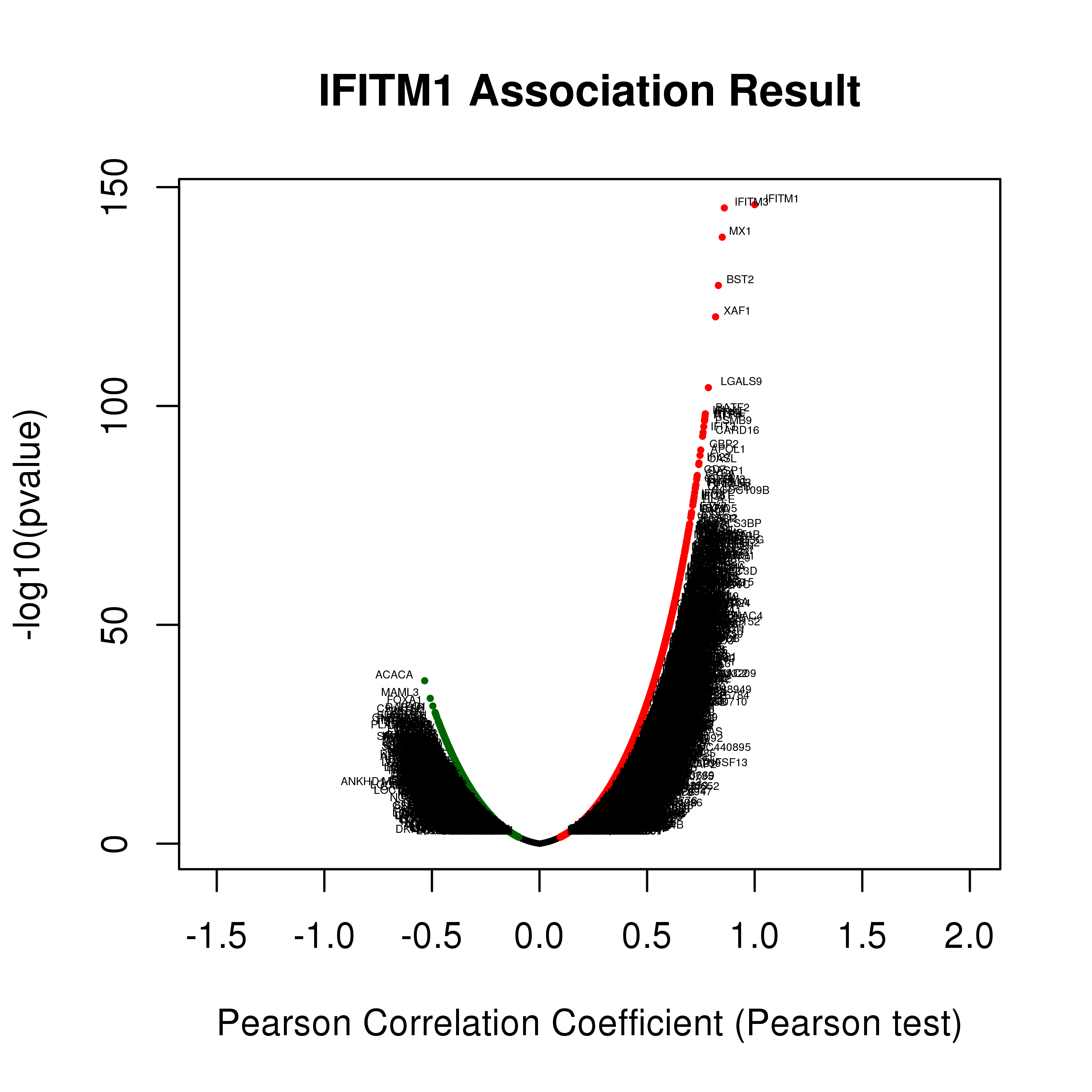

Supplement: Supplementary file 2 [file DataSheet_2.zip › raw data 2/Fig 9/fig 9a.png]

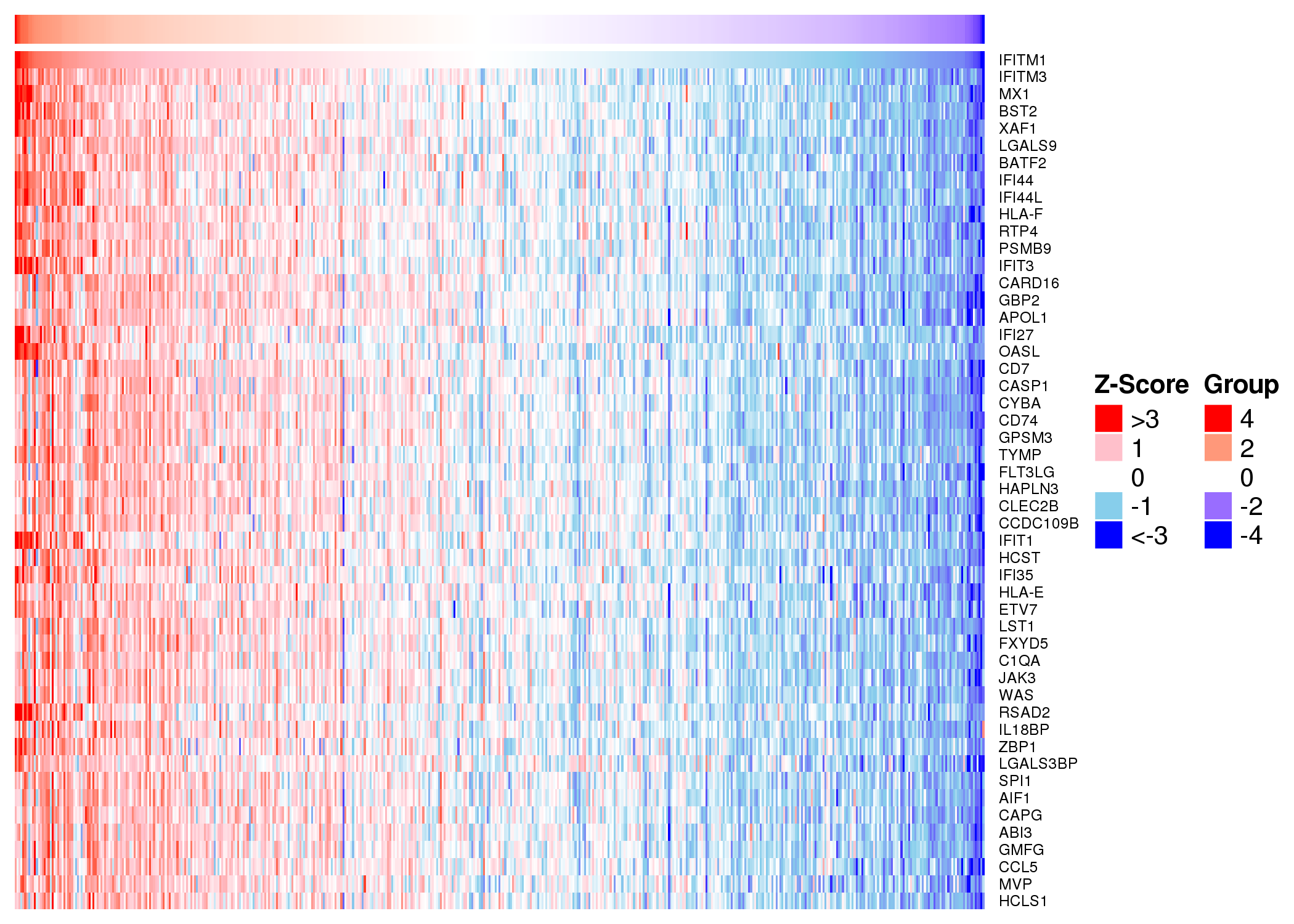

Supplement: Supplementary file 2 [file DataSheet_2.zip › raw data 2/Fig 9/fig 9b.png]

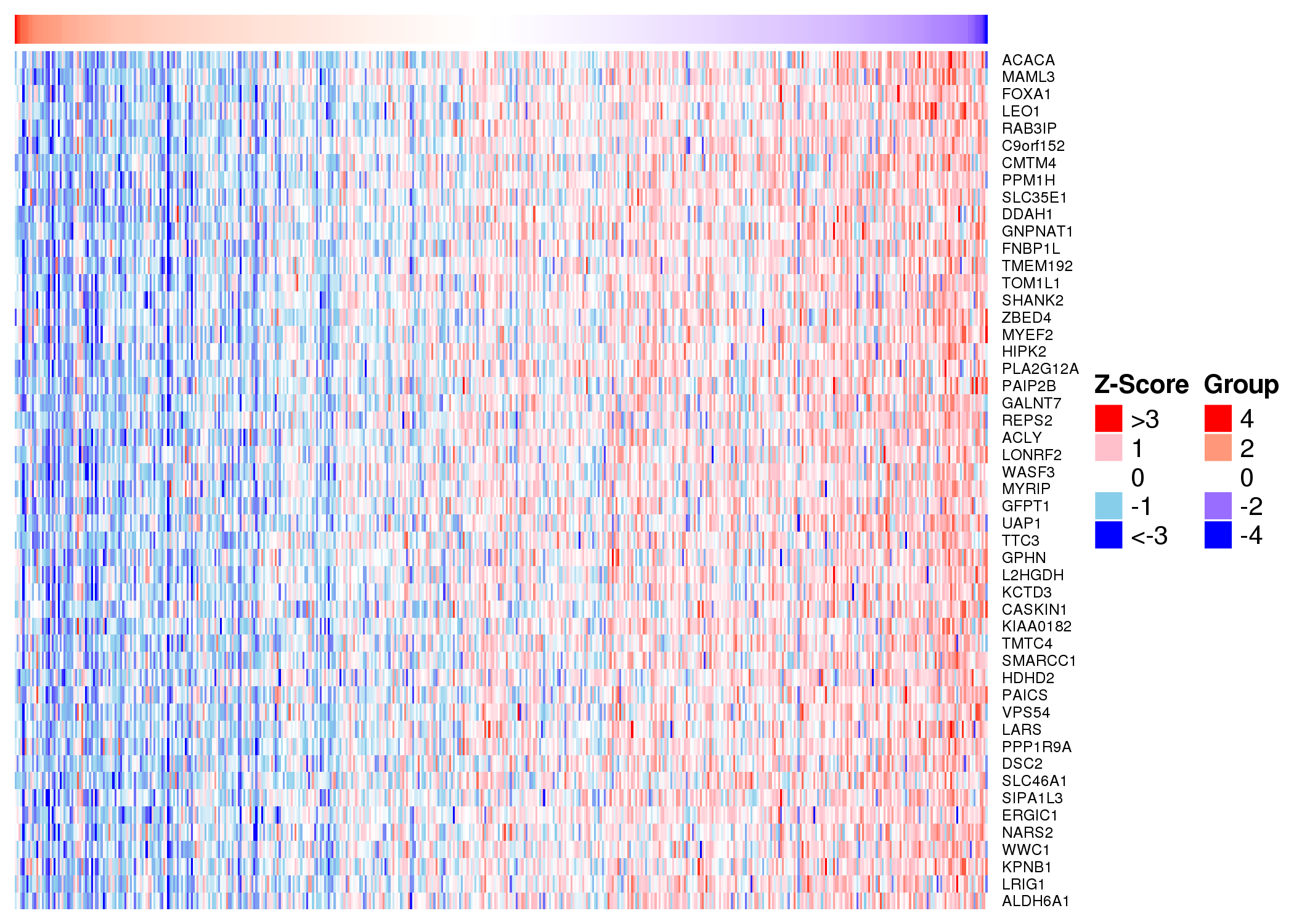

Supplement: Supplementary file 2 [file DataSheet_2.zip › raw data 2/Fig 9/fig 9c.png]

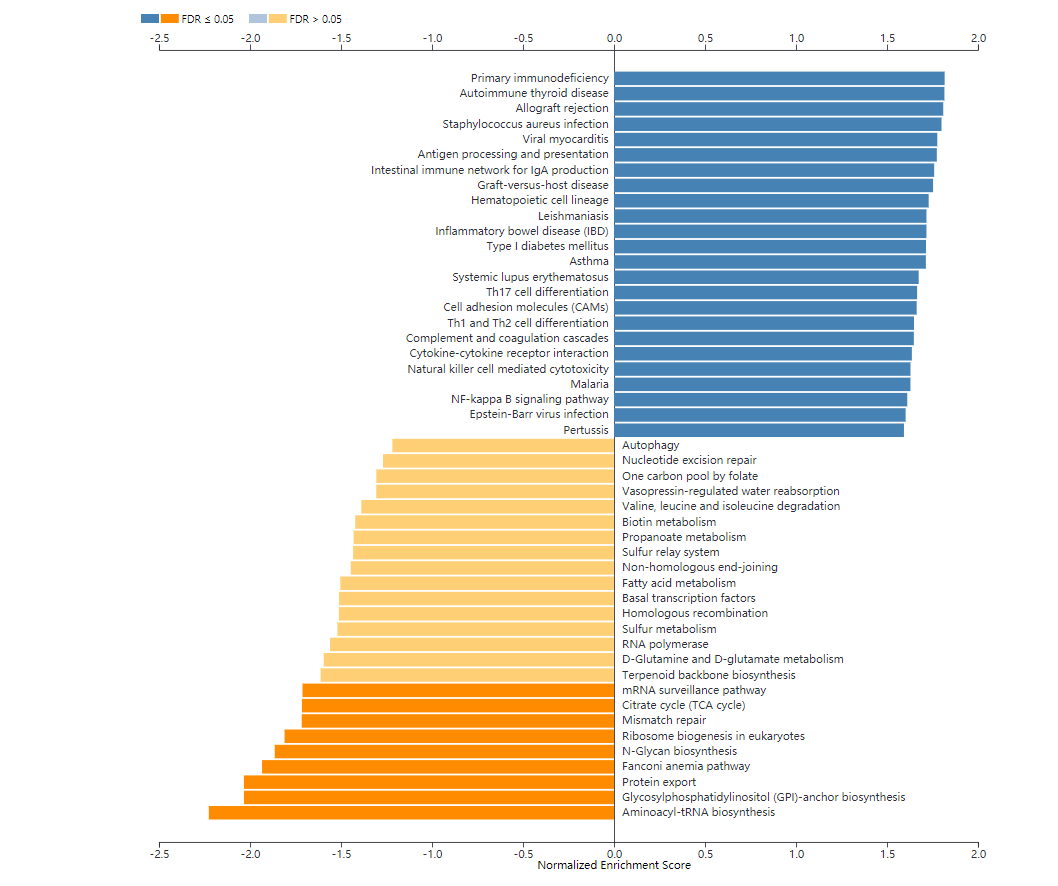

Supplement: Supplementary file 2 [file DataSheet_2.zip › raw data 2/Fig 9/fig 9d.png]

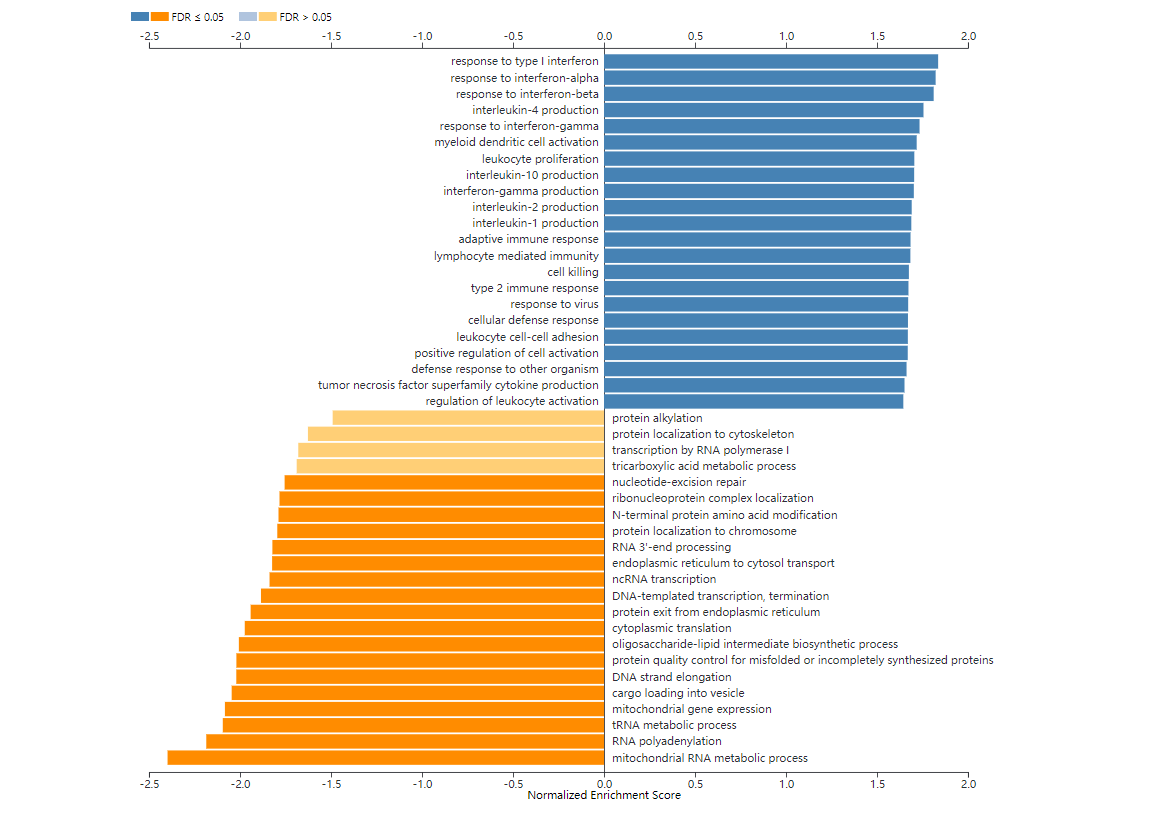

Supplement: Supplementary file 2 [file DataSheet_2.zip › raw data 2/Fig 9/fig 9e.png]

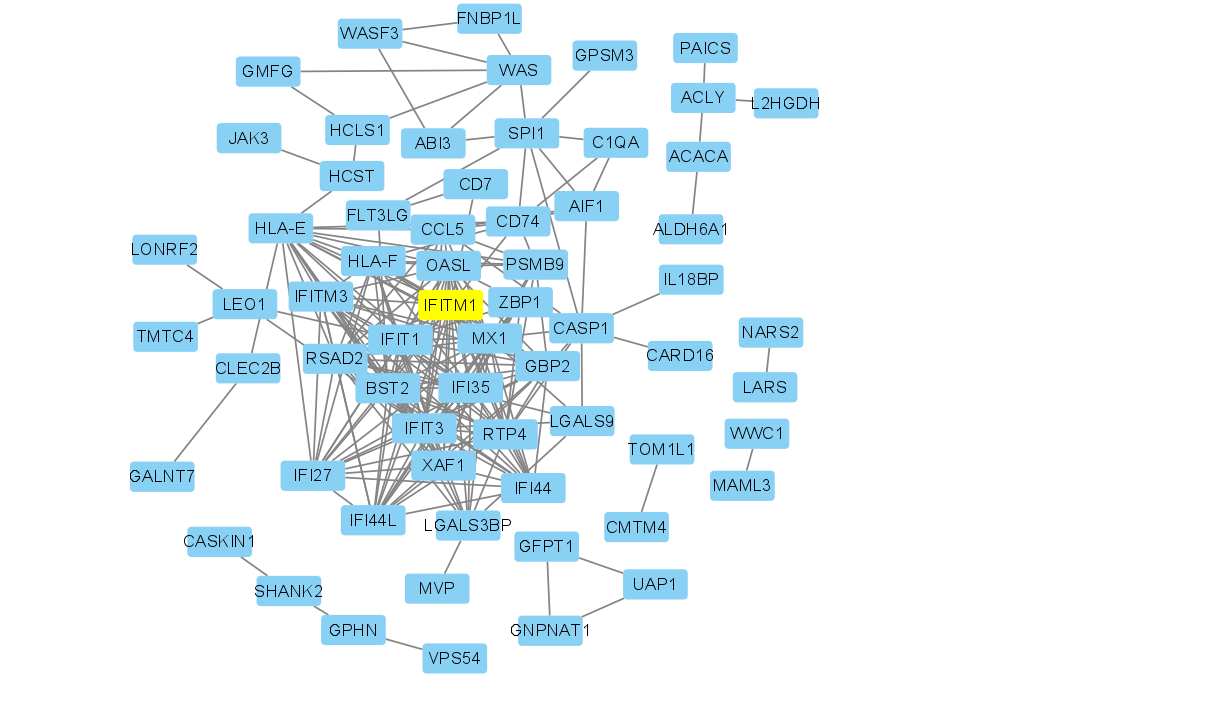

Supplement: Supplementary file 2 [file DataSheet_2.zip › raw data 2/fig 10/fig 10a.png]

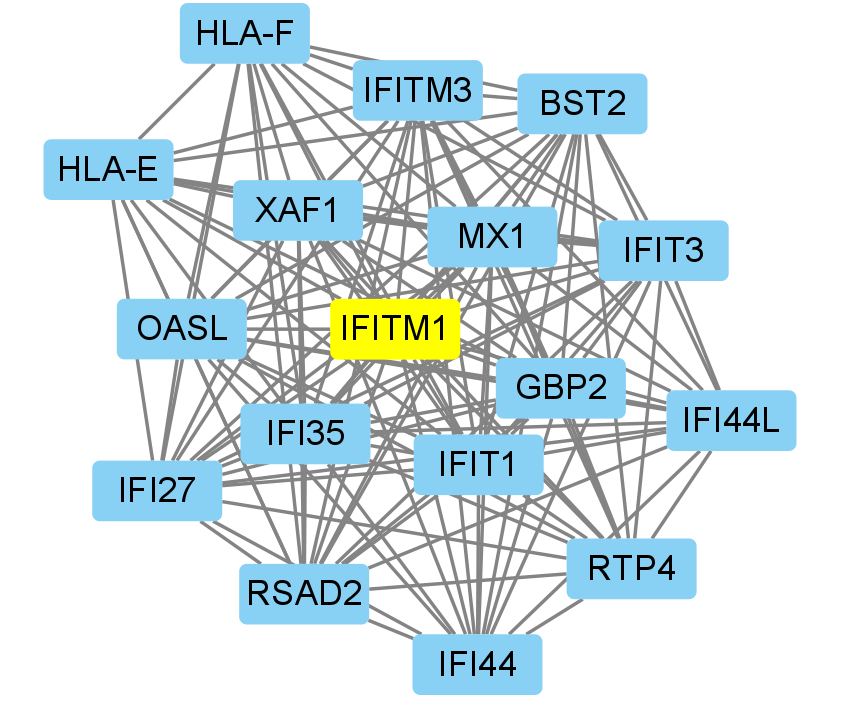

Supplement: Supplementary file 2 [file DataSheet_2.zip › raw data 2/fig 10/fig 10b.png]

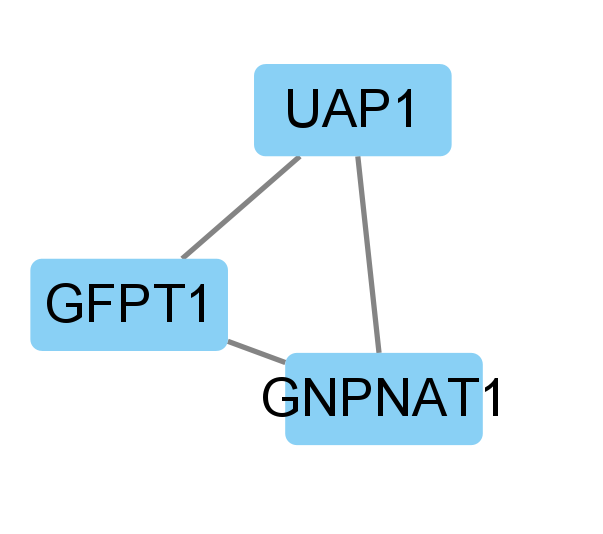

Supplement: Supplementary file 2 [file DataSheet_2.zip › raw data 2/fig 10/fig 10c.png]

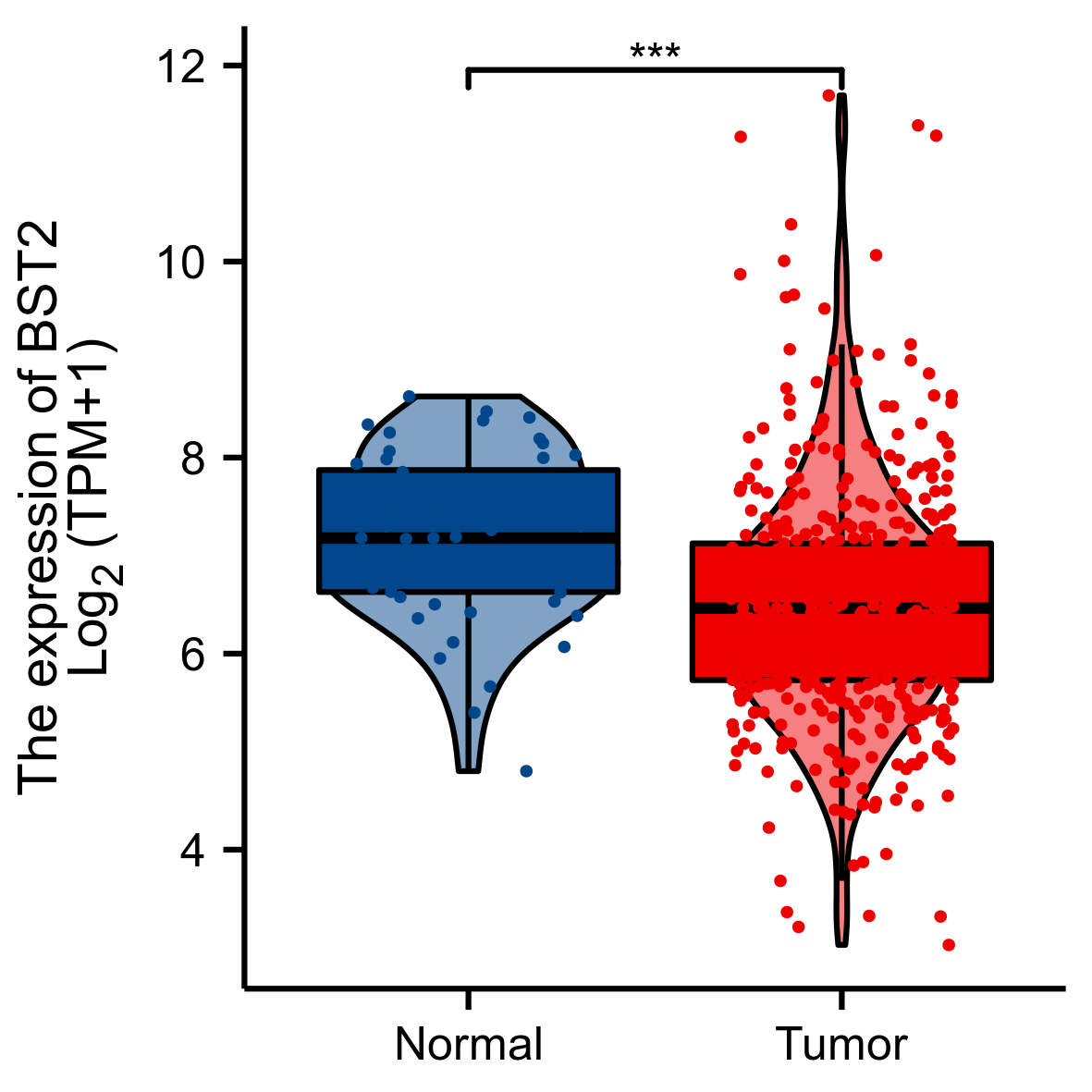

Supplement: Supplementary file 2 [file DataSheet_2.zip › raw data 2/fig 11/▒φ┤∩▓ε╥∞_╖╟┼Σ╢╘╤∙▒╛_BST2.tiff]

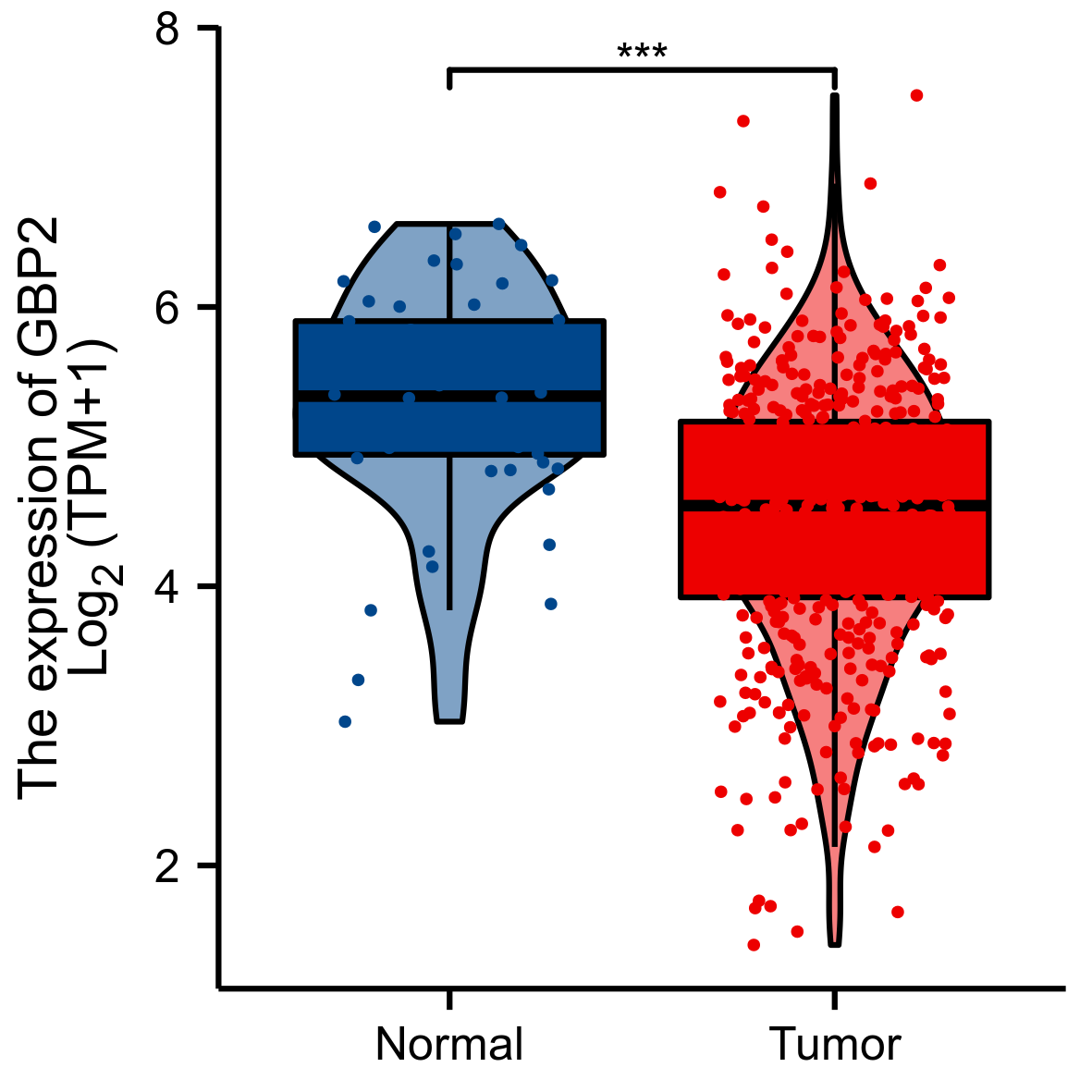

Supplement: Supplementary file 2 [file DataSheet_2.zip › raw data 2/fig 11/▒φ┤∩▓ε╥∞_╖╟┼Σ╢╘╤∙▒╛_GBP2.tiff]

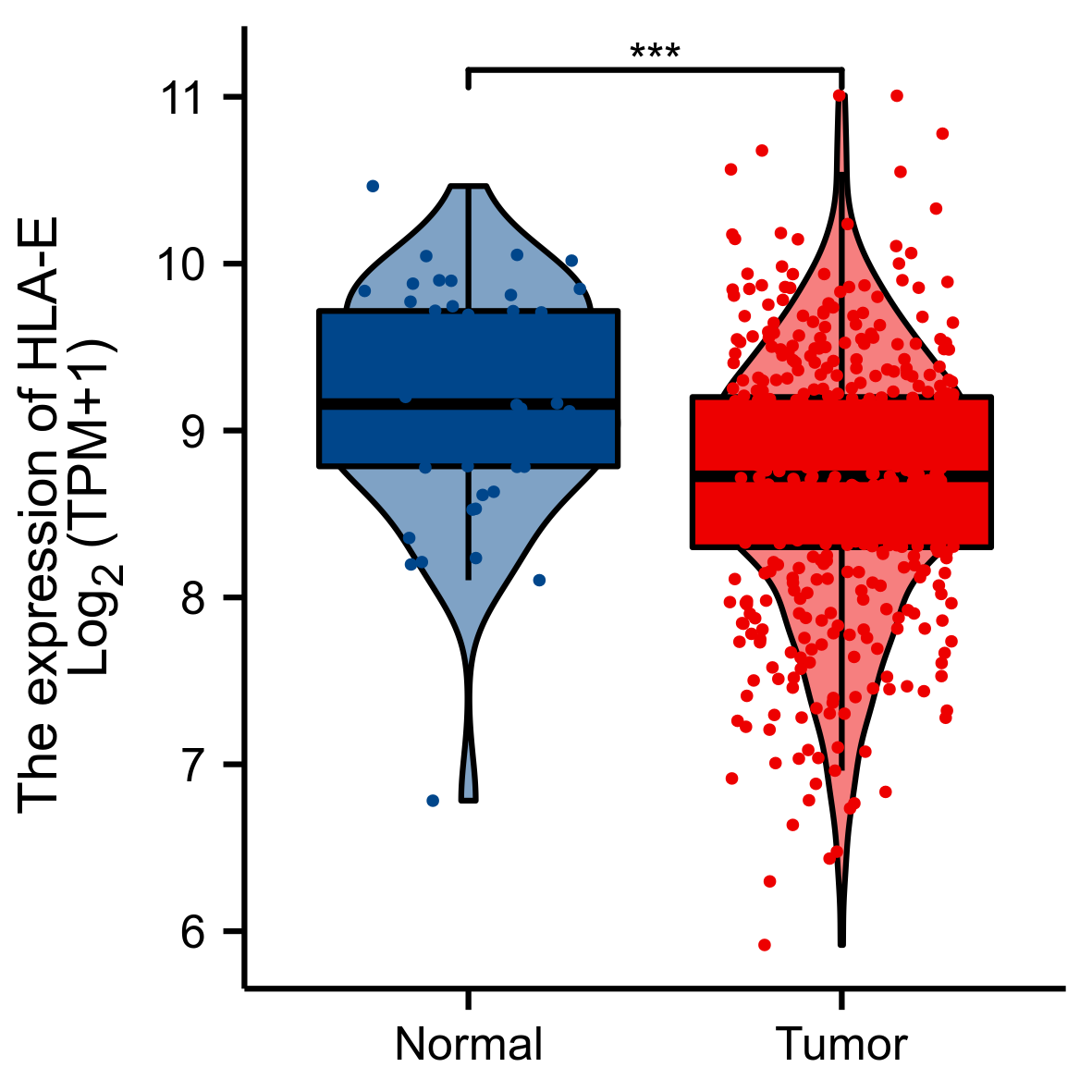

Supplement: Supplementary file 2 [file DataSheet_2.zip › raw data 2/fig 11/▒φ┤∩▓ε╥∞_╖╟┼Σ╢╘╤∙▒╛_HLA-E.tiff]

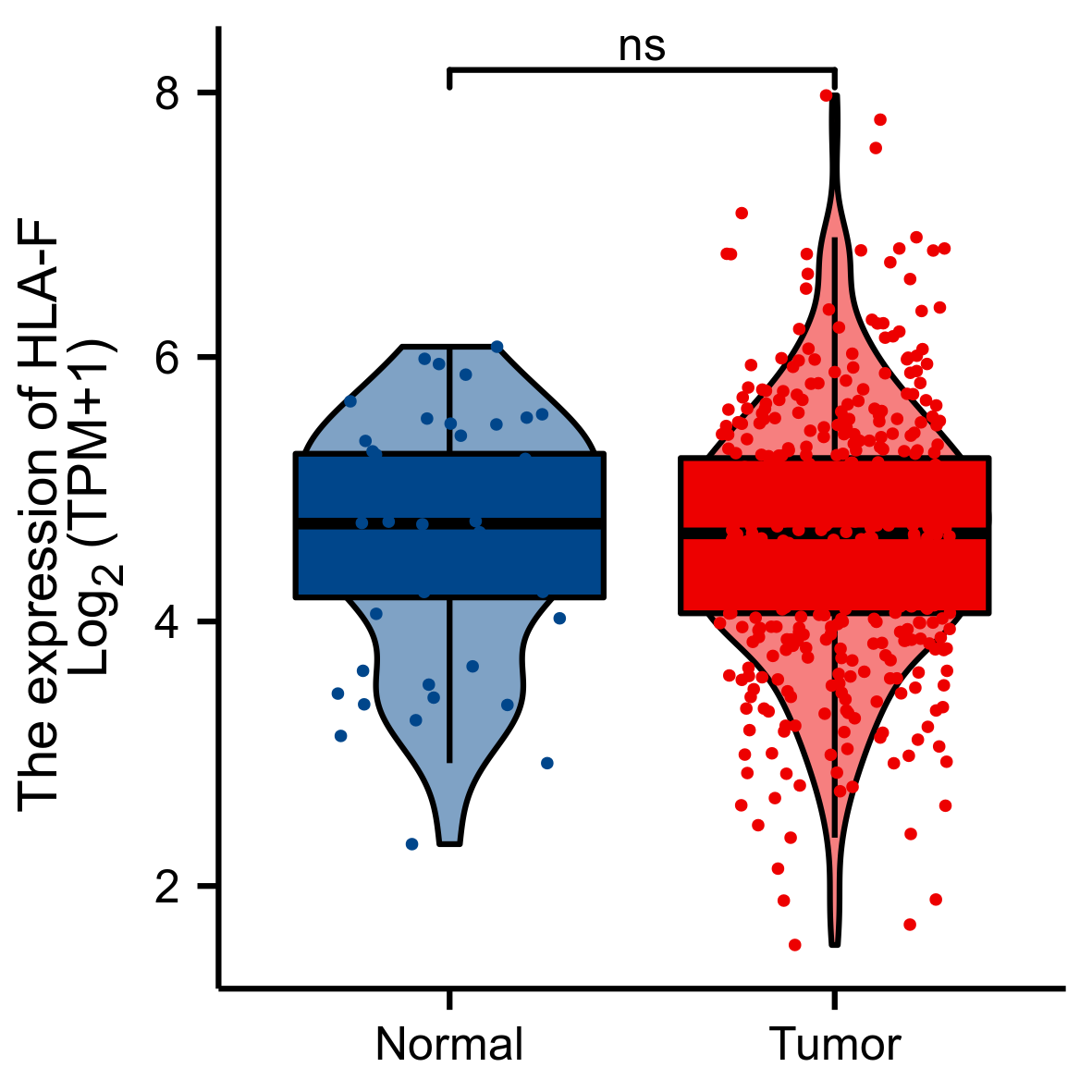

Supplement: Supplementary file 2 [file DataSheet_2.zip › raw data 2/fig 11/▒φ┤∩▓ε╥∞_╖╟┼Σ╢╘╤∙▒╛_HLA-F.tiff]

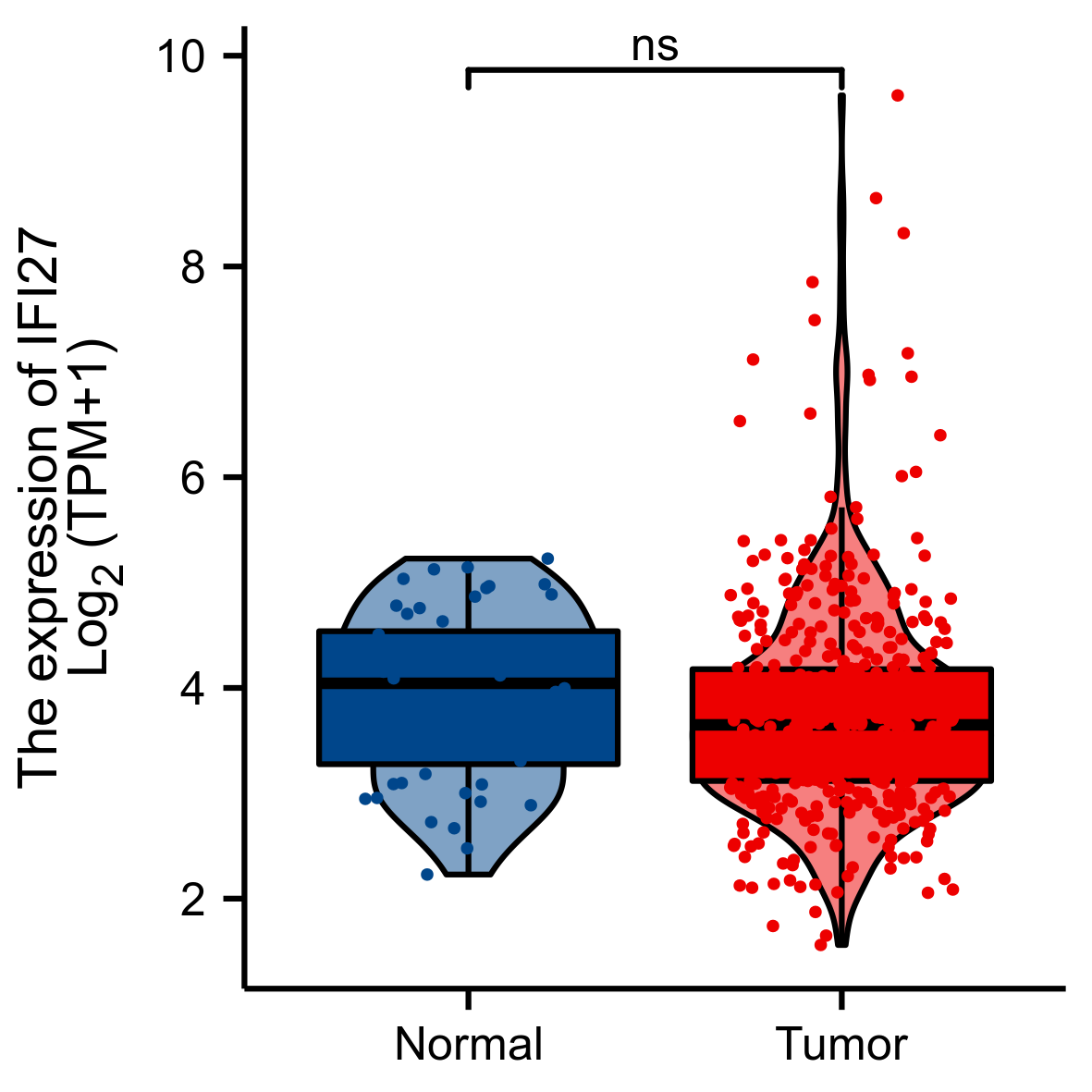

Supplement: Supplementary file 2 [file DataSheet_2.zip › raw data 2/fig 11/▒φ┤∩▓ε╥∞_╖╟┼Σ╢╘╤∙▒╛_IFI27.tiff]

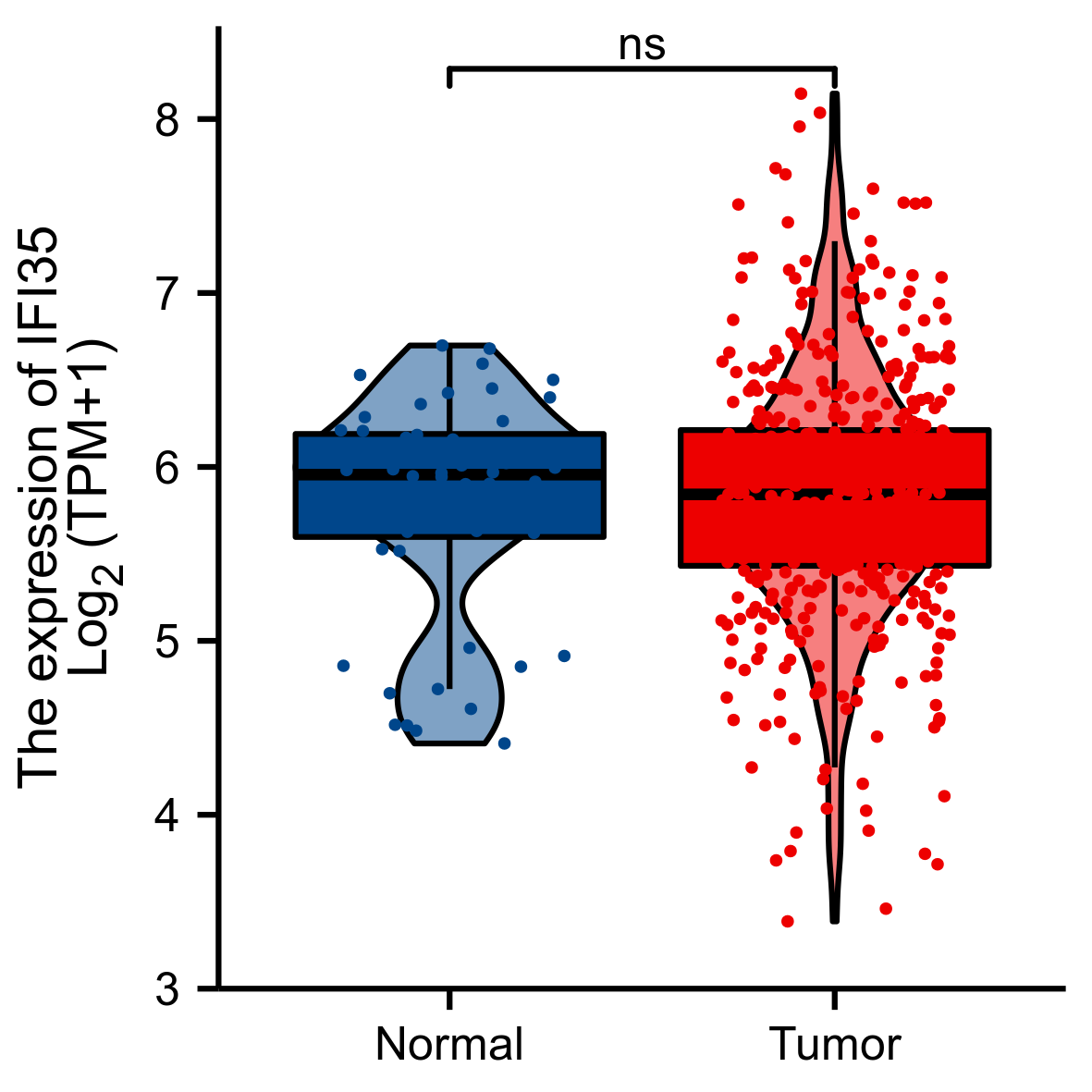

Supplement: Supplementary file 2 [file DataSheet_2.zip › raw data 2/fig 11/▒φ┤∩▓ε╥∞_╖╟┼Σ╢╘╤∙▒╛_IFI35.tiff]

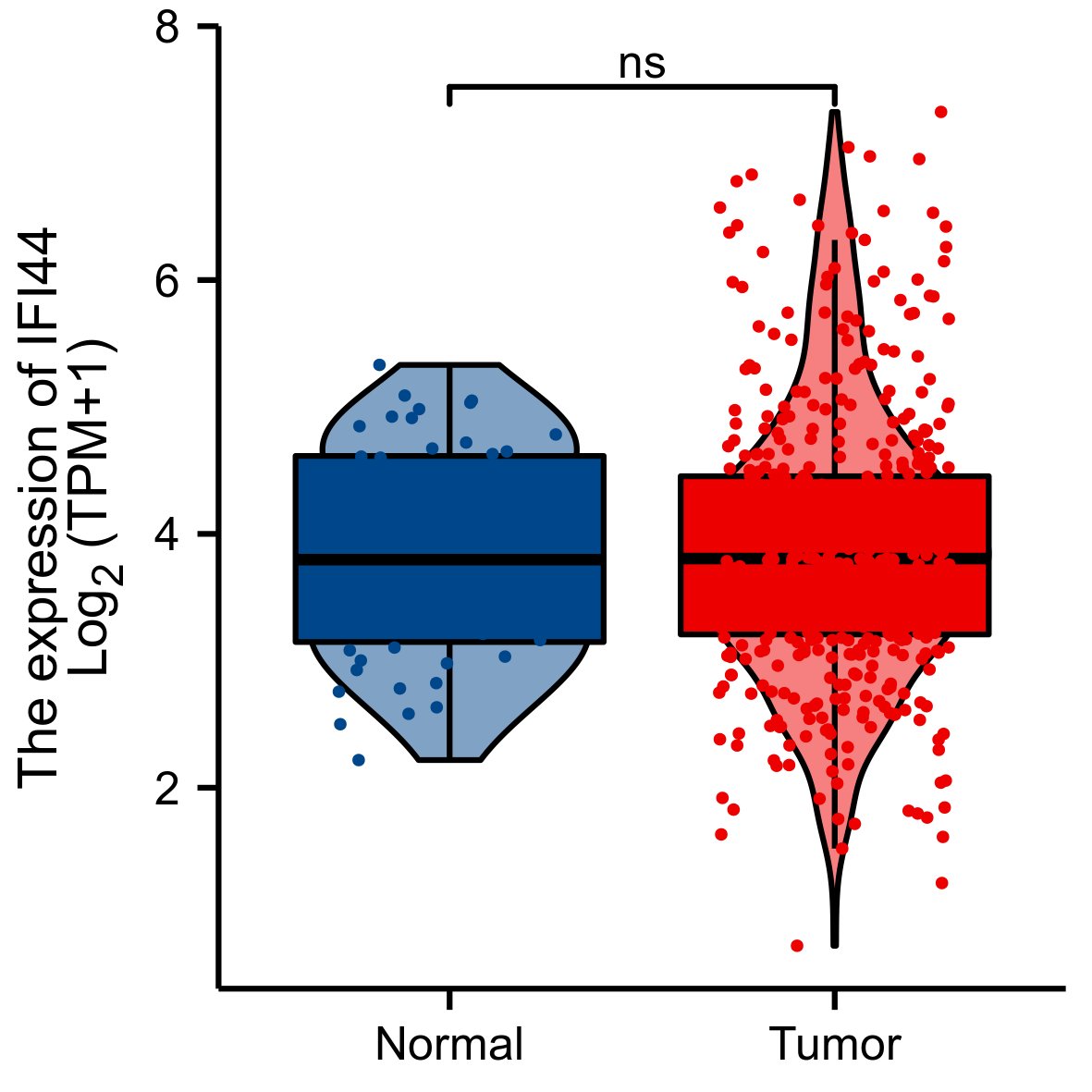

Supplement: Supplementary file 2 [file DataSheet_2.zip › raw data 2/fig 11/▒φ┤∩▓ε╥∞_╖╟┼Σ╢╘╤∙▒╛_IFI44.tiff]

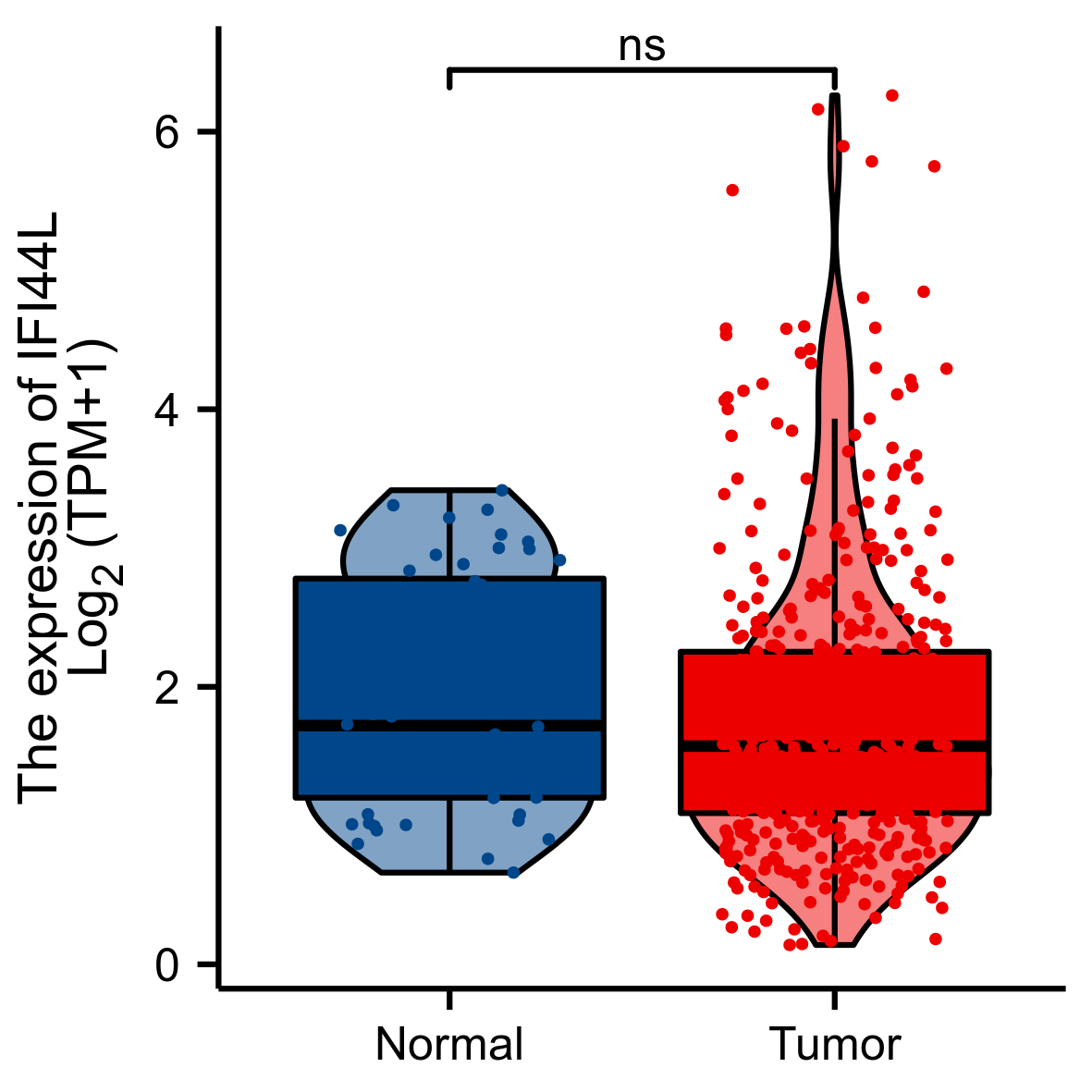

Supplement: Supplementary file 2 [file DataSheet_2.zip › raw data 2/fig 11/▒φ┤∩▓ε╥∞_╖╟┼Σ╢╘╤∙▒╛_IFI44L.tiff]

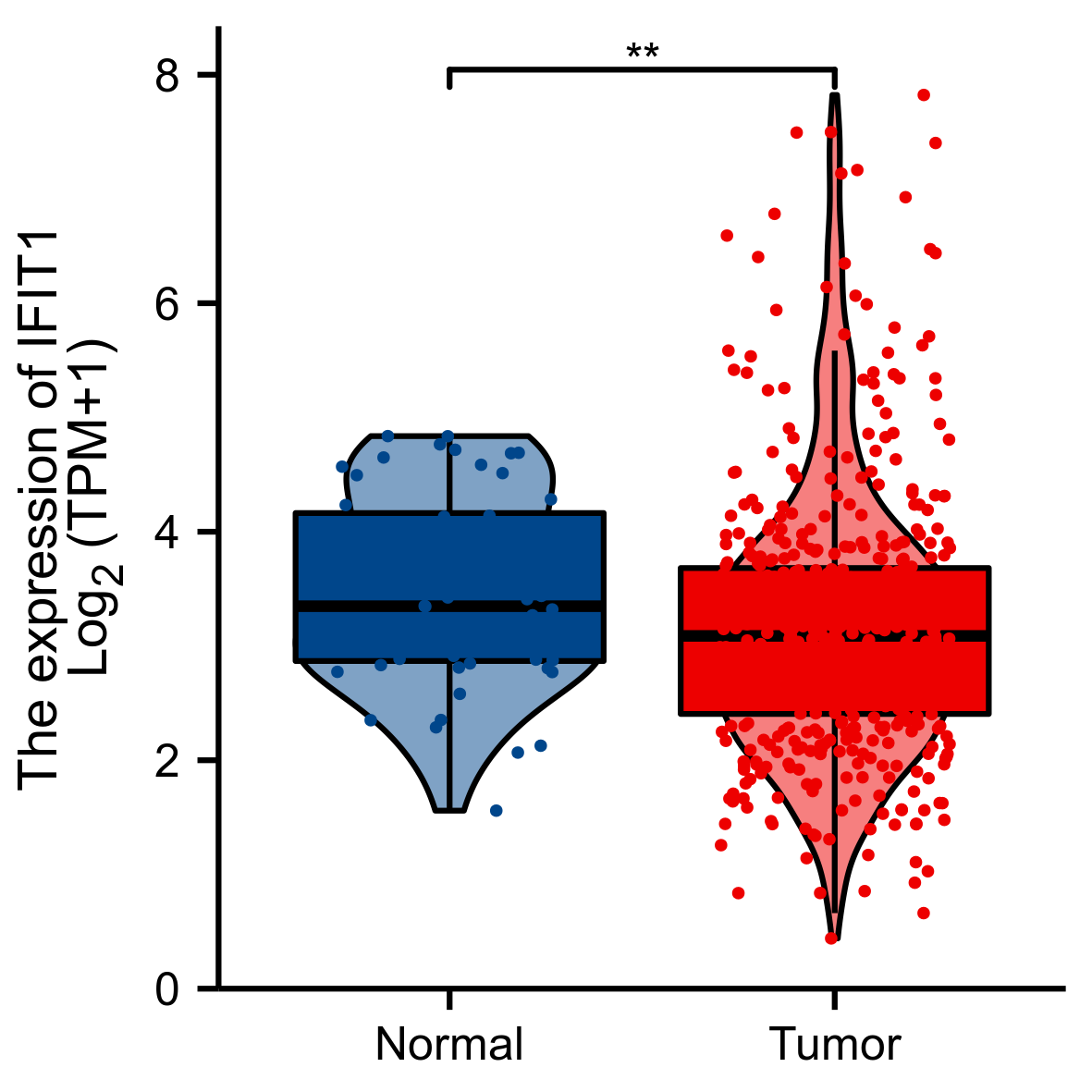

Supplement: Supplementary file 2 [file DataSheet_2.zip › raw data 2/fig 11/▒φ┤∩▓ε╥∞_╖╟┼Σ╢╘╤∙▒╛_IFIT1.tiff]

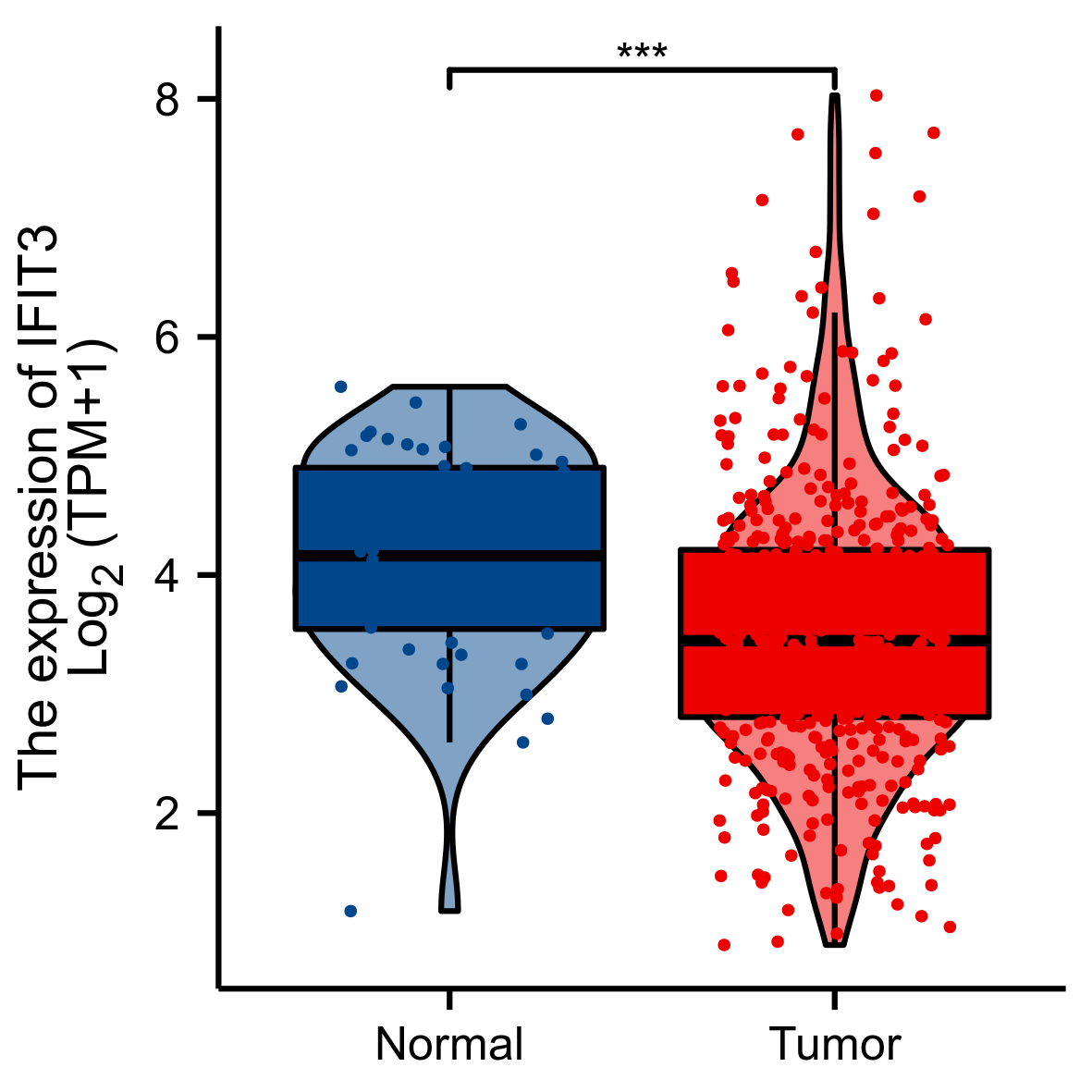

Supplement: Supplementary file 2 [file DataSheet_2.zip › raw data 2/fig 11/▒φ┤∩▓ε╥∞_╖╟┼Σ╢╘╤∙▒╛_IFIT3.tiff]

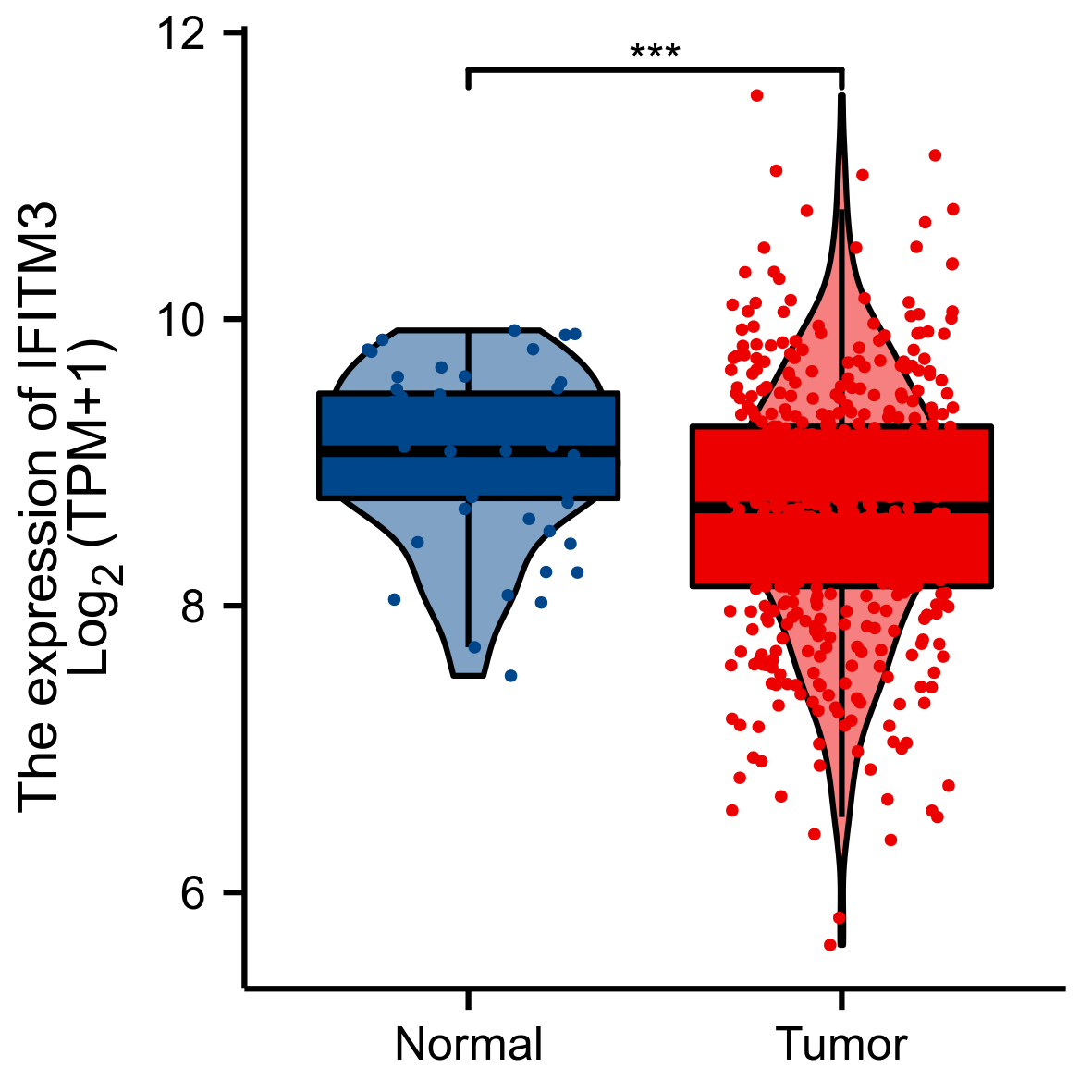

Supplement: Supplementary file 2 [file DataSheet_2.zip › raw data 2/fig 11/▒φ┤∩▓ε╥∞_╖╟┼Σ╢╘╤∙▒╛_IFITM3.tiff]

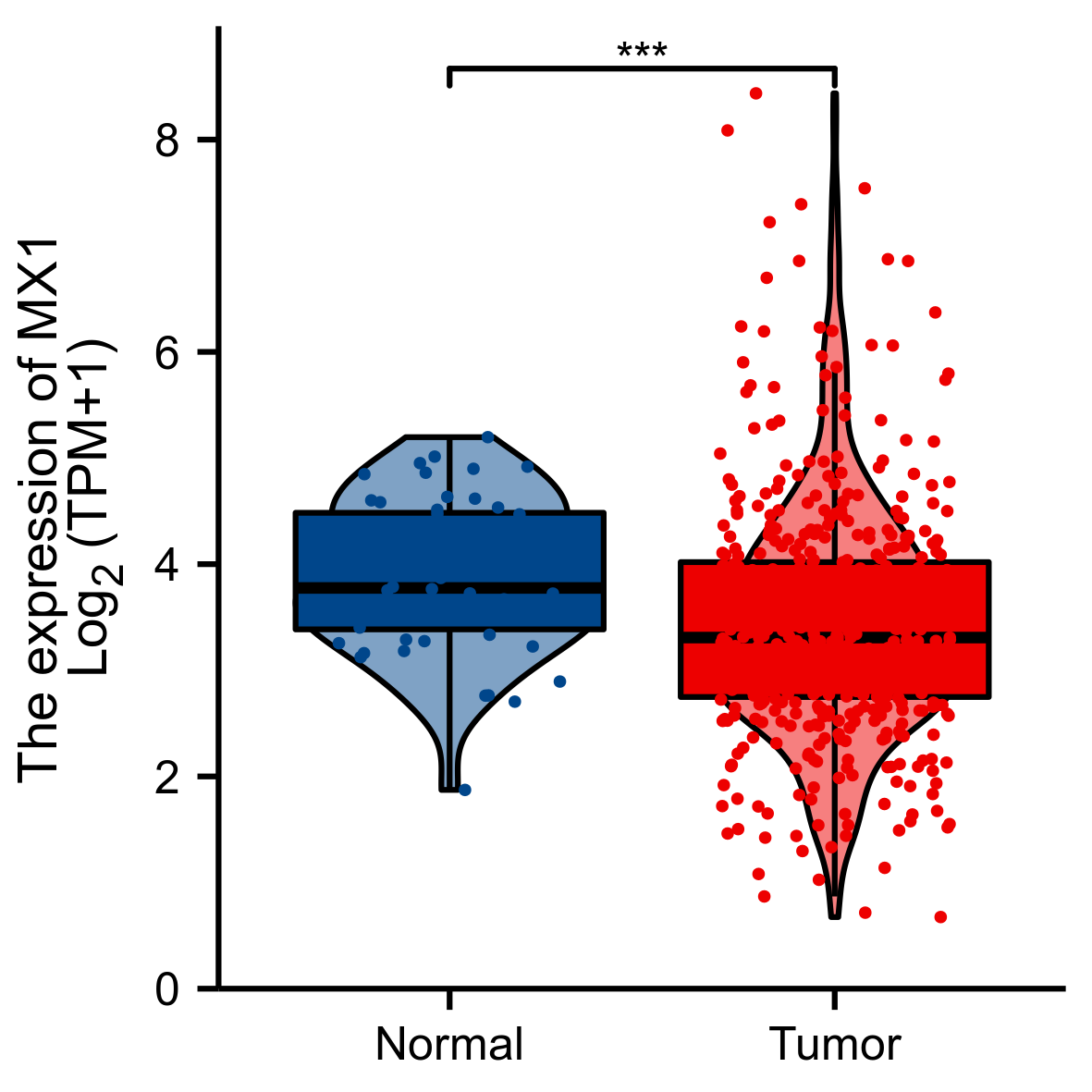

Supplement: Supplementary file 2 [file DataSheet_2.zip › raw data 2/fig 11/▒φ┤∩▓ε╥∞_╖╟┼Σ╢╘╤∙▒╛_MX1.tiff]

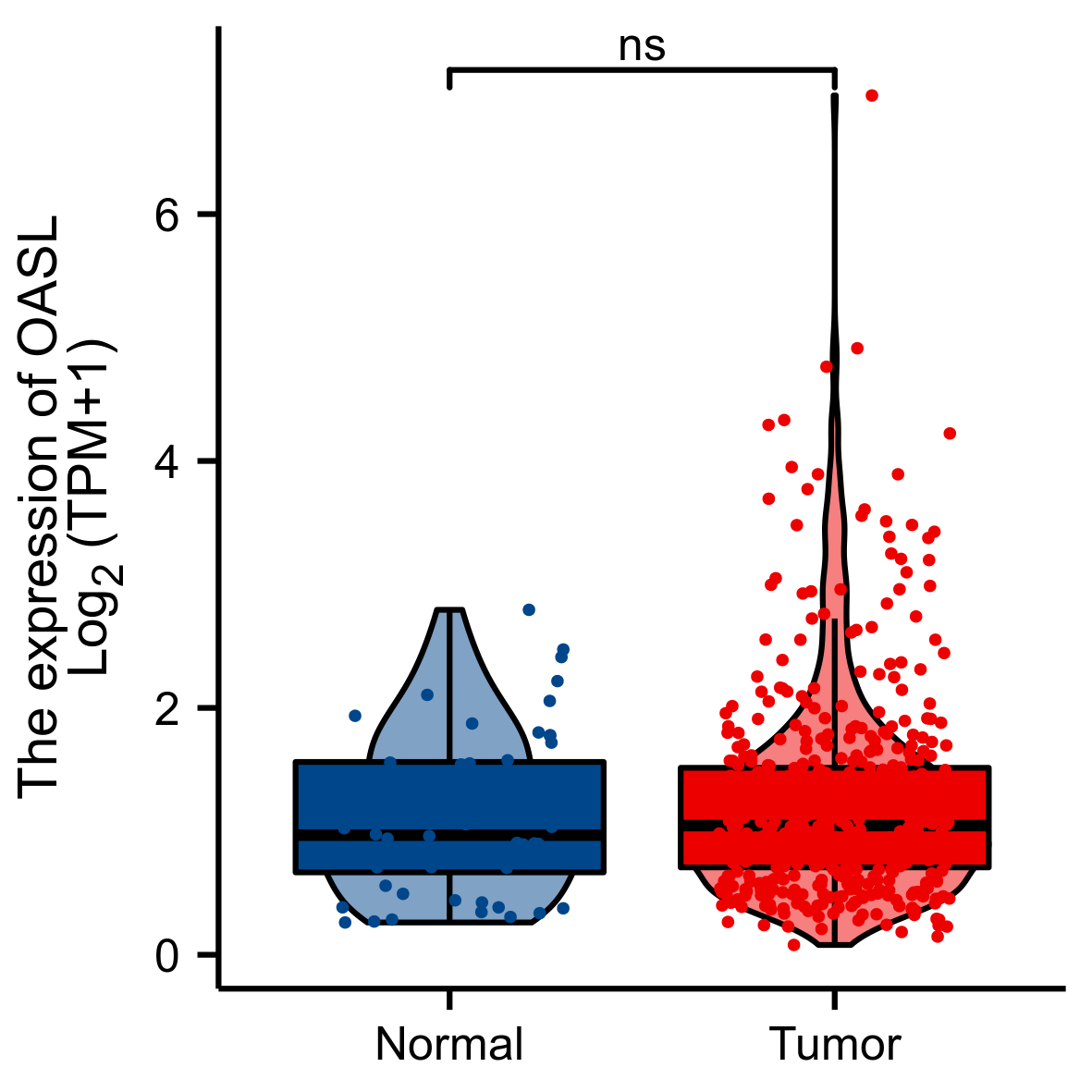

Supplement: Supplementary file 2 [file DataSheet_2.zip › raw data 2/fig 11/▒φ┤∩▓ε╥∞_╖╟┼Σ╢╘╤∙▒╛_OASL.tiff]

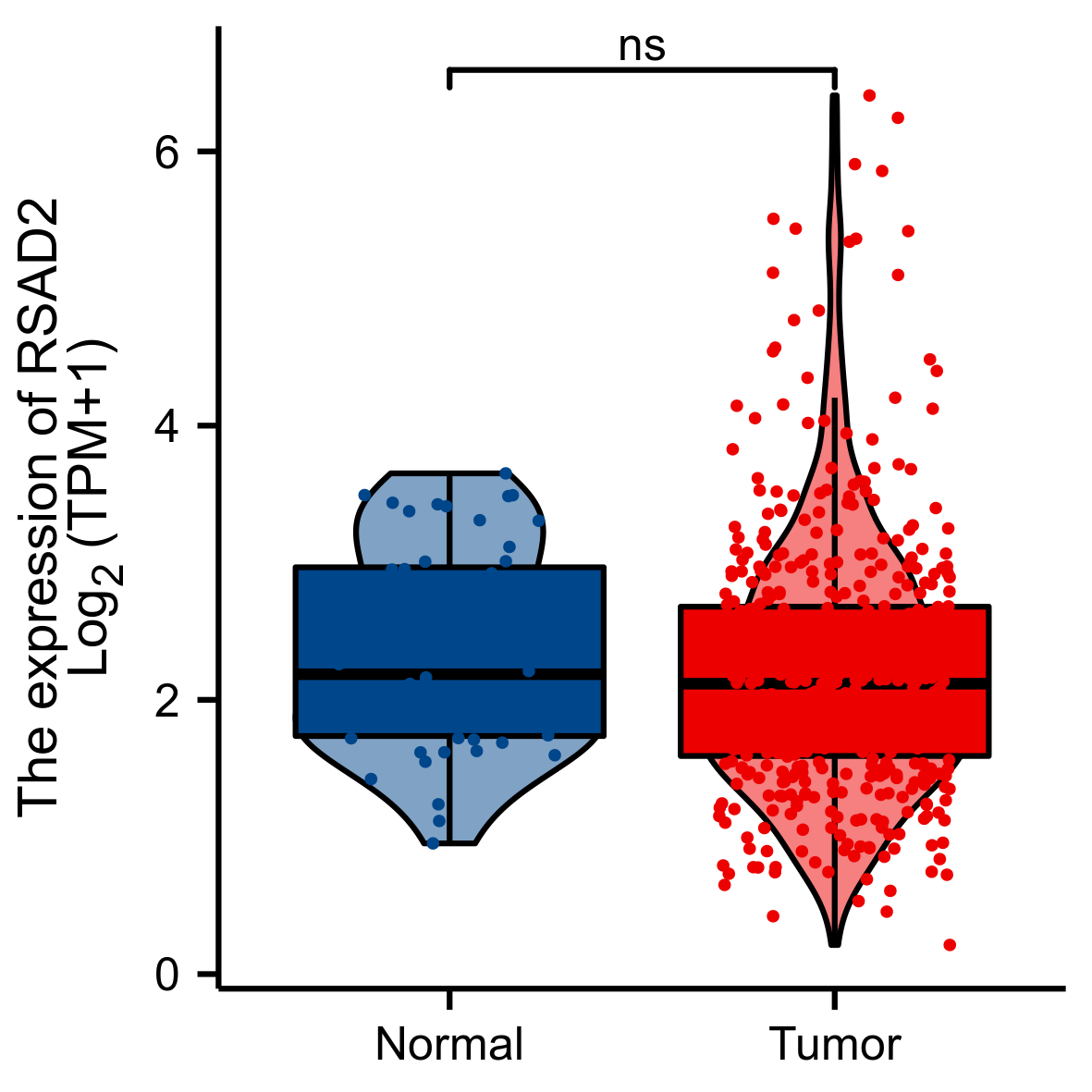

Supplement: Supplementary file 2 [file DataSheet_2.zip › raw data 2/fig 11/▒φ┤∩▓ε╥∞_╖╟┼Σ╢╘╤∙▒╛_RSAD2.tiff]

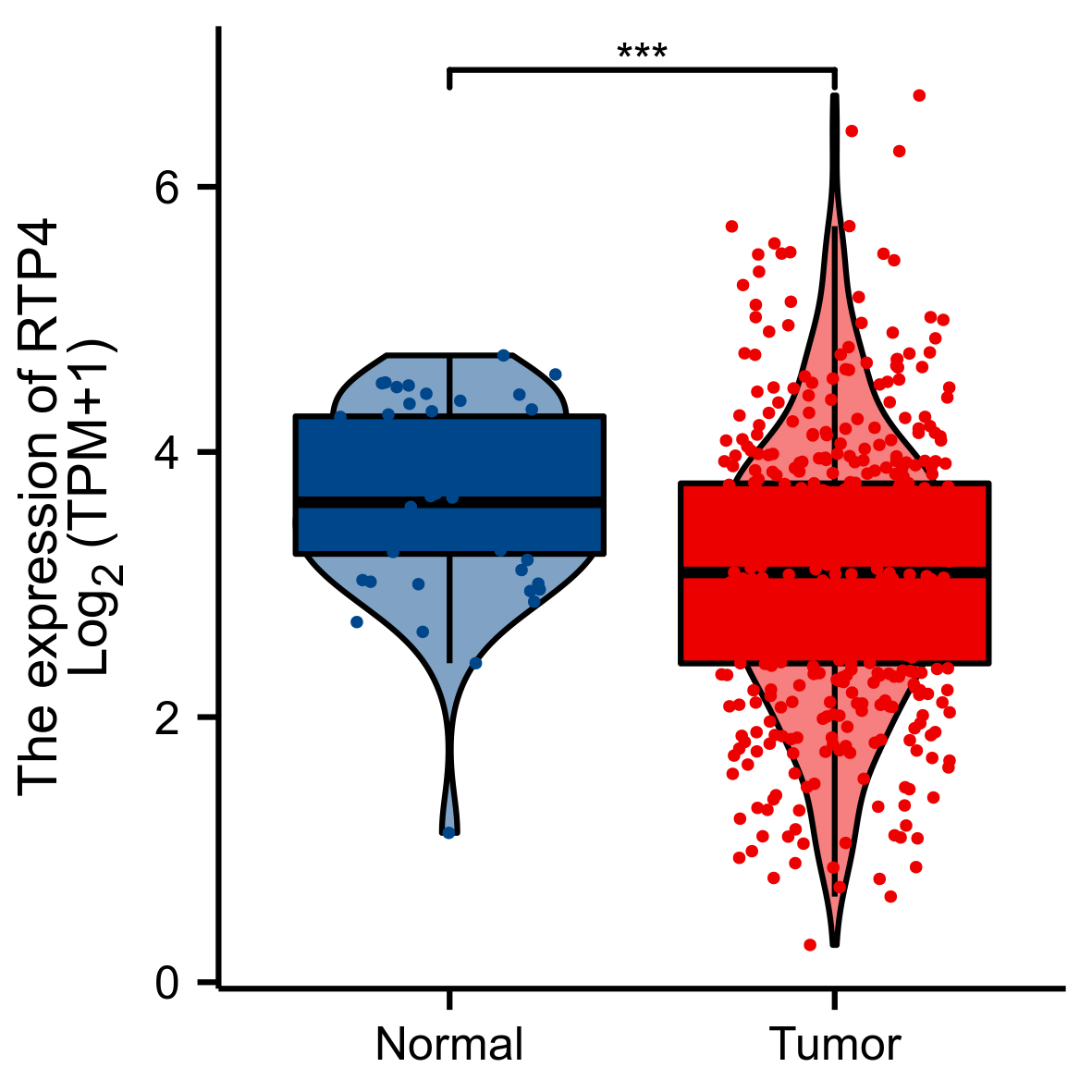

Supplement: Supplementary file 2 [file DataSheet_2.zip › raw data 2/fig 11/▒φ┤∩▓ε╥∞_╖╟┼Σ╢╘╤∙▒╛_RTP4.tiff]

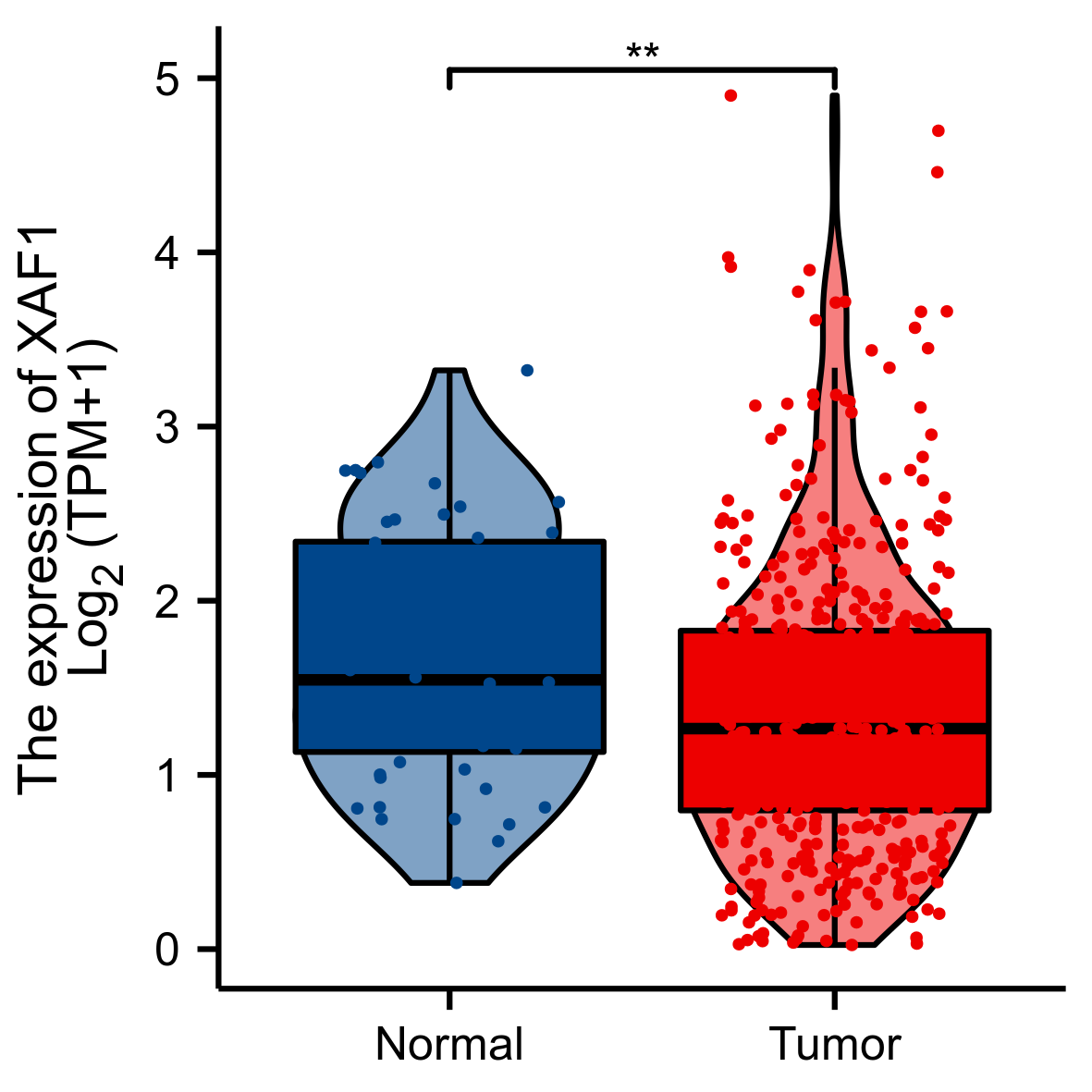

Supplement: Supplementary file 2 [file DataSheet_2.zip › raw data 2/fig 11/▒φ┤∩▓ε╥∞_╖╟┼Σ╢╘╤∙▒╛_XAF1.tiff]

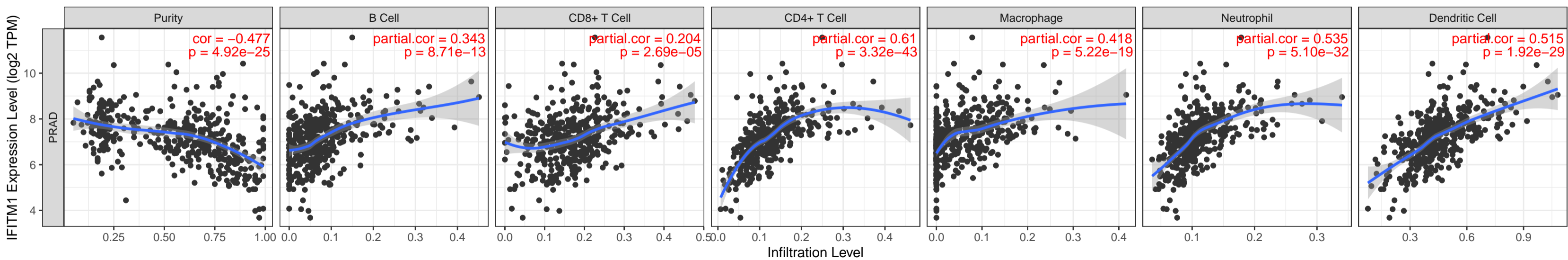

Supplement: Supplementary file 2 [file DataSheet_2.zip › raw data 2/fig 12/fig 12a.pdf]

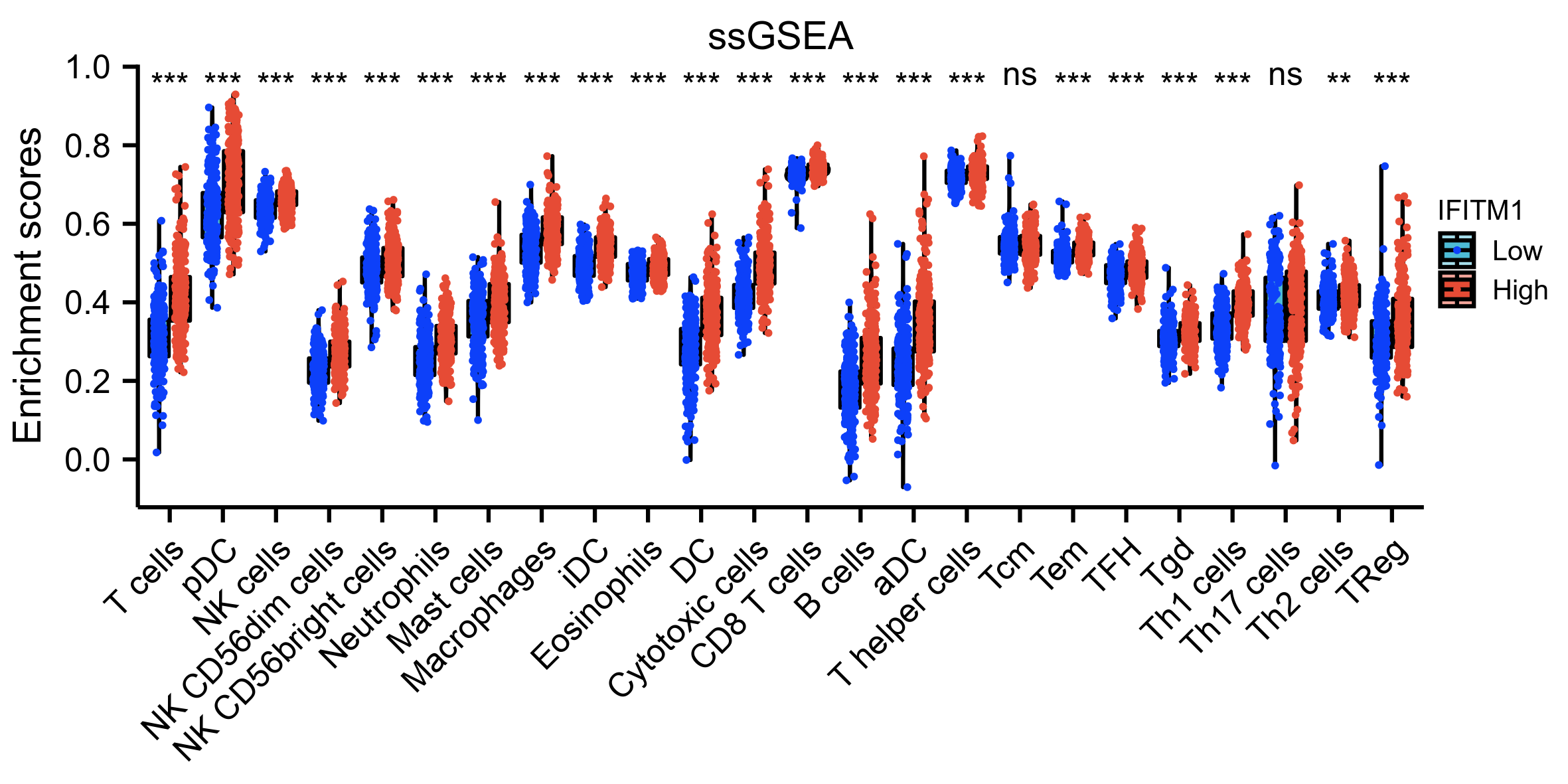

Supplement: Supplementary file 2 [file DataSheet_2.zip › raw data 2/fig 12/fig 12b.tiff]

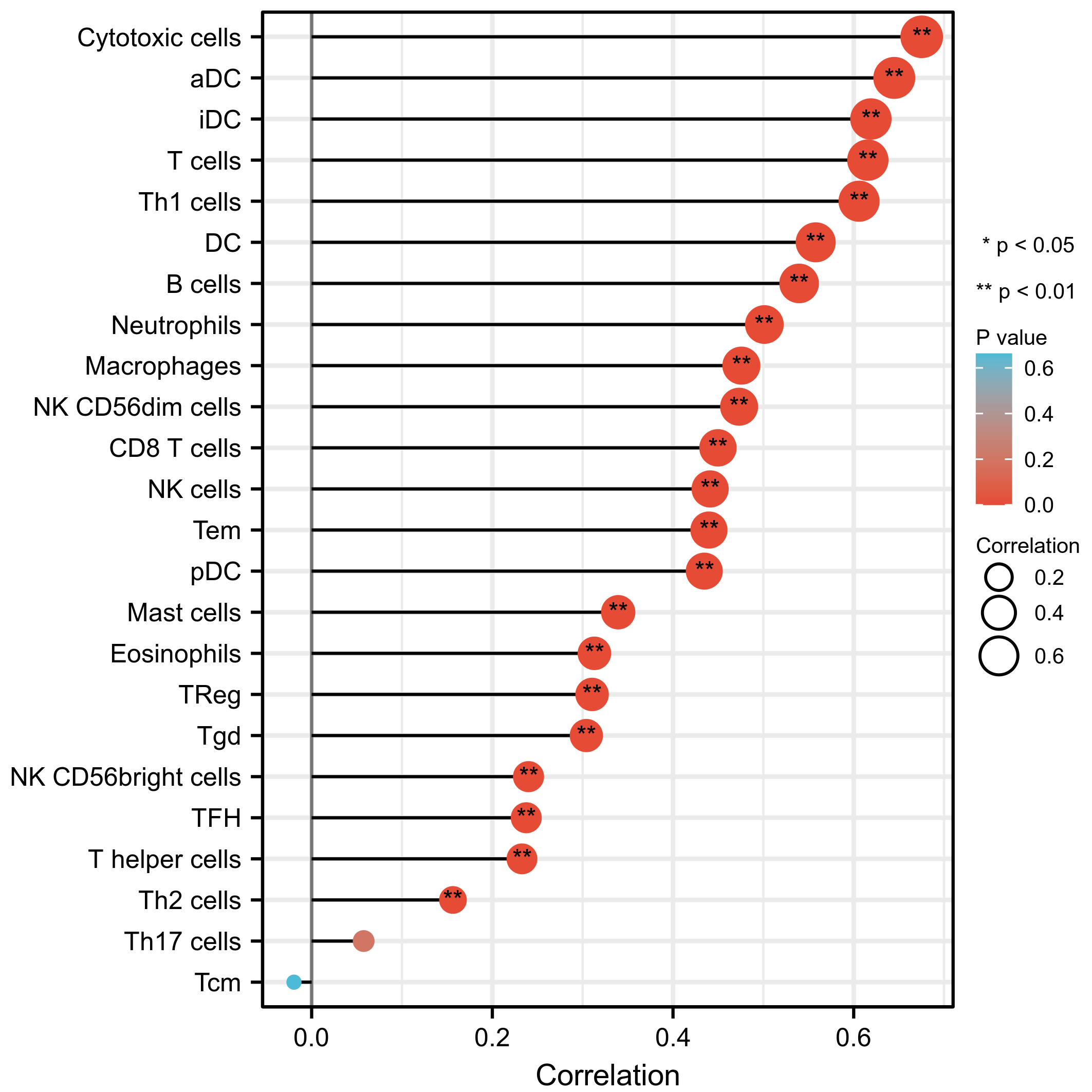

Supplement: Supplementary file 2 [file DataSheet_2.zip › raw data 2/fig 12/fig 12c.tiff]

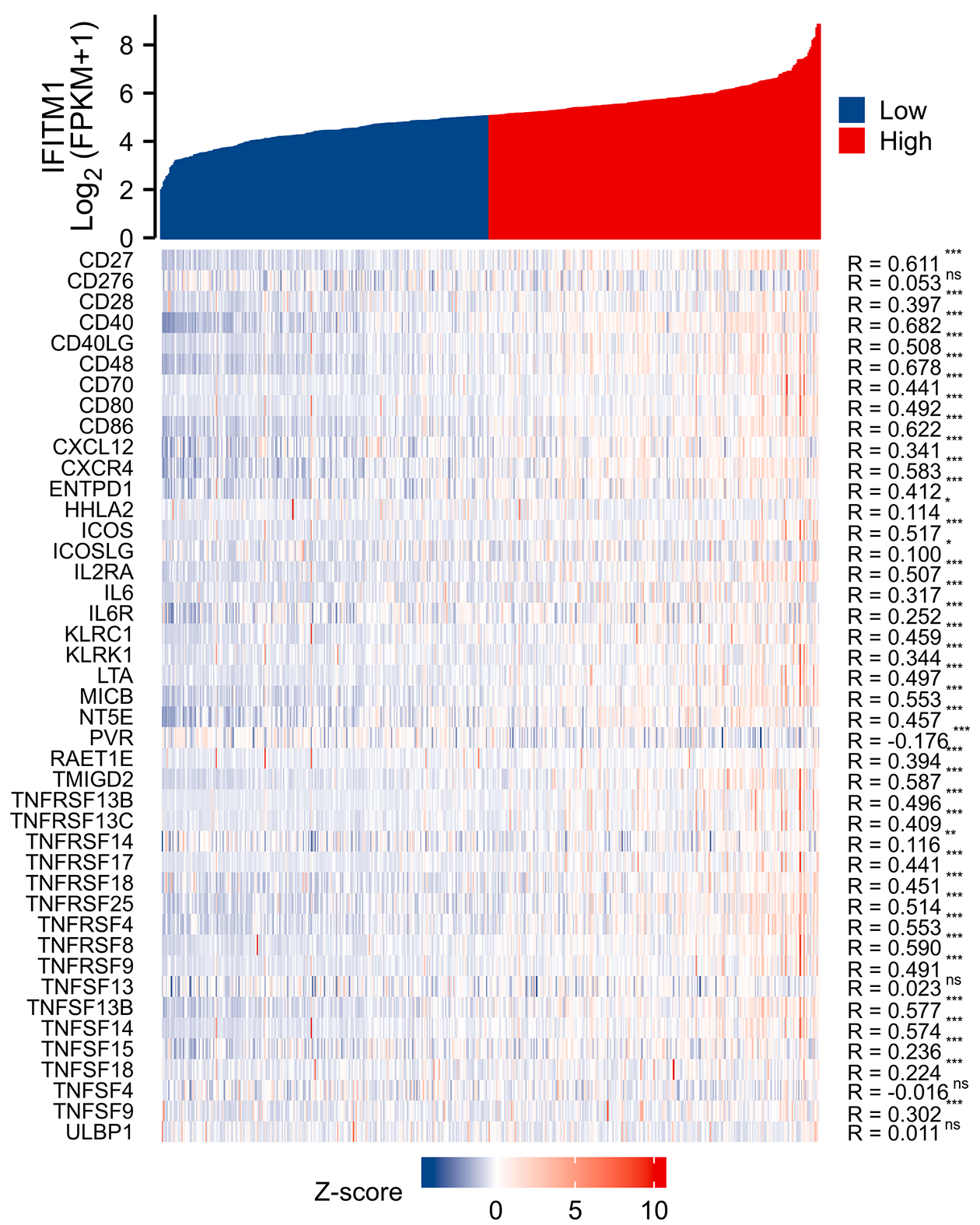

Supplement: Supplementary file 2 [file DataSheet_2.zip › raw data 2/fig 13/fig 13a.tif]

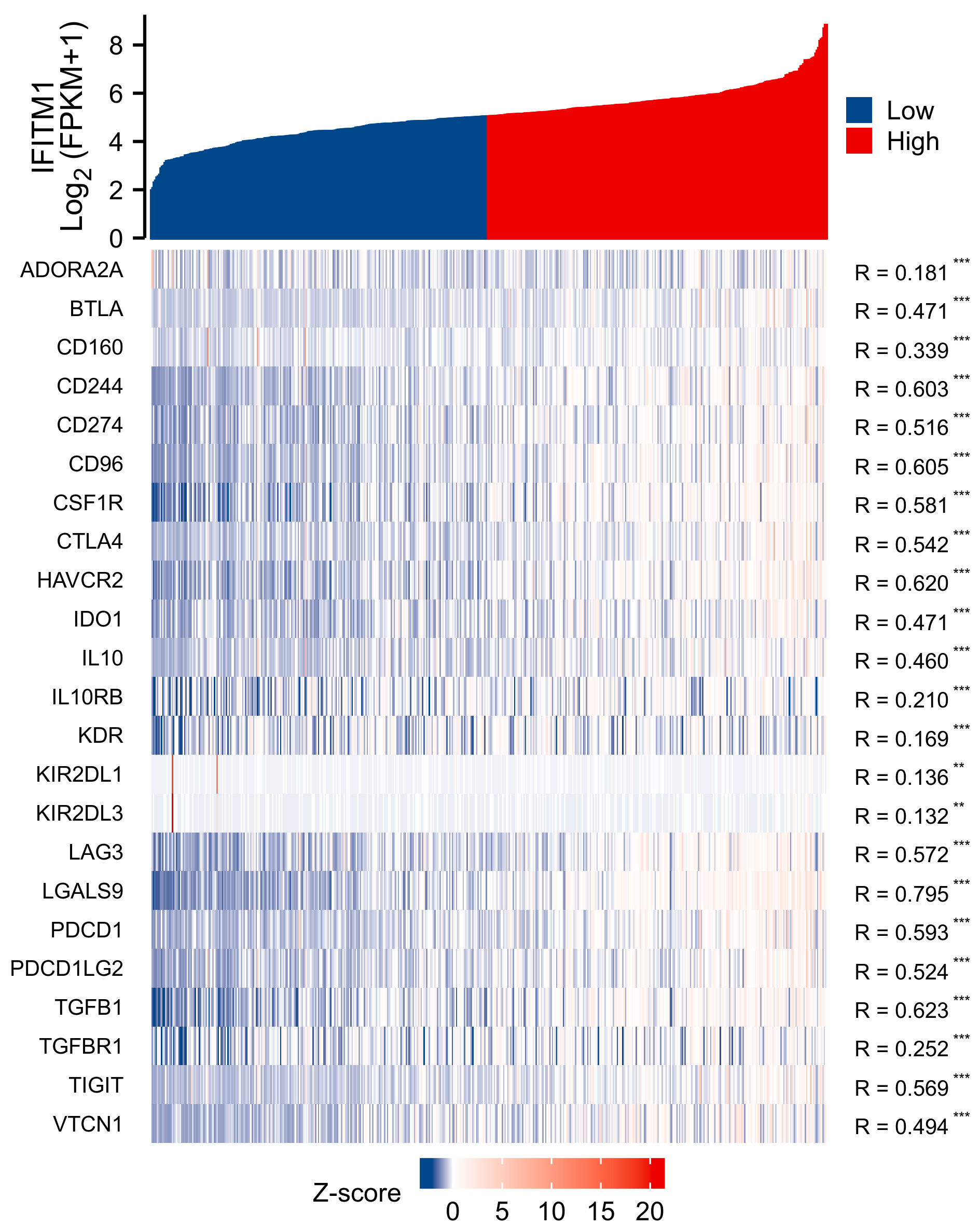

Supplement: Supplementary file 2 [file DataSheet_2.zip › raw data 2/fig 13/fig 13b.tiff]

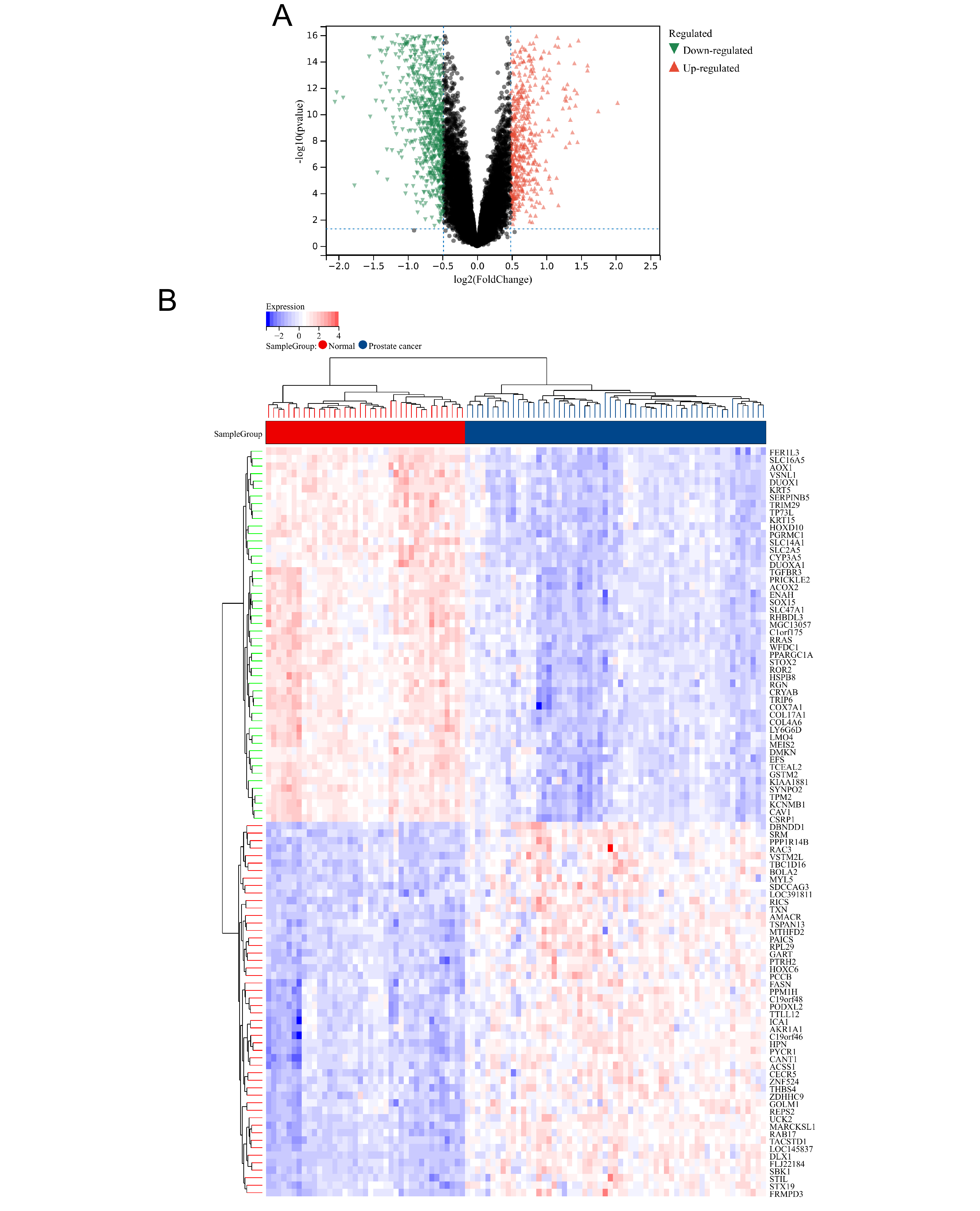

Supplement: Supplementary file 4 [file Image_1.tiff]

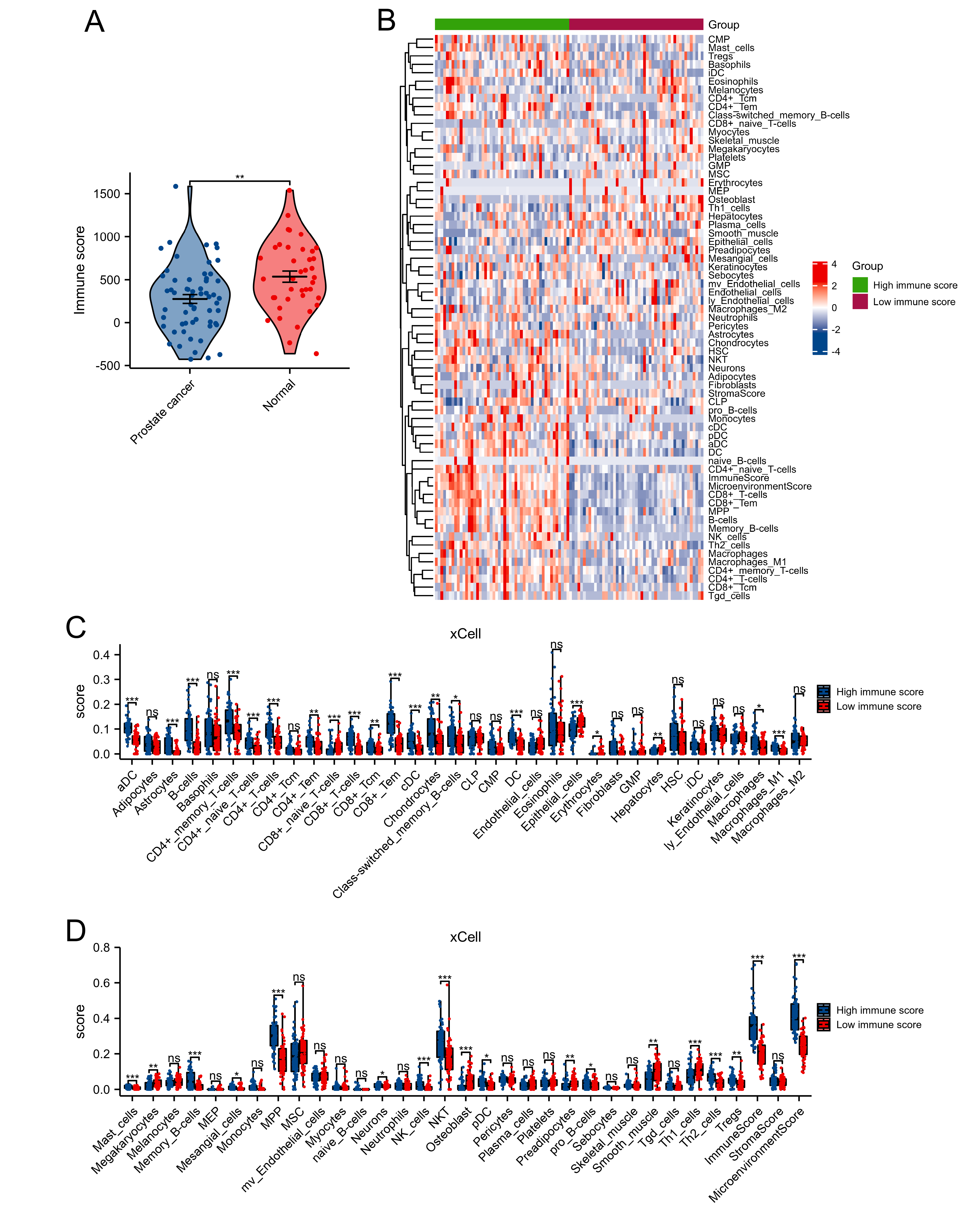

Supplement: Supplementary file 5 [file Image_2.tiff]

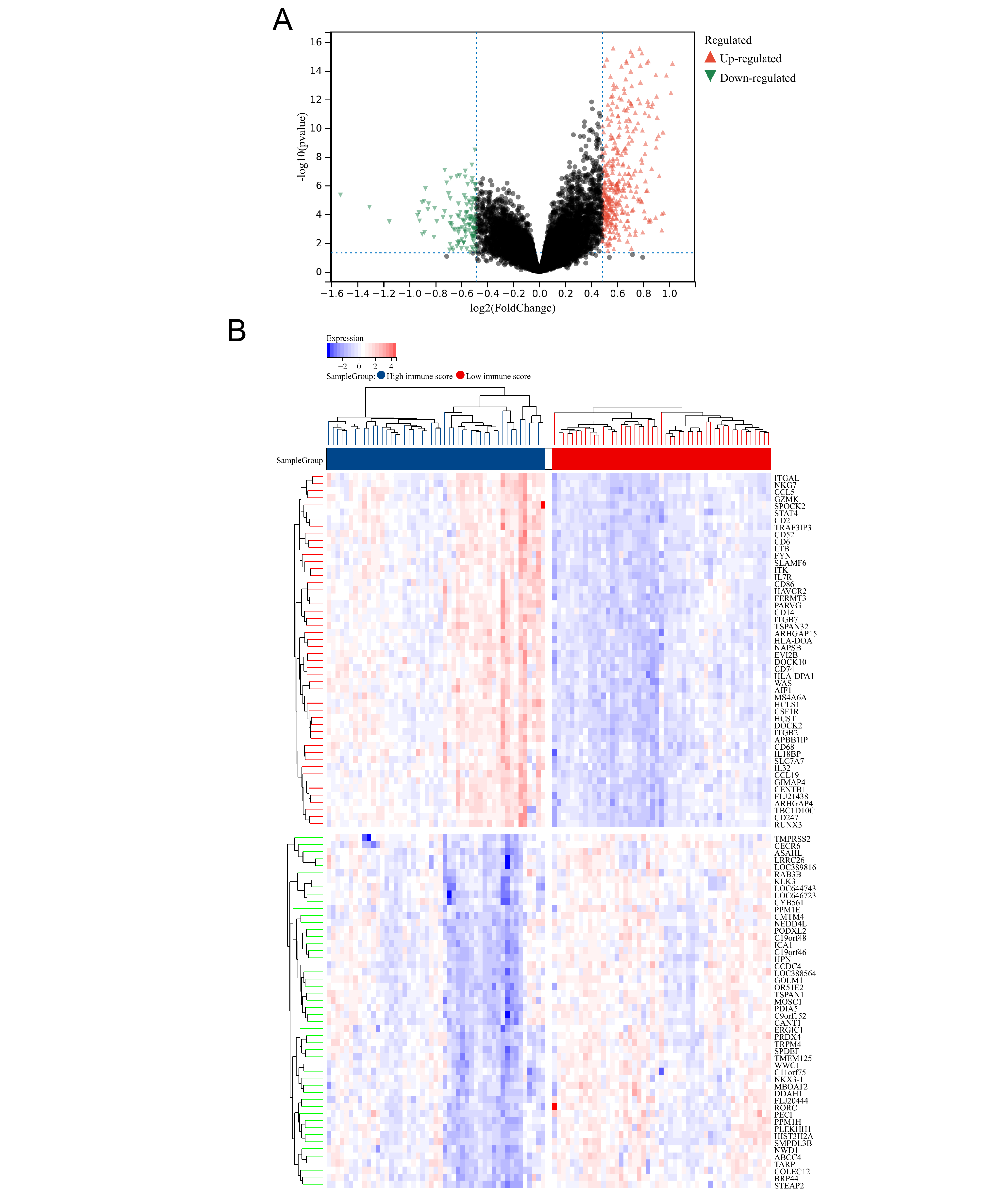

Supplement: Supplementary file 6 [file Image_3.tiff]

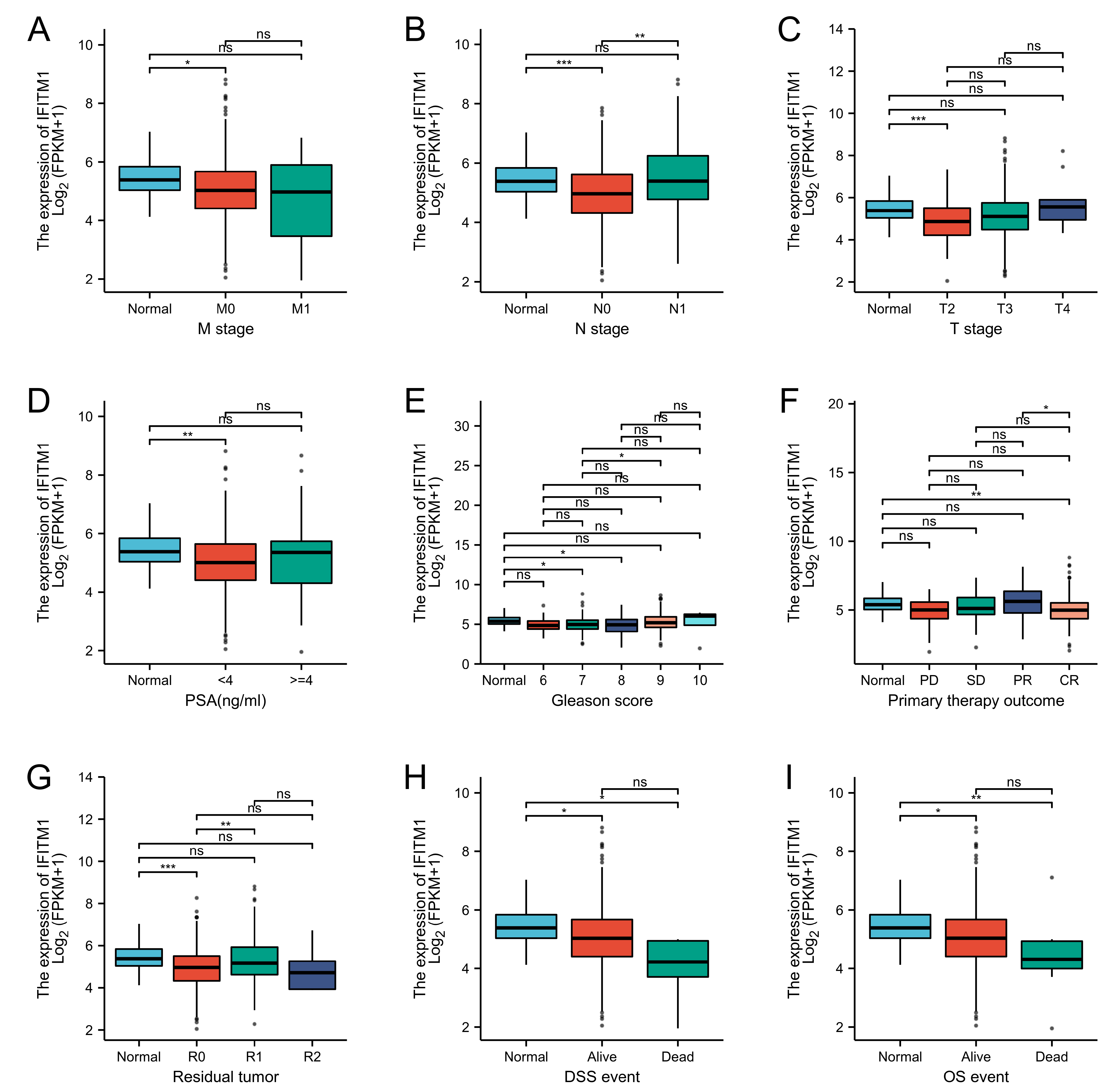

Supplement: Supplementary file 7 [file Image_4.tiff]

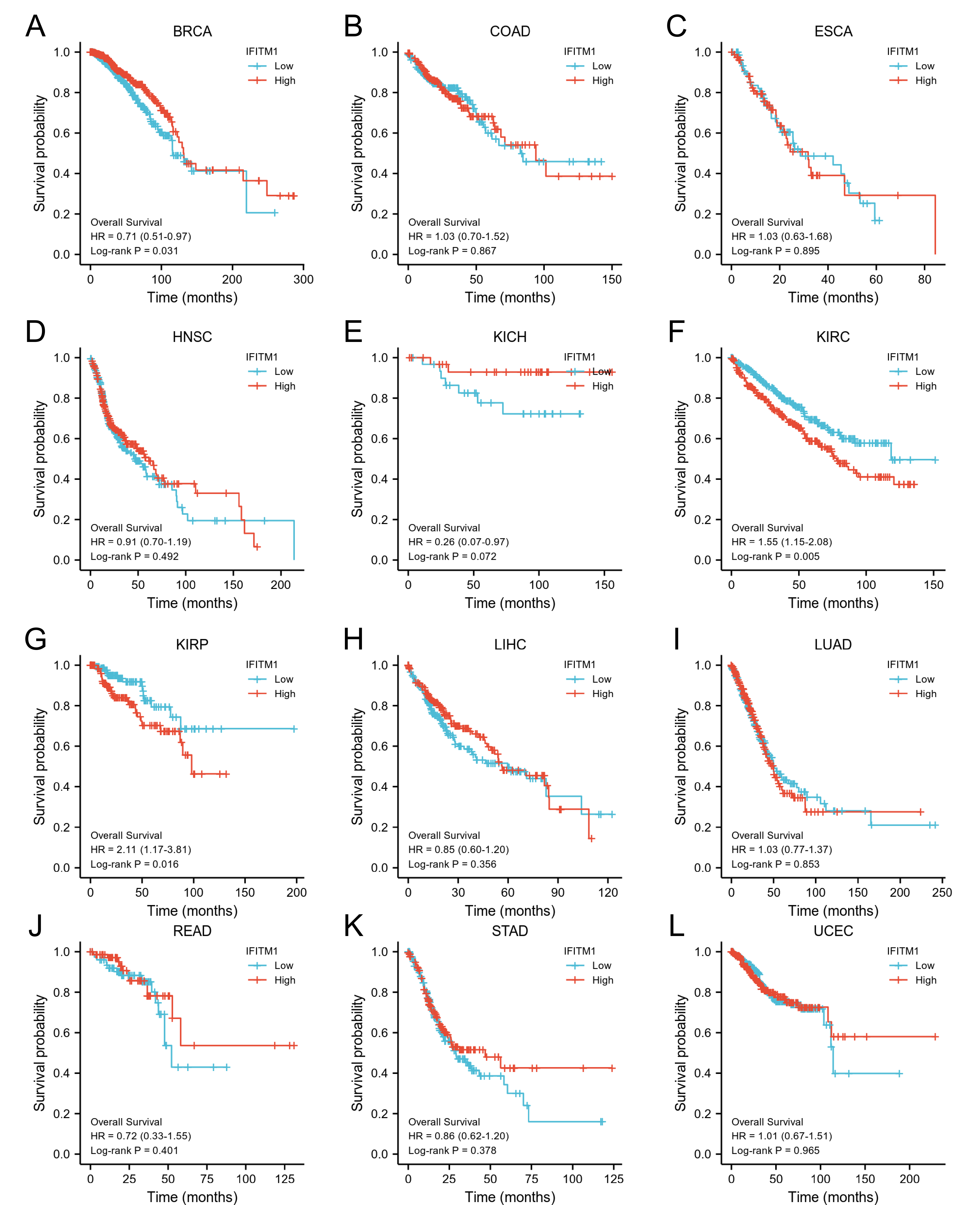

Supplement: Supplementary file 9 [file Image_6.tiff]

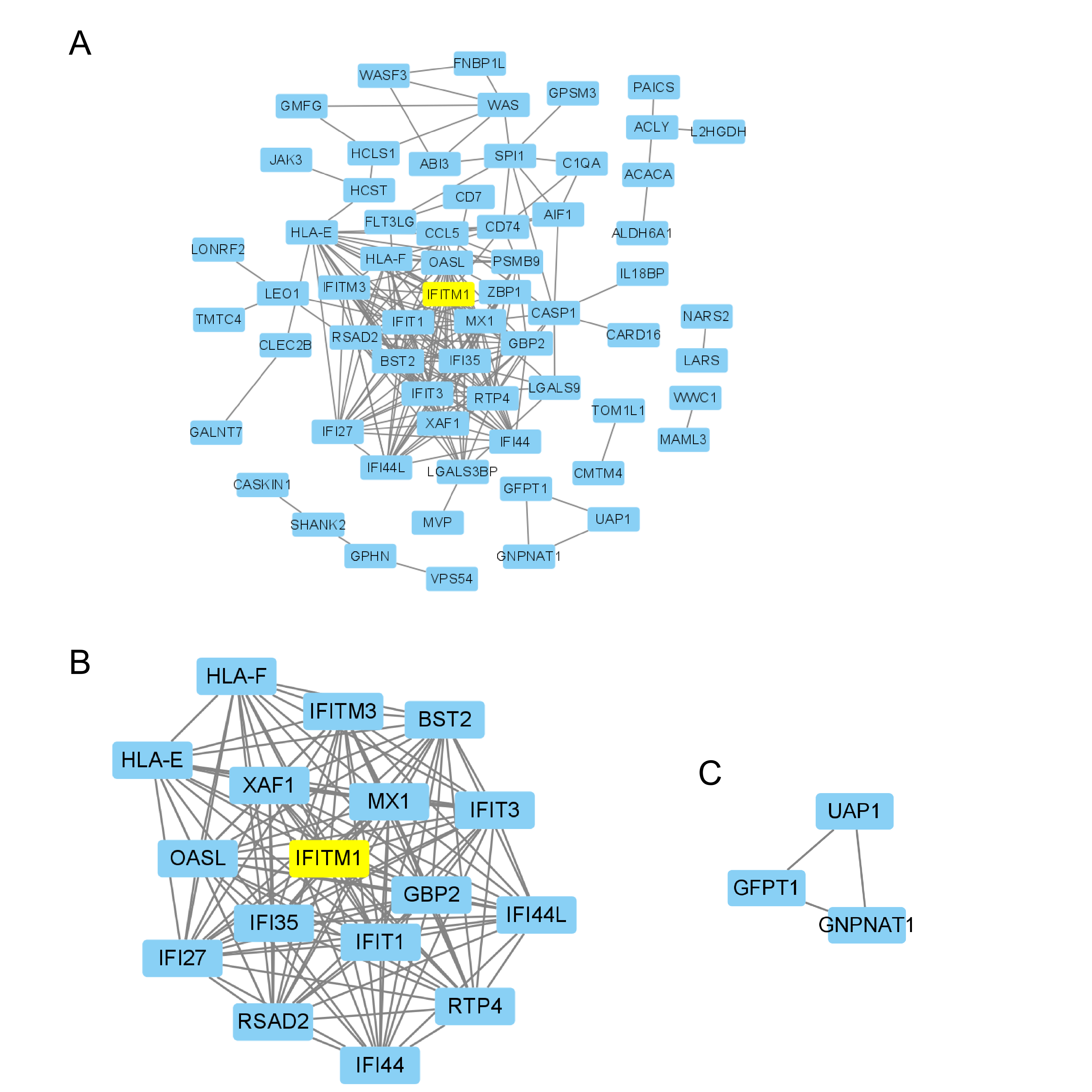

Supplement: Supplementary file 10 [file Image_7.tiff]

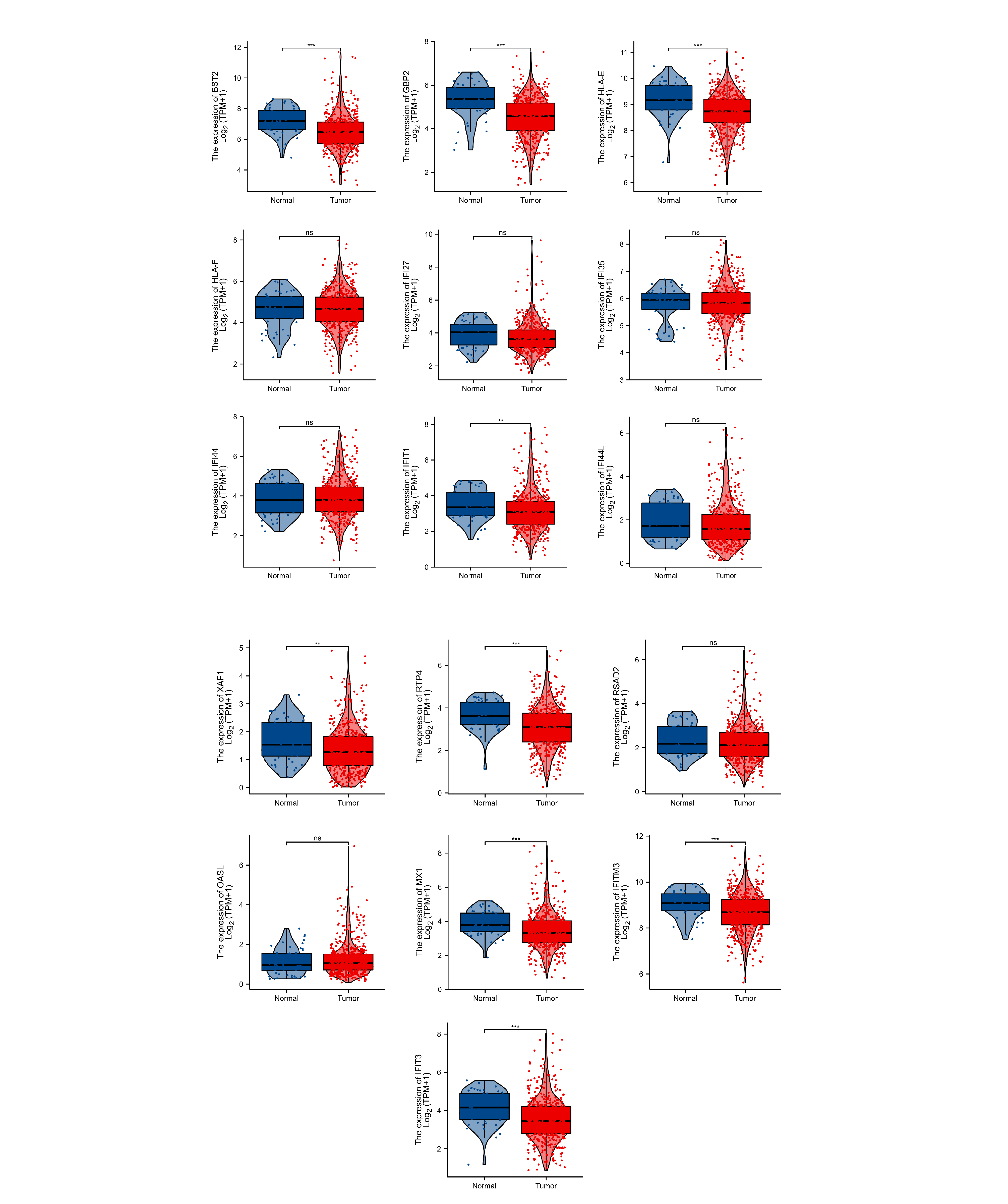

Supplement: Supplementary file 11 [file Image_8.tiff]

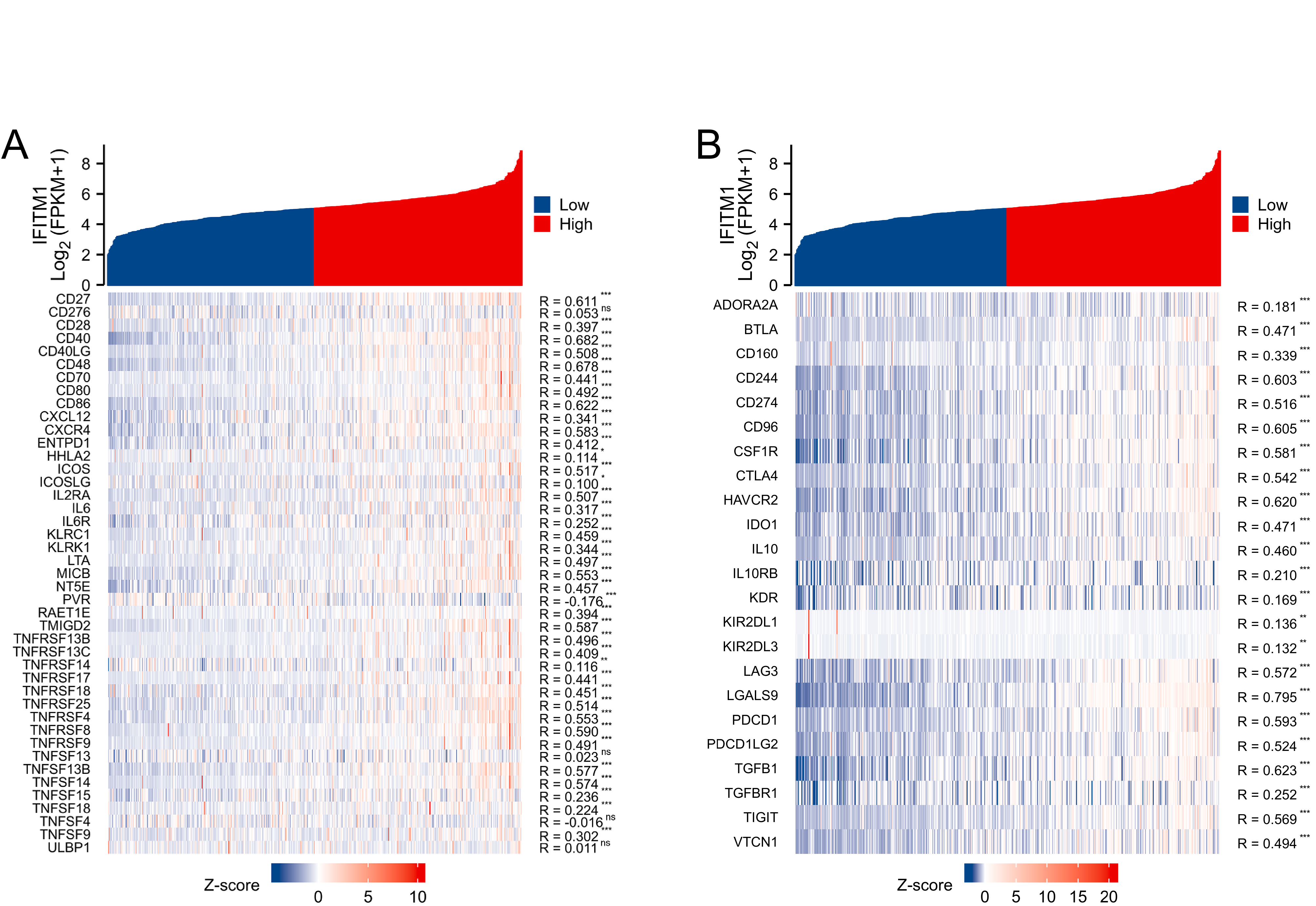

Supplement: Supplementary file 12 [file Image_9.tiff]
