# Supplementary material for: Integrated bioinformatics analysis of IFITM1 as a prognostic biomarker and investigation of its immunological role in prostate adenocarcinoma
Source: Front Oncol. 2022 Dec 14;12:1037535. doi: 10.3389/fonc.2022.1037535 (PMC9795034; doi:10.3389/fonc.2022.1037535)
Supplement: Supplementary file 3 [file Table_1.docx]

**Table S1.** Sequences of primer used quantitative real-time PCR.

| Gene | Forward primer (5' to 3') | Reverse primer (5' to 3') |
| --- | --- | --- |
| IFITM1 | TCGCCTACTCCGTGAAGTCTA | TGTCACAGAGCCGAATACCAG |
| GAPDH | TGAAGGTCGGAGTCAACGG | TGGAAGATGGTGATGGGAT |
